# Integrative Multi-Omics and Single-Cell Analysis Reveal THOC3 and THOC7 as Oncogenic RNA Processing Regulators in Lung Adenocarcinoma

**DOI:** 10.7150/ijms.128975

**Published:** 2026-03-09

**Authors:** Sachin Kumar, Chung-Che Wu, Dahlak Daniel Solomon, Meng-Chi Yen, I-Jeng Yeh, Ching-Chung Ko, Do Thi Minh Xuan, Kai-Fu Chang, Hui-Ru Lin, Hung-Yun Lin, Chia-Lung Shih, Jian-Bin Chen, Wei-Yi Lee, Chih-Yang Wang, Yung-Kuo Lee, Ngoc Uyen Nhi Nguyen

**Affiliations:** 1 PhD Program for Cancer Molecular Biology and Drug Discovery, College of Medical Science and Technology, Taipei Medical University, Taipei 11031, Taiwan.; 2 Graduate Institute of Cancer Biology and Drug Discovery, College of Medical Science and Technology, Taipei Medical University, Taipei 11031, Taiwan.; 3 Faculty of Applied Sciences and Biotechnology, Shoolini University of Biotechnology and Management Sciences Himachal Pradesh 173229, India.; 4 Department of Neurosurgery, Taipei Medical University Hospital, Taipei, Taiwan.; 5 Department of Surgery, School of Medicine, College of Medicine, Taipei Medical University, Taipei, Taiwan.; 6 Neuroscience Research Center, Taipei Medical University, Taipei, Taiwan.; 7 Yogananda School of AI Computers and Data Sciences, Shoolini University Solan 173229, India.; 8 Graduate Institute of Clinical Medicine, College of Medicine, Kaohsiung Medical University, Kaohsiung 80708, Taiwan.; 9 Department of Emergency Medicine, Kaohsiung Medical University Hospital, Kaohsiung Medical University, Kaohsiung 80708, Taiwan.; 10 Department of Medical Imaging, Chi-Mei Medical Center, Tainan 710402, Taiwan.; 11 Department of Health and Nutrition, Chia Nan University of Pharmacy and Science, Tainan 71710, Taiwan.; 12 School of Medicine, College of Medicine, National Sun Yat-Sen University, Kaohsiung 80424, Taiwan.; 13 Van Lang University, 69/68 Dang Thuy Tram Street, Binh Loi Trung Ward, Ho Chi Minh City, Vietnam.; 14 School of Medicine, National Defense Medical University, Taipei 11490, Taiwan.; 15 Medical Laboratory, Medical Education and Research Center, Kaohsiung Armed Forces General Hospital, National Defense Medical University, Kaohsiung 80284, Taiwan.; 16 Institute of Medical Science and Technology, National Sun Yat-Sen University, Kaohsiung 80424, Taiwan; 17 Nursing Department, Kaohsiung Armed Forces General Hospital, National Defense Medical University, Kaohsiung, 80284, Taiwan.; 18 TMU Research Center of Cancer Translational Medicine, Taipei Medical University, Taipei, 11031, Taiwan.; 19 Cancer Center, Wan Fang Hospital, Taipei Medical University, Taipei 11031, Taiwan.; 20 Traditional Herbal Medicine Research Center of Taipei Medical University Hospital, Taipei Medical University, Taipei 11031, Taiwan.; 21 Pharmaceutical Research Institute, Albany College of Pharmacy and Health Sciences, Rensselaer, NY 12144, USA.; 22 Clinical Research Center, Ditmanson Medical Foundation Chiayi Christian Hospital, Chiayi City 60002, Taiwan.; 23 Department of Radiology, Ditmanson Medical Foundation Chia-Yi Christian Hospital, Chiayi, Taiwan.; 24 Division of Thoracic Surgery, Department of Surgery, Kaohsiung Armed Forces General Hospital, National Defense Medical University, Kaohsiung 80284, Taiwan.; 25 Institute of Aquatic Science and Technology, National Kaohsiung University of Science and Technology, Kaohsiung City, Taiwan.; 26 Division of Experimental Surgery Center, Department of Surgery, Tri-Service General Hospital, National Defense Medical University, Taipei 11490, Taiwan.; 27 Center for Regenerative Medicine, University of South Florida Health Heart Institute, Tampa, FL 33602, USA.; 28 Division of Cardiology, Department of Internal Medicine, Morsani School of Medicine, University of South Florida, Tampa, FL 33602, USA.

**Keywords:** lung adenocarcinoma, THOC3, THOC7, RNA export, TREX complex, DNA repair, immune microenvironment, biomarker, therapy resistance

## Abstract

Lung adenocarcinoma (LUAD) remains a leading cause of cancer-related mortality worldwide. Although the transcription-export (TREX) complex plays a central role in RNA maturation and nuclear export, the clinical and biological relevance of individual THO Complex Subunit (including THOC1, THOC2, THOC3, THOC5, THOC6, and THOC7) in LUAD is not well defined. We performed integrative analyses combining bulk transcriptomics from TCGA/GTEx and independent GEO cohorts, survival modeling, DNA methylation profiling, protein-level annotation from public resources, protein-protein interaction network analysis, immune infiltration estimation (TIMER), and single-cell RNA sequencing (scRNA-seq) to evaluate the relevance of THOC3 and THOC7 in LUAD. Across TCGA and external GEO validation datasets, THOC3 and THOC7 were consistently upregulated in LUAD and associated with poorer overall and disease-free survival, whereas other THO complex members showed weaker or inconsistent associations. Given these comparatively consistent and reproducible signals, we therefore prioritized THOC3 and THOC7 for downstream multi-layer analyses. Epigenetic profiling and interaction network analyses placed both genes within conserved RNA processing and export programs linked to genome maintenance pathways. Single-cell transcriptomic analysis provided additional resolution, demonstrating predominant enrichment of THOC3 and THOC7 in malignant epithelial clusters, with THOC3 aligning with transcriptional programs associated with DNA replication and repair, and THOC7 with proliferative and checkpoint-related states. Notably, expression of both genes was also detectable in myeloid and neutrophil subsets, and THOC7 expression remained elevated in recurrent LUAD samples, indicating association with aggressive and treatment-resistant disease states. Collectively, by integrating bulk, single-cell, epigenetic, and immune profiling across multiple independent cohorts, this study identifies THOC3 and THOC7 as reproducible molecular correlates of aggressive LUAD phenotypes. These highlight dysregulated RNA export programs as potential biomarkers of poor prognosis and motivate future functional studies to assess RNA export dependencies in LUAD.

## Introduction

Lung cancer remains the leading cause of cancer-related morbidity and mortality worldwide, accounting for approximately 2.2 million new cases and 1.8 million deaths annually according to GLOBOCAN 2020 estimates 1. It is broadly classified into small-cell lung cancer (SCLC) and non-small cell lung cancer (NSCLC), the latter comprising nearly 85% of all cases 2. Among NSCLC subtypes, lung adenocarcinoma (LUAD) is the most prevalent, accounting for approximately 40% of diagnoses 3. LUAD, originating from alveolar epithelial cells, and represents the most common histological subtype of NSCLC 4. It is the predominant lung cancer subtype in non-smokers and female patients, accounting for 40-50% of cases worldwide 5. Smoking (including passive exposure), air pollution, and occupational hazards remain major risk factors, while epidermal growth factor receptor (EGFR) mutations represent one of the most frequent molecular alterations, particularly in East Asian populations 6-8. Currently, low-dose computed tomography (LDCT), is widely used for early detection, while tissue biopsy obtained via bronchoscopy or needle biopsy remains the gold standard for pathological classification 9. Treatment strategies are guided by disease stage and molecular features; surgery however, prognosis for advanced LUAD remains poor, with a five-year survival rate below 10% for stage IV disease 10, 11. Despite advances in targeted therapies and immunotherapy, therapeutic resistance and disease recurrence continue to pose major clinical challenges 12-14.

**Figure 1:**
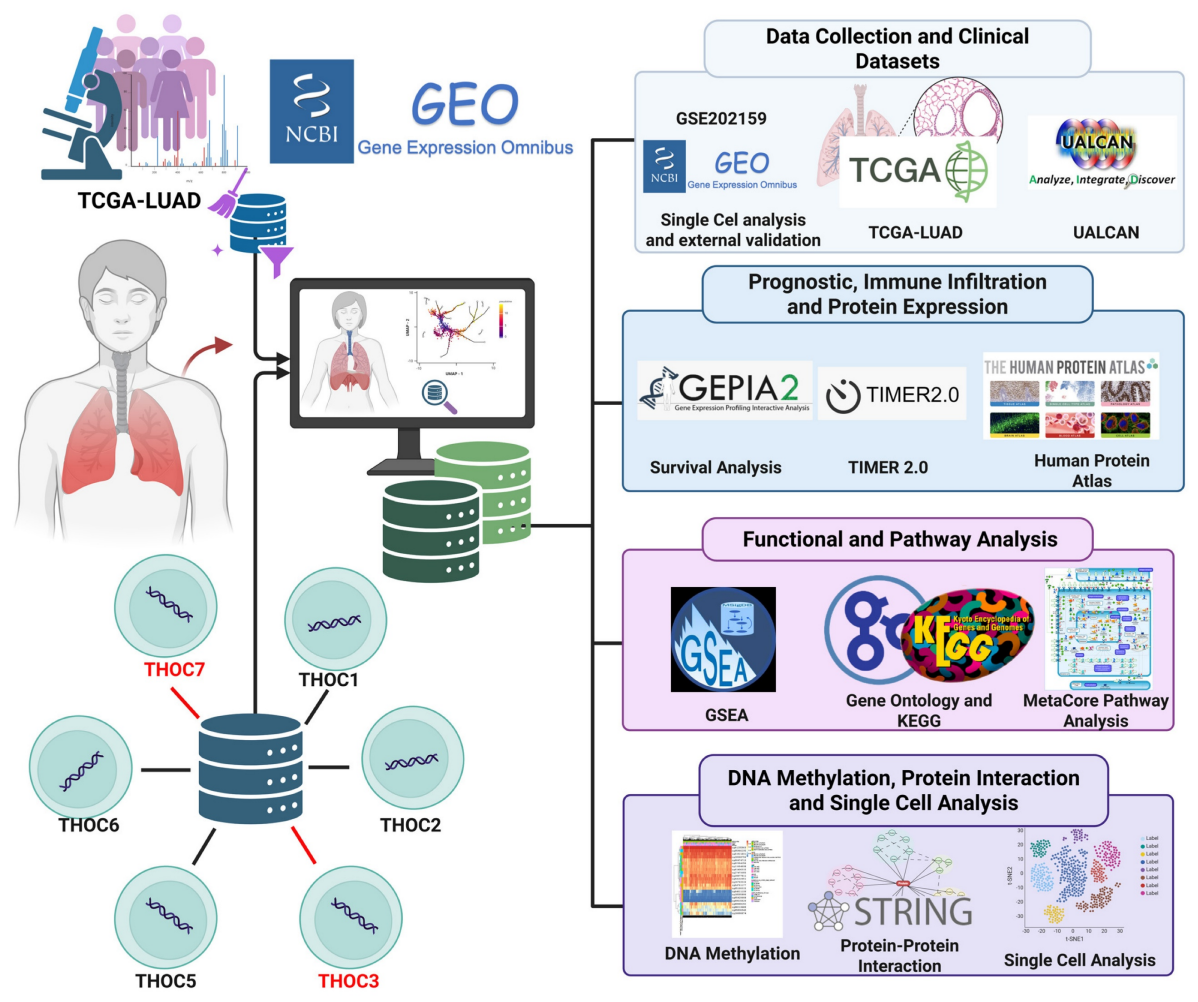
** Schematic overview of the study workflow.** Public datasets from TCGA, GEO, and UALCAN were used to analyze the expression and clinical relevance of THOC3 and THOC7 in LUAD. Prognostic, immune infiltration, and protein validation analyses were performed using GEPIA2, TIMER2.0, and the Human Protein Atlas. Functional enrichment was assessed via GSEA, GO, KEGG, and MetaCore. STRING was used for protein-protein interactions, and single-cell RNA sequencing (scRNA-seq) data provided cell-type-specific insights. Together, these analyses outline the oncogenic and functional landscape of THOC3 and THOC7 in LUAD.

RNA processing and nuclear export are essential components of eukaryotic gene expression, ensuring that pre-mRNAs are properly and accurately spliced, packaged, and transported from the nucleus to the cytoplasm for translation 15, 16. Following transcription by RNA polymerase II, nascent transcripts undergo multiple maturation steps, including capping, splicing, and 3′ end processing 17. Nuclear export requires coordination assembly of ribonucleoprotein complexes and surveillance mechanisms that preserve transcript fidelity. Dysregulation of these processes has been associated with aberrant RNA accumulation, genomic instability, and altered transcriptional programs observed in cancer. The transcription export (TREX) complex is a conserved multiprotein assembly that couples transcriptional elongation and RNA processing to nuclear export 18. Within this complex, the THO subcomplex (THOC1-7, Table 1) functions as a structural scaffold, bridging mRNA splicing and packaging factors with export adaptors such as ALYREF and the DEAD-box RNA helicase DDX39B 19, 20. Notably, THOC4 is annotated as a pseudogene; thus, we focused our analyses on protein-coding THO subunits with consistent transcript annotations (THOC1-3 and THOC5-7). Through this coordination, the THO/TREX machinery contributes to transcript quality control, genome stability, and regulation of genes involved in proliferation, stress responses, and DNA maintenance 21, 22. Previous studies have suggested that dysregulation of specific THOC family members may be associated with tumor-related phenotypes in certain cancer types 23, 24. For instance, THOC1 has been linked to proliferation and anti-apoptotic signatures in breast and ovarian cancers, while THOC5 has been implicated in hematopoietic malignancies 25-28. However, the role of THOC3 and THOC7 in LUAD remains poorly characterized. In this study (Figure 1), we performed a systematic integrative analysis to examine the expression patterns and clinical associations of THOC family members across cancer types, identifying THOC3 and THOC7 as consistently upregulated in LUAD and associated with adverse clinical outcomes, including overall survival and disease-free survival. Protein-level expression patterns derived from public immunohistochemistry resources further supported differential expression trends in LUAD tissues relative to normal lung samples.

Using an integrative multi-omics framework encompassing pan-cancer transcriptomics, survival analyses, protein expression resources, protein-protein interaction networks 29-31, functional enrichment, external GEO-based validation 32-36, and single-cell transcriptomic profiling 37-41, this study aims to characterize the transcriptional and cellular contexts associated with THOC3 and THOC7 expression in LUAD. Our findings are intended to provide a comprehensive, hypothesis-generating foundation for future functional studies investigating the potential roles of RNA export-associated factors in LUAD biology.

## Material and Methods

### Data acquisition and preprocessing

To comprehensively evaluate the expression patterns and clinical associations of THOC family members in lung adenocarcinoma (LUAD), we integrated multi-omics datasets from several public repositories. RNA sequencing (RNA-seq) expression profiles, DNA methylation data, and corresponding clinical annotations for LUAD were obtained from The Cancer Genome Atlas (TCGA-LUAD), with normal lung tissue controls were retrieved from the Genotype-Tissue Expression (GTEx) project. For external transcriptomic validation, independent LUAD cohorts were obtained from the Gene Expression Omnibus GEO, including GSE13213 and GSE31210 42, 43. GSE13213 consists of LUAD tumor samples with available survival information generated on the Affymetrix Human Genome U133 Plus 2.0 platform, whereas GSE31210 includes LUAD tumor samples together with non-tumor lung tissues, enabling independent tumor-normal expression comparisons and survival analyses. Single-cell RNA sequencing (scRNA-seq) data were obtained from GEO accession GSE202159, comprising 82,991 cells derived from multiple LUAD tumors. Additional prognostic validation was performed using the PRECOG platform 44. RNA-seq data from TCGA and GTEx were normalized to transcripts per million (TPM) or fragments per kilobase per million (FPKM), as appropriate. Batch effects between TCGA and GTEx cohorts were corrected using the ComBat algorithm. Principal component analysis (PCA) was performed to evaluate cohort-driven variance between TCGA-LUAD and GTEx samples prior to batch correction (Supplementary Figure S1A-B). Differential expressions between tumor and normal tissues were assessed using the Wilcoxon rank-sum test, and multiple testing correction was applied using the false discovery rate (FDR), with FDR < 0.05 considered statistically significant 45-47.

### Gene expression, survival analysis and protein validation

Gene expression analyses were performed using GEPIA2 (http://gepia2.cancer-pku.cn/) 48, an interactive web tool that integrates TCGA and GTEx datasets for differential expression, correlation, and survival analysis 49-51. The expression of the THOC gene family (THOC1-7) was compared between LUAD tumors and normal tissues, and THOC3 and THOC7 were prioritized as significantly upregulated members. Prognostic analyses were conducted using Kaplan-Meier curves generated in GEPIA2 and Kaplan-Meier Plotter platform (http://kmplot.com/analysis/) 52. Patients were stratified into high- and low-expression groups using the median expression value as the cutoff, unless otherwise specified. Statistical significance was assessed using log-rank tests, hazard ratios (HRs) with 95% confidence intervals (Cls) were estimated using Cox proportional hazards regression models 53, 54. Where available, multivariate analyses incorporated clinical covariates including age, tumor stage, smoking history, and TP53 mutation status. Protein-level expression patterns of THOC3 and THOC7 were examined using the Human Protein Atlas (HPA; https://www.proteinatlas.org/) 55, which provides immunohistochemistry (IHC) and immunofluorescence images derived from patient-derived tissues. Subgroup-specific expression trends based on sex, age, smoking status, tumor stage, and TP53 mutation status were further explored using UALCAN (http://ualcan.path.uab.edu/) 56, which integrates TCGA transcriptomic data and Clinical Proteomic Tumor Analysis Consortium (CPTAC) protein datasets. These protein-level analyses were used to provide descriptive expression context rather than experimental validation.

### DNA methylation and protein-protein interaction (PPI) network analysis

Epigenetic regulation of THOC3 and THOC7 was assessed using Illumina HumanMethylation450K array data from TCGA-LUAD. CpG probes mapped to promoter regions, gene bodies, and N-shore regions were analyzed, and differences between tumor and normal tissues were evaluated using Student's t-tests, with significance defined as mean Δβ ≥ 0.20 and p < 0.01 57. Protein-protein interaction (PPI) networks were constructed using STRING v11.5 (https://string-db.org/) 58, incorporating experimentally validated and predicted interactions with confidence scores. Networkswere visualized using Cytoscape v3.9.1 and hub genes were identified using CytoHubba, based on degree and betweenness centrality 59-61. Densely connected functional modules were extracted using MCODE. These analyses were performed to characterize interaction contexts and network connectivity rather than to infer direct mechanistic regulation.

### Functional enrichment analysis

Genes co-expressed with THOC3 and THOC7 were identified via cBioPortal (https://www.cbioportal.org) selecting the top 10% of genes ranked by Pearson correlation coefficients 62. Functional annotation was conducted using the clusterProfiler R package, to evaluate Gene Ontology (GO) biological processes, cellular components, and molecular functions and Kyoto Encyclopedia of Genes and Genomes (KEGG) pathways 63. Gene Set Enrichment Analysis (GSEA) was performed using the Molecular Signatures Database (MSigDB v7.5) with a pre-ranked approach and 1,000 permutations 64-66. Pathways with nominal p-values < 0.05 and false discovery rate (FDR) q-values < 0.25 were considered significant 67. To complement these analyses, pathway enrichment was further evaluated using MetaCore (Clarivate Analytics), a curated systems biology platform that integrates experimentally supported molecular interactions 68-70. MetaCore results were used to refine pathway associations and are reported in the Supplementary Tables.

### Immune infiltration and single-cell RNA sequencing (scRNA-seq) analysis

The correlation between THOC3/THOC7 expression and immune cell infiltration were evaluated using the TIMER2.0 platform (https://timer.cistrome.org). Immune lineages analyzed included B cells, CD4⁺ T cells, CD8⁺ T cells, macrophages, dendritic cells, and neutrophils. Correlation coefficients were calculated using Spearman's correlation, with p < 0.05 considered statistically significant after FDR correction 71.

Single-cell RNA-seq data from GSE202159, were analyzed using Seurat v5.1.0. Cells were filtered based on standard quality-control criteria, normalized, and subjected to dimensionality reduction using principal component analysis (PCA), uniform manifold approximation and projection (UMAP), and t-distributed stochastic neighbor embedding (t-SNE). Cell clustering was performed using the Louvain algorithm, and cell types were annotated based on established canonical markers for epithelial cells, fibroblasts, endothelial cells, myeloid cells, T cells, B cells, neutrophils, and lymphatic endothelial cells 72-74. THOC3 and THOC7 expression patterns were visualized across cell populations using features and violin plots. Subcluster analyses stratified by smoking history, TP53 mutation status, and recurrence status were performed to explore context-dependent expression patterns 75.

### Statistical analysis

All statistical analyses were performed using R version 4.3.0. Tumor-normal expression differences were evaluated using the Wilcoxon rank-sum test, and subgroup analyses were conducted with appropriate multiple-testing correction using the false discovery rate. Survival outcomes were assessed using KaplanMeier analysis with log-rank testing, and Cox proportional hazards regression models were applied to estimate hazard ratios and 95% confidence intervals, adjusting for clinical covariates where available. DNA methylation analyses used Student's t-tests with predefined Δβ thresholds. Immune infiltration correlations were assessed using Spearman's correlation. Functional enrichment analyses employed hypergeometric and permutation-based testing as implemented in clusterProfiler, GSEA, and MetaCore. Visualizations were generated using ggplot2, Seurat, ComplexHeatmap, and Cytoscape as we previous described 76-79.

## Results

### Expression patterns and prognostic associations of THOC family members in LUAD

**Figure 2:**
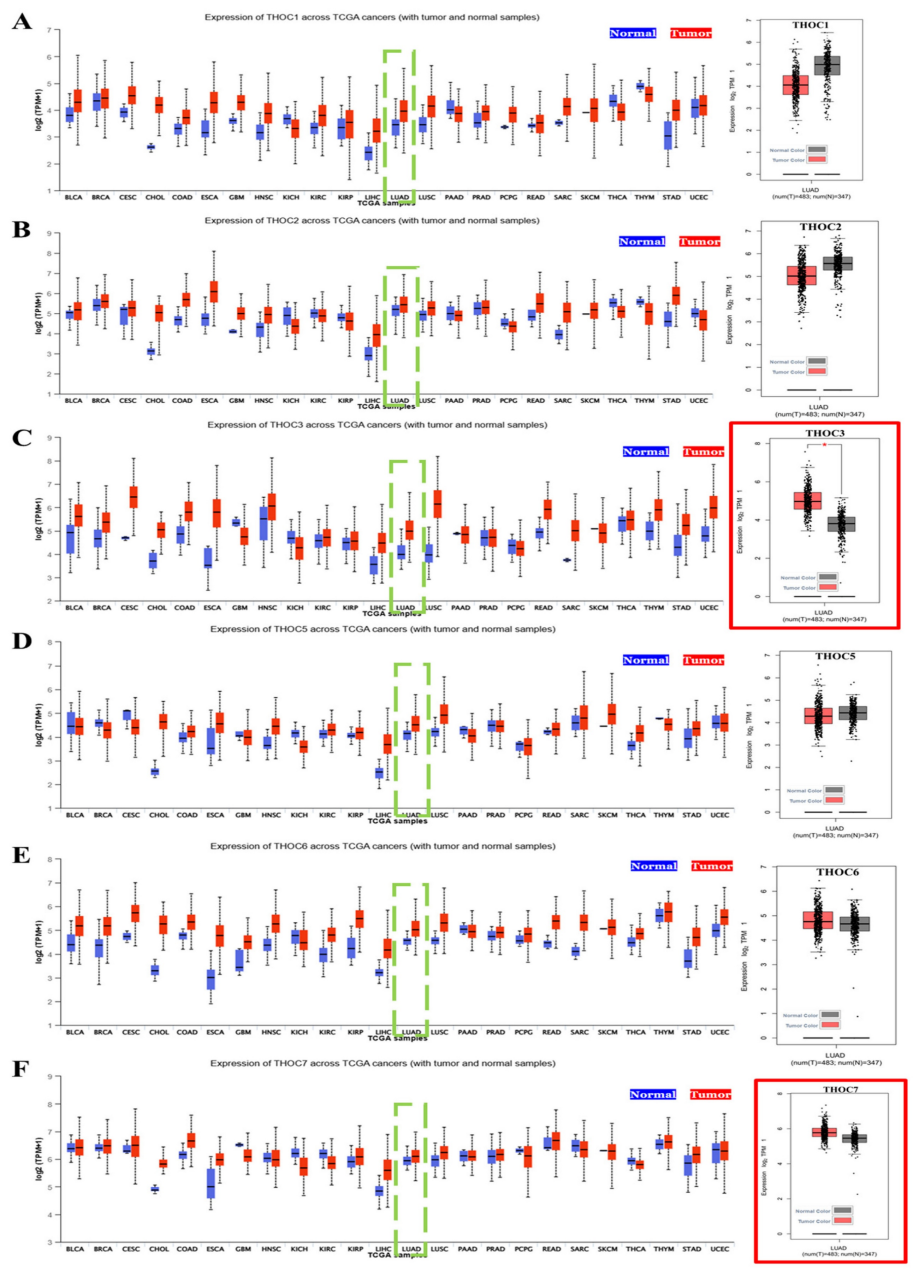
**Expression of THOC family genes across TCGA cancers and in LUAD**. **(A-F)** Boxplots showing the mRNA expression levels (log2 TPM) of THOC1-THOC7 family members across TCGA pan-cancer datasets. Tumor tissues are represented in red and matched normal tissues in blue. Green dashed boxes highlight lung adenocarcinoma (LUAD) samples for each THOC gene. The corresponding right-hand panels show LUAD tumor samples (red) versus normal lung tissues (black). Notably, THOC3 (C) and THOC7 (F) display significantly higher expression in LUAD tumors compared with normal tissues, as indicated by red-outlined panels. Colors: Red = tumor samples; Blue = pan-cancer normal tissues; Black = LUAD normal tissues.

In this study, we first examined the expression patterns of the THOC family gene across human cancers using TCGA pan-cancer datasets. Among the seven canonical subunits of the THO complex (THOC1-7), THOC3 and THOC7 showed the most consistently elevation in LUAD relative to normal lung tissues (Figure 2A-F). Specifically, both THOC3 (Figure 2C) and THOC7 (Figure 2F), exhibited significantly higher median mRNA expression in LUAD tumor tissues compared with adjacent normal lung tissues (Wilcoxon test, p < 0.01). However, distributions showed substantial overlap between tumor and normal samples, indicating modest effect sizes rather than uniform tumor-specific overexpression. In contrast, other THOC members including THOC1, THOC2, THOC5, and THOC6 (Figure 2A, 2B, 2D, 2E), displayed weaker or non-significant tumor-normal differences. These results suggest that while multiple THOC genes are expressed in LUAD, THOC3 and THOC7 demonstrate relatively stronger, though heterogeneous, elevation at the cohort level. To determine whether THOC gene expression varied across disease progression, we further analyzed expression levels across clinical stages I-IV in the TCGA-LUAD cohort. As shown in Supplementary Figure S1C-H, none of the THOC family members exhibited statistically significant stage-dependent differences by ANOVA. Although THOC3 displayed a modest trend toward higher expression in more advanced stages, substantial overlap across stages was observed, highlighting pronounced intra-stage heterogeneity. These findings indicate that THOC3 and THOC7 expression is not strongly stratified by clinical stage, and that their tumor-normal differences are not solely driven by stage progression.

**Figure 3:**
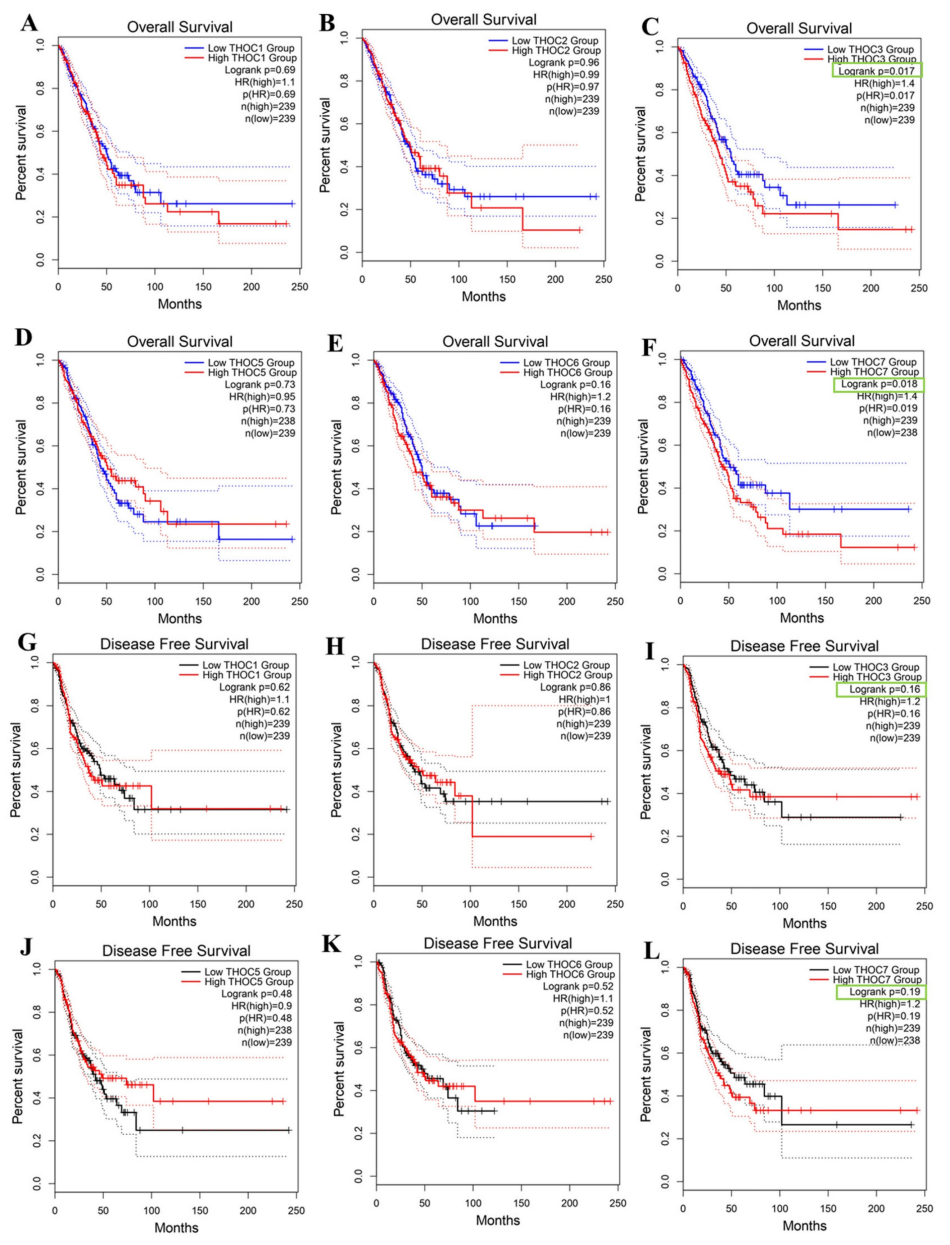
**Prognostic impact of THOC family gene expression in LUAD. (A-F)** Overall survival (OS) analysis of LUAD patients stratified into low- and high-expression groups for THOC1-THOC7 using Kaplan-Meier plots. Patients with high THOC3 expression (C) showed significantly poorer OS compared with the low-expression group. Similarly, high THOC7 expression (F) was strongly associated with unfavorable OS. No statistically significant differences were observed for THOC1, THOC2, THOC4, THOC5, and THOC6. **(G-L)** Disease-free survival (DFS) analysis of LUAD patients stratified by THOC1-THOC7 expression. Elevated THOC3 expression (I) correlated with shorter DFS, and THOC7 high expression (L) also predicted significantly worse DFS. The remaining THOC family members did not show significant DFS associations (G, H, J, K). Colors: Red = high-expression group; Blue = low-expression group (OS panels); Black = low-expression group (DFS panels).

We next assessed whether THOC gene expression was associated with clinical outcomes in LUAD patients using Kaplan-Meier survival analyses in the TCGA-LUAD cohort. Overall survival (OS), and disease-free survival (DFS) were evaluated using median expression cutoffs. For most THOC family members, survival associations were weak or inconsistent (Figure 3A, 3B, 3D, 3E, 3G, 3H, 3J, 3K). In contrast, higher expressions of THOC3 and THOC7 were associated with poorer survival outcomes. Specifically, patients with high THOC3 expression showed reduced OS (Figure 3C, log-rank p = 0.017, HR = 1.34) and DFS (Figure 3I, p = 0.018, HR = 1.29), while elevated THOC7 expression was similarly associated with shorter OS (Figure 3F, p = 0.017, HR = 1.41) and DFS (Figure 3L, p = 0.019, HR = 1.29). These associations reflect univariate correlations and do not establish independent prognostic value or causal involvement. These findings indicate that THOC3 and THOC7 are among the THOC family members most consistently associated with altered expression and adverse clinical outcomes in LUAD relative to other subunits. Importantly, these results describe statistical associations observed at the cohort level, characterized by heterogeneity and overlapping expression distributions, and do not imply that THOC3 or THOC7 directly drive tumor progression. Rather, their elevated expression may reflect broader transcriptional states associated with tumor aggressiveness or proliferative demand. Based on these association patterns, THOC3 and THOC7 were prioritized for further descriptive and integrative analyses in subsequent sections of the study.

### Protein-level context, external transcriptomic validation, and clinicopathological associations of THOC3 and THOC7 in LUAD

**Figure 4:**
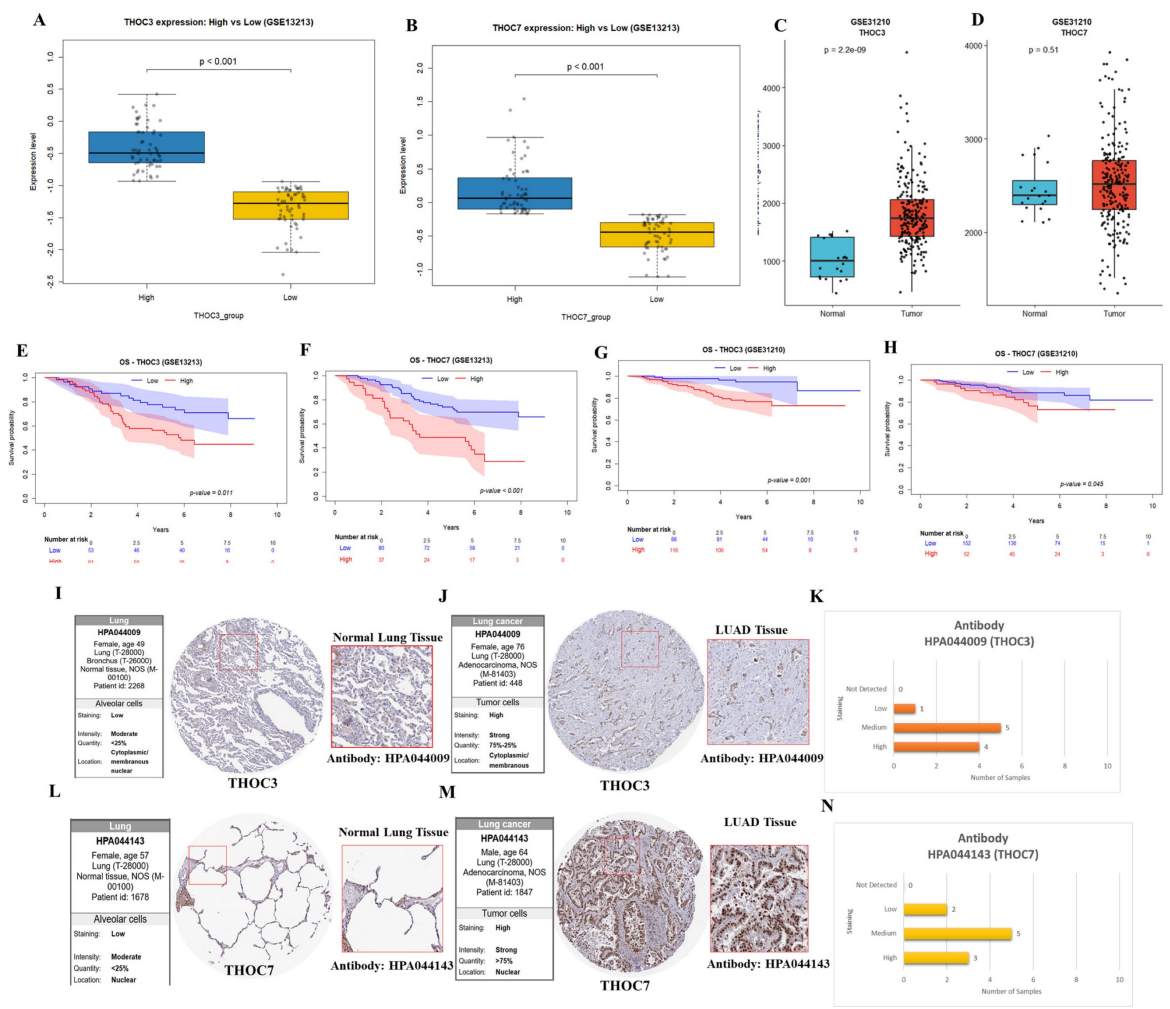
** External validation of THOC3 and THOC7 expression, prognostic relevance, and protein-level distribution in LUAD. (A-D)** Box-plot analysis showing differential expression of THOC3 and THOC7 between high- and low-expression groups in the independent LUAD cohort GSE13213 (A-B) and GSE31210 (C-D), confirming significantly elevated expression in the high-expression groups (p < 0.001). **(E-H)** Kaplan-Meier overall survival analyses demonstrating that high expression of THOC3 and THOC7 is associated with poorer overall survival in LUAD patients from GSE13213 (E-F) and the independent GSE31210 cohort (G-H). **(I-J)** Representative immunohistochemical (IHC) staining of THOC3 protein (antibody HPA044009) in normal lung alveolar tissue (I) and LUAD tumor tissue (J). Normal lung samples show weak to moderate cytoplasmic and membranous staining, whereas LUAD tissues display strong cytoplasmic/membranous staining in the majority of tumor cells. **(K)** Quantitative summaries of IHC staining intensity derived from the Human Protein Atlas, indicating predominantly medium-to-high expression levels of THOC3 in LUAD tissues.** (L-M)** Representative IHC staining of THOC7 protein (antibody HPA044143) in normal lung alveolar tissue (L) and LUAD tumor tissue (M). Normal tissues exhibit weak to moderate nuclear staining, while LUAD samples demonstrate strong nuclear localization with high tumor cell positivity. **(N)** Quantitative summaries of IHC staining intensity derived from the Human Protein Atlas, indicating predominantly medium-to-high expression levels of THOC7 LUAD tissues.

Building on the observation that THOC3 and THOC7 exhibit relatively higher mRNA expression in LUAD and are associated with unfavorable overall survival (OS) and disease-free survival (DFS) in the TCGA-LUAD cohort (Figure 2-3), we next sought to evaluate the reproducibility of these associations in independent datasets and to place the findings within a protein-level and clinicopathological context. To address concerns, we performed external transcriptomic validation using two independent LUAD cohorts from the Gene Expression Omnibus (GEO), GSE13213 and GSE31210. Consistent with TCGA results, both THOC3 and THOC7 showed significantly higher expression in the high-expression groups compared with low-expression groups (Figure 4A-D) in both the independent LUAD cohort. Kaplan-Meier survival analyses further demonstrated that elevated expression of THOC3 and THOC7 was associated with poorer overall survival in both the independent cohort (Figure 4E-H). These findings confirm that the observed expression outcome associations are reproducible across cohorts, supporting robustness of the transcriptomic signal. Importantly, this external validation strengthens confidence in the associations while remaining descriptive and non-mechanistic in nature.

To complement transcriptomic analyses, protein-level expression patterns of THOC3 and THOC7 proteins in were examined using immunohistochemistry (IHC) data from the Human Protein Atlas (HPA). In normal lung tissues, THOC3 and THOC7 generally displayed weak to moderate staining, whereas LUAD tumor tissues more frequently exhibited moderate to strong staining intensity (Figure 4I-N). These observations provide protein-level expression context consistent with mRNA-level findings but do not constitute independent experimental validation. Quantitative summaries derived from HPA further indicated a shift toward medium-to-high expression levels in LUAD samples relative to normal tissues. Immunofluorescence (IF) images from the Human Protein Atlas (HPA) were used to illustrate subcellular distribution patterns of THOC3 and THOC7 and have now been relocated to the Supplementary Materials (Supplementary Figure S2). These images were included solely for descriptive visualization of intracellular localization and not as functional or disease-specific validation. The IF data were generated using A431 cells, which were employed only as an EGFR-high epithelial reference model with well-defined cellular morphology and strong, reproducible fluorescence signals, enabling clear discrimination between nuclear and cytoplasmic compartments. Given that aberrant EGFR signaling is a central molecular feature of lung adenocarcinoma, A431 cells provide a technical context in which EGFR-associated nucleocytoplasmic organization can be readily visualized, rather than a biological surrogate for LUAD. Importantly, A431 cells were not used to model LUAD-specific behavior, and no conclusions regarding lung cancer-specific function, oncogenic mechanism, or EGFR-dependent regulation of THOC3 or THOC7 are derived from these images. As these IF data originate from a non-LUAD epithelial system, they are presented strictly to illustrate general localization tendencies, and no LUAD-specific mechanistic inference is drawn from these observations.

**Figure 5:**
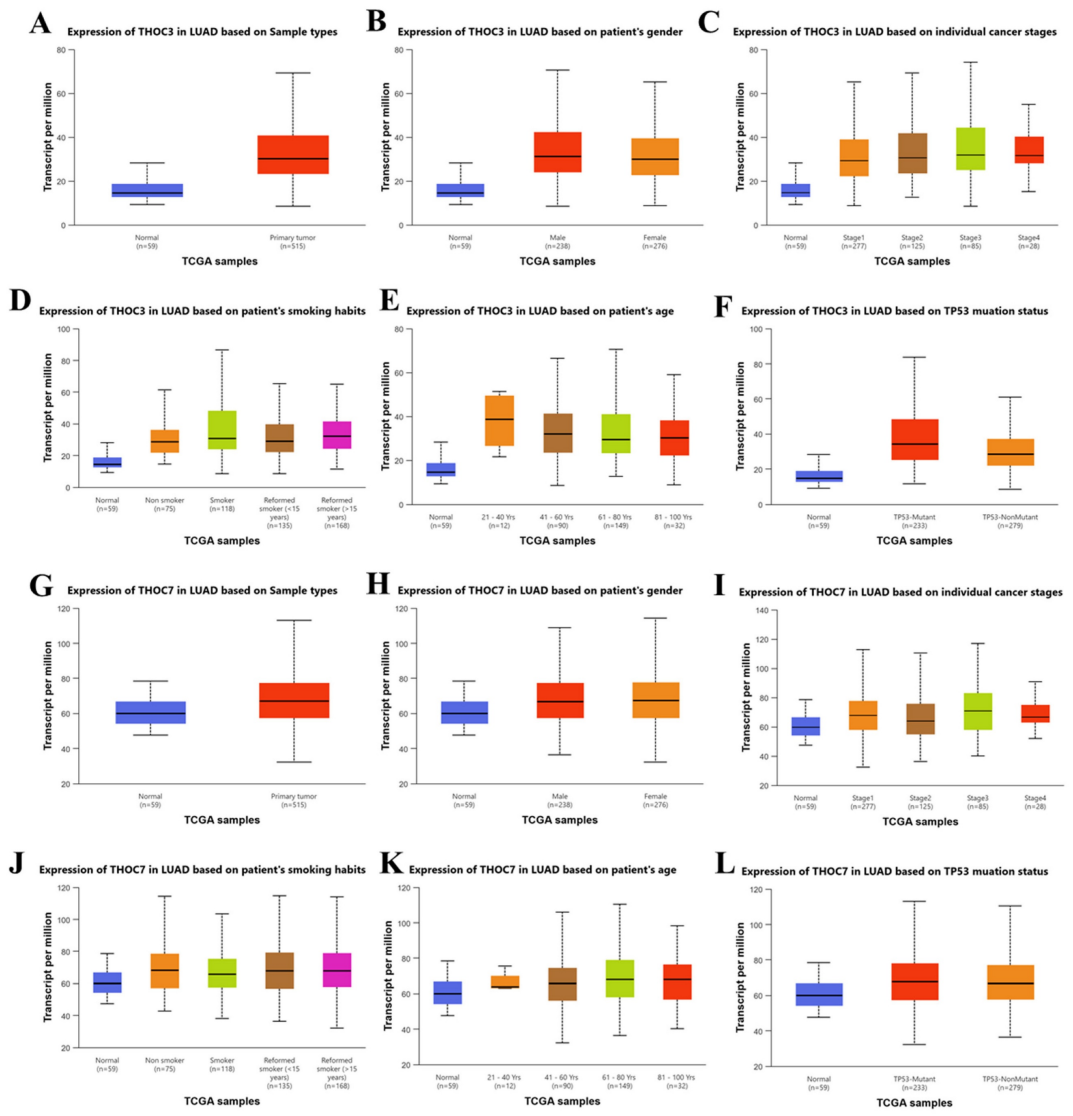
**Clinical correlation of THOC3 and THOC7 expression in LUAD TCGA patients. (A-F)** Boxplots showing THOC3 expression in LUAD based on different clinical parameters, **(A)** Sample types (normal vs. primary tumor),** (B)** patient gender**, (C**) cancer **stages (**I-IV),** (D)** smoking habits (non-smoker, smoker, reformed smoker), **(E)** patient age groups, and** (F)** TP53 mutation status. THOC3 expression was significantly elevated in primary tumor tissues compared with normal controls** (A)** and was further enriched in advanced cancer stages** (C)** and TP53-mutant samples** (F). (G-L)** Boxplots showing THOC7 expression in LUAD across the same clinical categories,** (G)** Sample types,** (H)** gender,** (I)** cancer stages,** (J)** smoking habits,** (K)** age, and** (L)** TP53 mutation status. THOC7 expression was markedly higher in tumor tissues than in normal lung** (G),** with notable increases in TP53-mutant groups** (L).** Colors: Blue = normal tissues; Red/Orange/Brown/Green/Magenta = clinical subgroups as indicated in each panel. Statistical significance: p < 0.05, p < 0.01, p < 0.001 (log2 TPM, TCGA).

To further explore clinical associations, subgroup analyses were performed using TCGA data accessed via UALCAN (Figure 5A-L). Both THOC3 and THOC7 were consistently expressed at higher levels in LUAD tumor tissues compared with normal lung tissues across multiple clinical strata, including sex, age group, and smoking status. Expression levels were elevated across clinical stages I-IV without strong stage-dependent stratification, consistent with the stage-agnostic patterns observed in Supplementary Figure S1C-H. Notably, both genes exhibited higher expression in TP53-mutant tumors compared with TP53 wild-type cases, suggesting an association between THOC3/THOC7 expression and genomic instability-associated tumor contexts rather than linear disease progression. These analyses demonstrate that THOC3 and THOC7 expression-survival associations are reproducible across independent LUAD cohorts, supported by external GEO validation, and are accompanied by consistent protein-level expression patterns in patient tissues. However, all findings presented here remain correlational and do not establish a direct oncogenic or mechanistic role for THOC3 or THOC7. Instead, the data support the interpretation that these genes mark transcriptional states associated with adverse clinical outcomes in LUAD, warranting further functional investigation.

### DNA methylation and Interaction Landscape of THOC3 and THOC7 in LUAD

**Figure 6:**
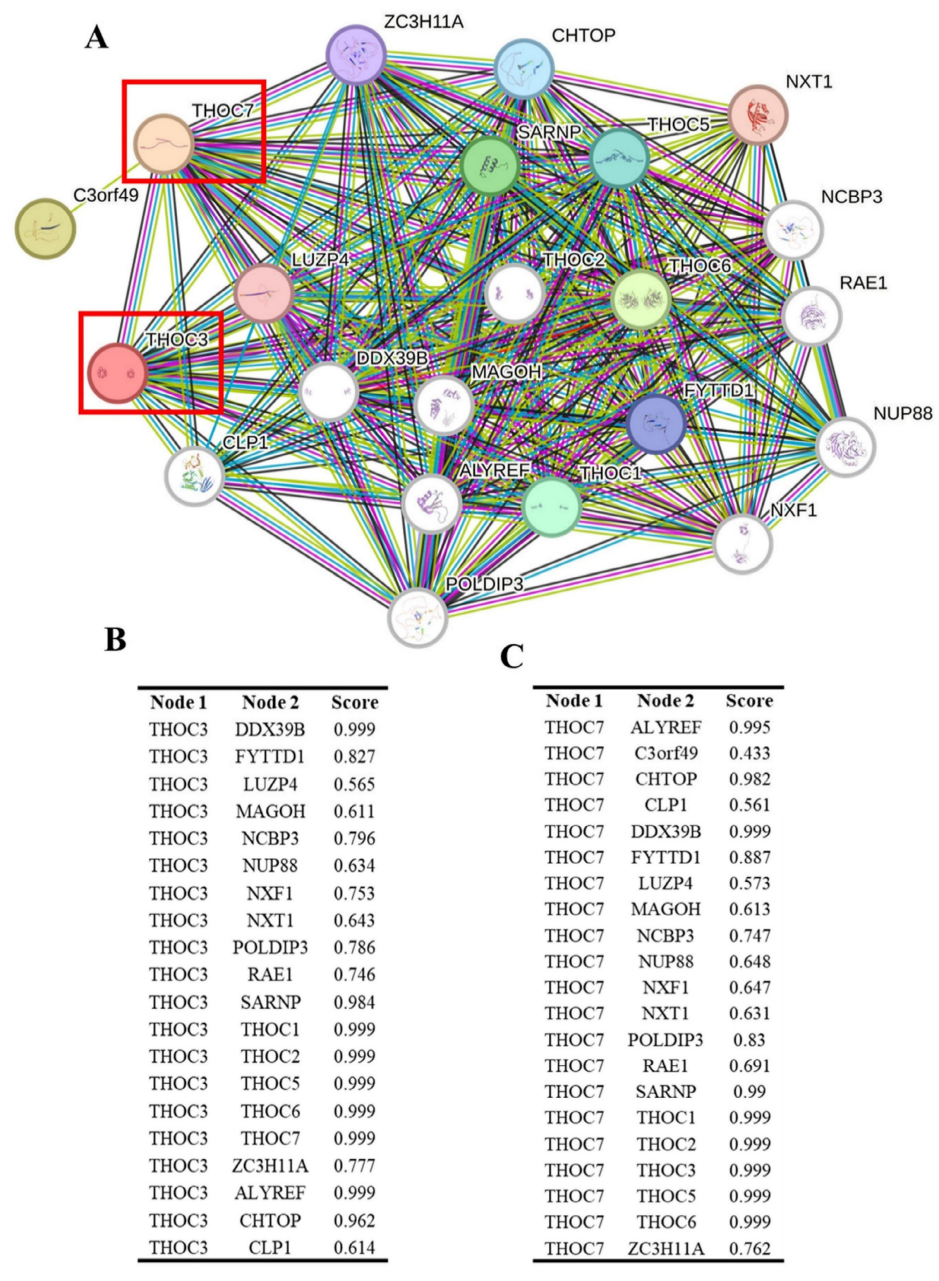
**Protein-protein interaction (PPI) network analysis of THOC3 and THOC7. (A)** STRING-based interaction network of THOC3 and THOC7 with other members of the THO/TREX complex and RNA processing machinery. Nodes represent proteins, and edges indicate functional associations, with thicker/more numerous connections reflecting stronger confidence scores. Both THOC3 and THOC7 (red boxes) display high connectivity with TREX components, including THOC1/2/5/6, ALYREF, DDX39B, MAGOH, NCBP3, and CHTOP, indicating their central roles in RNA splicing and export. **(B-C)** Tabulated interaction scores of THOC3 (B) and THOC7 (C) with their top partner proteins. For THOC3, the strongest interactions were observed with DDX39B, THOC1, THOC2, THOC5, THOC6, and ALYREF (score = 0.999). For THOC7, high-confidence interactions included DDX39B, THOC1/2/3/5/6, ALYREF, and SARNP (score = 0.999). Colors: Nodes = proteins; red box highlights = THOC3 and THOC7.Interaction scores: Based on STRING confidence (0-1.0), with values approaching 1.0 representing strongest predicted associations.

To explore potential regulatory contexts associated with the elevated expression of THOC3 and THOC7 in LUAD, we examined their DNA methylation profiles using TCGA Illumina 450K data. Distinct methylation patterns were observed between LUAD and normal lung tissues (Supplementary Figure S3A-B). For THOC3, two CpG sites (cg11951952 and cg1444436) displayed lower methylation levels in tumor samples compared with normal tissues. For THOC7, three CpG sites (cg11378484, cg22134162, and cg25490800) similarly exhibited reduced methylation across tumor samples, including regions within gene bodies and N-shore regions. These observations indicate an association between altered DNA methylation states and increased THOC3 and THOC7 expression in LUAD. However, given the correlative nature of these data, no direct causal relationship between methylation changes and transcriptional activation can be inferred. To further contextualize THOC3 and THOC7 within known molecular networks, we constructed a protein-protein interaction (PPI) network using STRING (Figure 6A). Both THOC3 and THOC7 were embedded within dense interaction networks enriched for RNA processing and mRNA export factors, consistent with their established roles as components of the THO/TREX complex. High-confidence predicted interactions were observed with canonical TREX-associated proteins, including THOC1, THOC2, THOC5, THOC6, DDX39B, ALYREF, SARNP, and CHTOP. To provide a quantitative summary of these predicted interactions, interaction confidence scores for THOC3 and THOC7 with their top-ranked partners are shown in Figure 6B and Figure 6C, respectively, illustrating their connectivity within the conserved THO/TREX interaction network. For THOC3, strong predicted associations were observed with DDX39B, ALYREF, and SARNP, proteins involved in pre-mRNA processing and nuclear export. Additional interactions with MAGOH and CHTOP place THOC3 within broader RNA maturation and surveillance networks. Similarly, THOC7 displayed predicted interactions with core TREX components as well as nuclear pore-associated proteins such as RAE1 and NUP88, suggesting potential links between RNA export machinery and nuclear transport architecture. These interaction patterns are consistent with known TREX complex organization and do not indicate LUAD-specific rewiring or functional gain. Importantly, PPI networks derived from STRING represent predicted and literature-curated associations and do not establish functional dependency, regulatory hierarchy, or tumor-specific activity. The observed connectivity of THOC3 and THOC7 therefore reflects their participation in conserved RNA-processing pathways rather than direct evidence of oncogenic function. These DNA methylation and PPI analyses provide contextual support for the transcriptional upregulation of THOC3 and THOC7 in LUAD and place these genes within established RNA-processing and export networks. However, all findings presented here are associative and should be interpreted as hypothesis-generating. Functional validation will be required to determine whether altered methylation or network positioning contributes causally to LUAD pathogenesis.

### Functional and Pathway Characterization of THOC3 and THOC7 in LUAD

**Figure 7:**
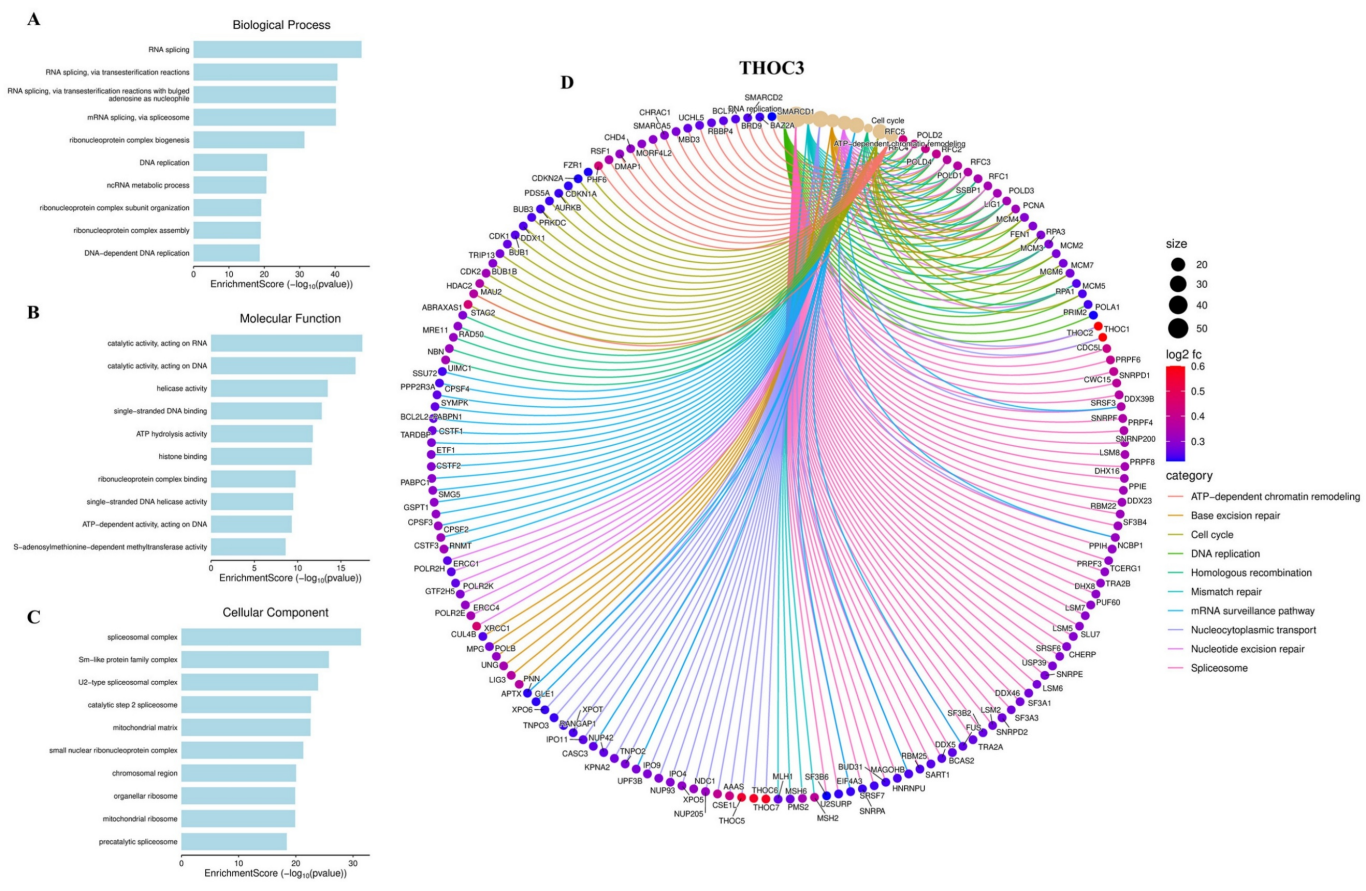
**Functional enrichment and interaction network of THOC3 in LUAD. (A-C)** Gene Ontology (GO) enrichment analysis of THOC3-associated genes in LUAD.(A) Biological process terms revealed enrichment in RNA splicing, RNA transesterification reactions, DNA replication, ribonucleoprotein complex assembly, and metabolic processes.(B) Molecular function analysis highlighted catalytic activity on RNA/DNA, helicase activity, histone binding, and ATP-dependent enzymatic activities.(C) Cellular component analysis demonstrated strong associations with spliceosomal complex, ribonucleoprotein complexes, chromosomal regions, mitochondrial compartments, and precatalytic spliceosomes.** (D)** Functional interaction network of THOC3 with enriched pathways and associated proteins. The chord diagram shows multiple pathway-level connections including chromatin remodeling, cell cycle regulation, DNA replication, homologous recombination, nucleotide excision repair, and spliceosome assembly. Node size reflects gene degree (interaction frequency), while edge color represents functional categories (ATP-dependent chromatin remodeling, base excision repair, cell cycle, DNA replication, mismatch repair, mRNA surveillance, nucleotide transport, nucleotide excision repair, spliceosome).

**Figure 8:**
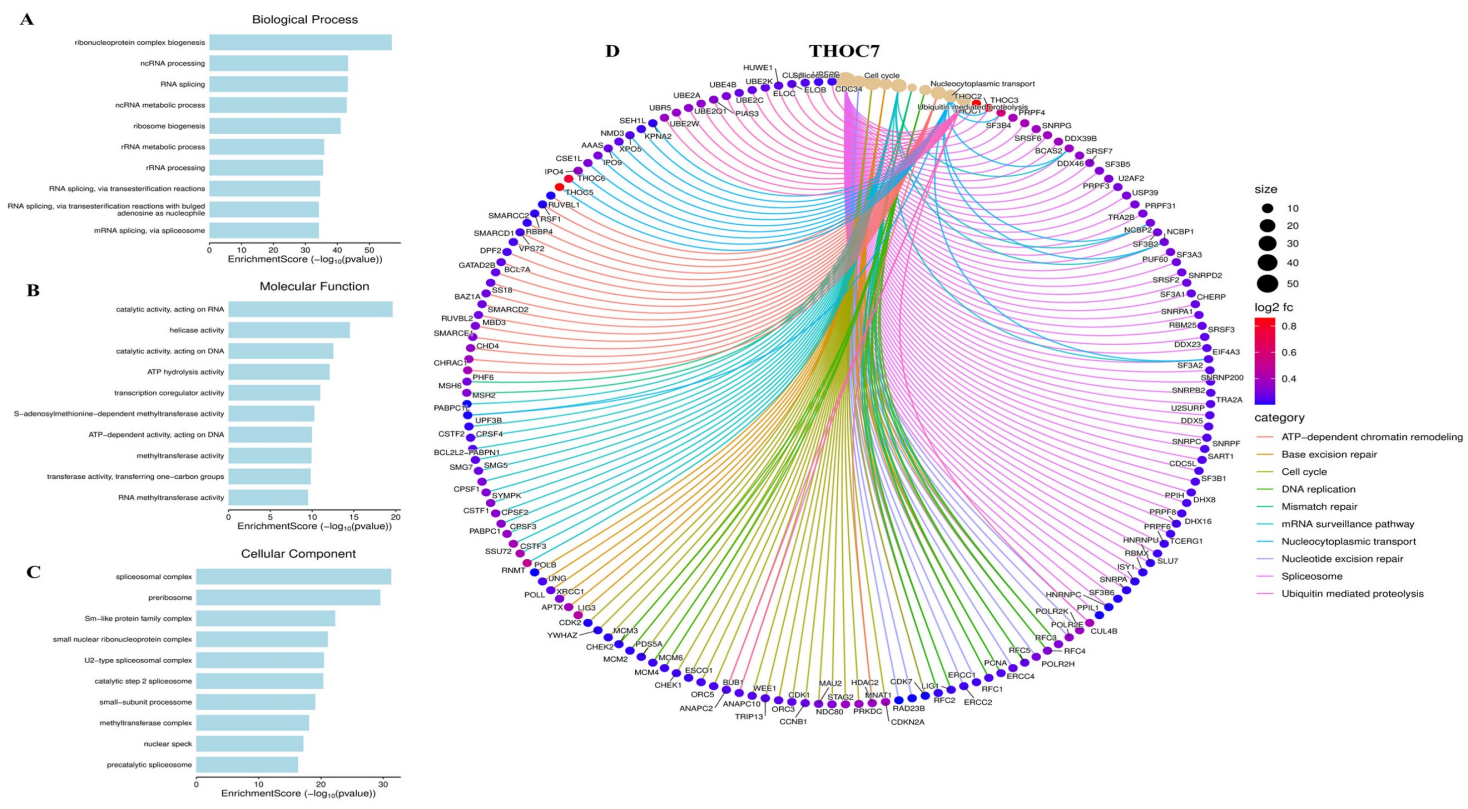
**Gene Ontology (GO) enrichment analysis of THOC7-associated genes. (A-C)** (A) Biological process terms showed strong enrichment in ribonucleoprotein complex biogenesis, ncRNA processing, RNA splicing, ribosome biogenesis, RNA metabolic processes, and transesterification reactions.(B) Molecular function analysis highlighted RNA/DNA catalytic activity, helicase activity, ATP hydrolysis, transcription regulator activity, and RNA methyltransferase activity.(C) Cellular component terms revealed enrichment in spliceosomal complex, peribisome, Sm-like protein family complex, ribonucleoprotein complexes, and precatalytic spliceosome. **(D)** Functional interaction network of THOC7 with enriched pathways and partner genes. The chord diagram illustrates pathway-level associations, including chromatin remodeling, base excision repair, cell cycle regulation, DNA replication, mismatch repair, mRNA surveillance, nucleotide transport, spliceosome function, and ubiquitin-mediated proteolysis. Node size represents interaction frequency (degree), edge colors indicate functional categories, and color intensity of nodes (log2fc) reflects expression levels.

To characterize the biological programs associated with THOC3 and THOC7 expression in LUAD, we performed integrative functional enrichment analyses using Gene Ontology (GO), KEGG, gene set enrichment analysis (GSEA), and MetaCore-based pathway annotation. Consistent with their established roles as components of the THO/TREX complex, genes co-expressed with THOC3 were significantly enriched in RNA metabolism-related processes, including RNA splicing, mRNA surveillance, ribonucleoprotein complex assembly, and nucleocytoplasmic transport (Figure 7A-C), while THOC7-associated genes showed similar enrichment patterns across corresponding GO categories (Figure 8A-C). These results indicate that variation in THOC3 and THOC7 expression is associated with broader transcriptional states characterized by elevated RNA processing capacity. Beyond RNA metabolism, enrichment analyses revealed associations with cell cycle- and genome maintenance-related programs. Specifically, biological process terms linked to DNA replication, mismatch repair, base excision repair, and chromosome organization were enriched among THOC3-associated genes (Figure 7A), whereas THOC7-associated genes showed enrichment in mitotic regulation and chromosome condensation-related processes (Figure 8A). At the molecular function level, enriched categories included nucleic acid binding, ATP-dependent helicase activity, and splicing factor interactions for both THOC3 (Figure 7B) and THOC7 (Figure 8B). Cellular component analysis localized associated genes to spliceosomal complexes, exon junction complexes, and ribonucleoprotein particles (Figures 7C and 8C), reinforcing their association with RNA maturation and quality control machinery.

**Figure 9:**
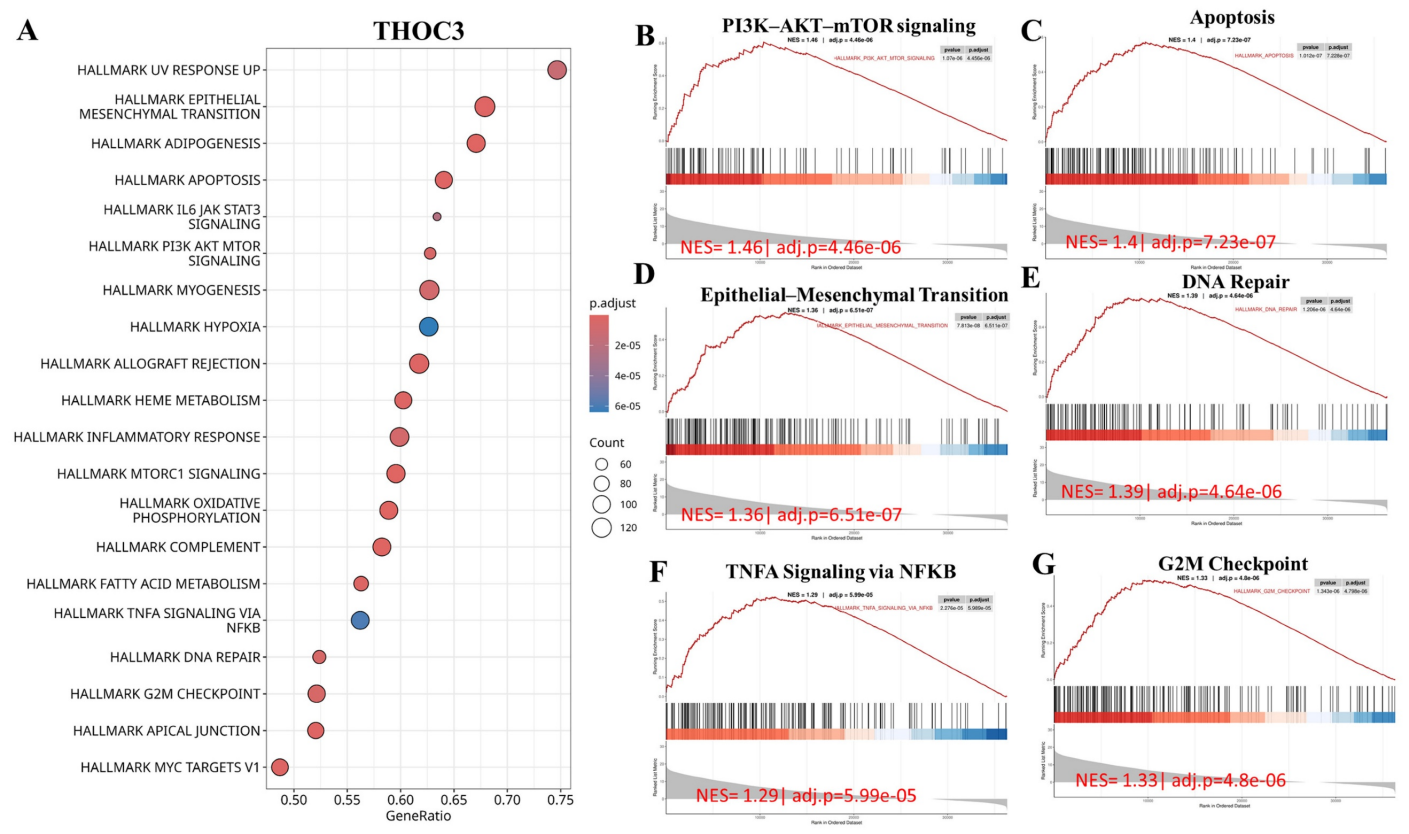
** Gene Set Enrichment Analysis (GSEA) showed that high THOC3 expression in LUAD is linked to several key cancer-related pathways. (A)** The bubble plot summarizes the top enriched Hallmark gene sets, where bubble size represents the number of involved genes and color indicates significance level. The top six enriched pathways were PI3K-AKT-mTOR signaling, apoptosis, epithelial-mesenchymal transition (EMT), DNA repair, G2M checkpoint, and TNFα signaling via NFκB. **(B-G)** Representative enrichment curves show strong activation of these pathways, with high normalized enrichment scores (NES) and low adjusted p-values. Together, these results indicate that THOC3 supports cell growth, survival, DNA repair, and inflammatory signaling.

**Figure 10:**
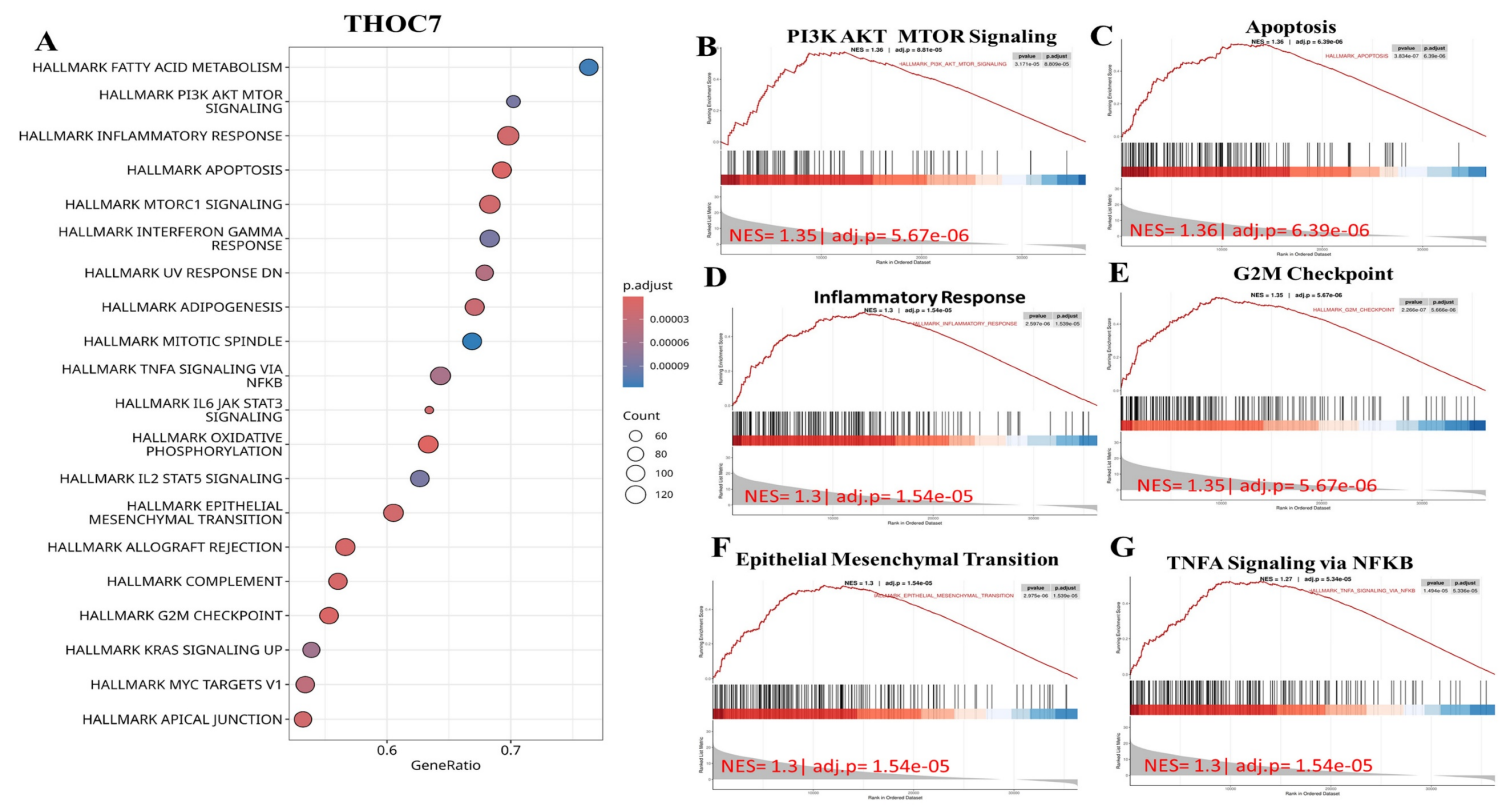
** Gene Set Enrichment Analysis (GSEA) showed that high THOC7 expression in LUAD is associated with several important cancer-related pathways. (A)** The bubble plot summarizes the top enriched Hallmark gene sets, where bubble size represents the number of involved genes and color indicates significance. The top six enriched pathways were PI3K-AKT-mTOR signaling, apoptosis, inflammatory response, G2M checkpoint, epithelial-mesenchymal transition (EMT), and TNFα signaling via NFκB. **(B-G)** Representative enrichment curves show strong activation of these pathways with high normalized enrichment scores (NES) and low adjusted p-values. Together, these results suggest that THOC7 promotes cell proliferation, survival, and inflammatory signaling, supporting its role in LUAD progression and immune modulation.

**Figure 11:**
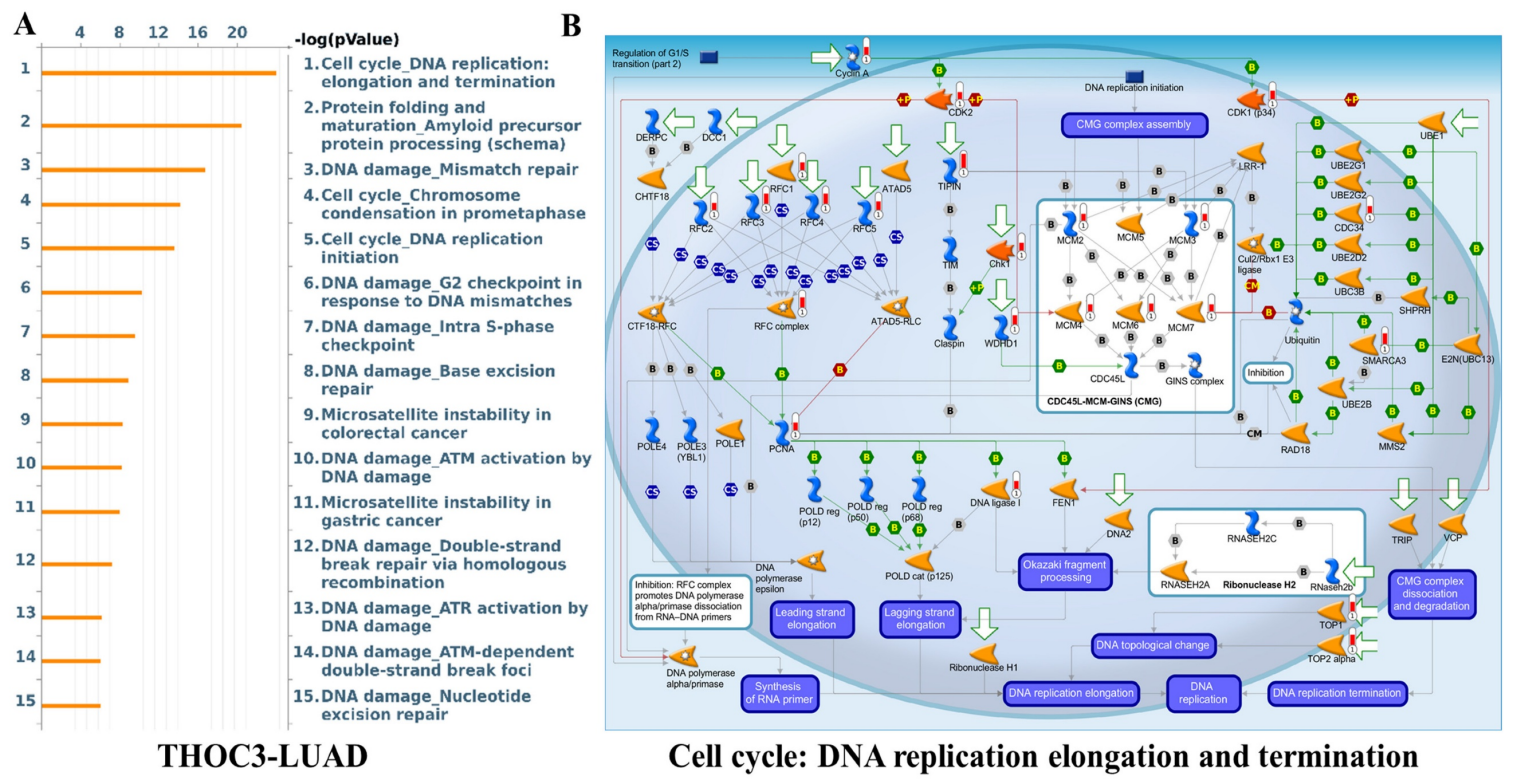
**Pathway enrichment analysis of THOC3 in LUAD. (A)** Bar plot showing the top enriched pathways associated with THOC3 expression in LUAD, based on enrichment scores (-log10 p-value). The most significant pathways included cell cycle: DNA replication elongation and termination, protein folding and precursor maturation, DNA damage-mismatch repair, chromosome condensation in prometaphase, base excision repair, and nucleotide excision repair, highlighting a central role of THOC3 in genome stability and replication fidelity. **(B)** Pathway map of cell cycle: DNA replication elongation and termination, showing THOC3-associated regulatory networks. Core components of replication machinery, including MCM helicase complex, PCNA, DNA polymerases, and checkpoint kinases, are represented, with THOC3 predicted to be integrated within replication and DNA repair signaling modules. Significance: Pathways displayed at p < 0.05 in enrichment analysis.

**Figure 12:**
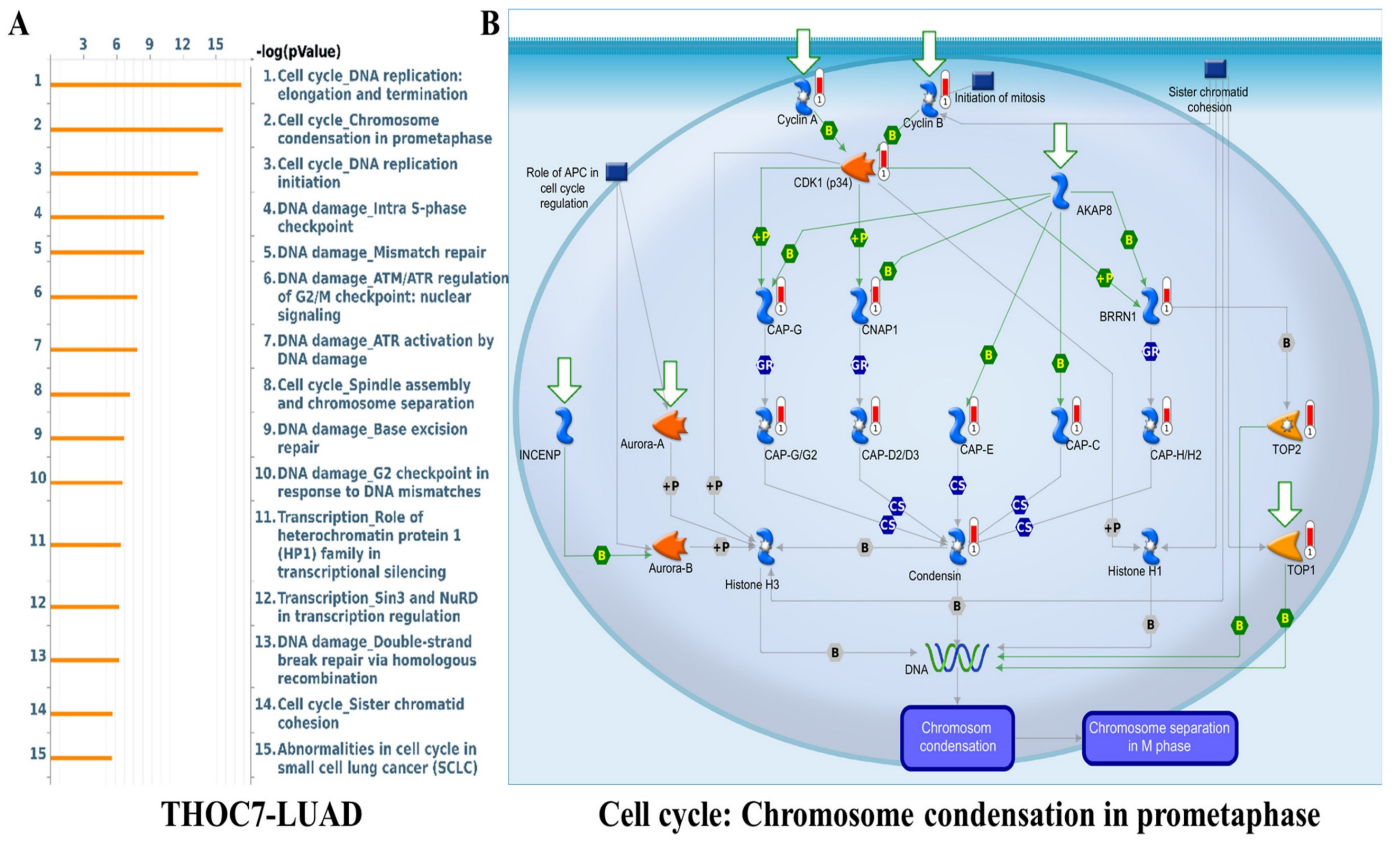
**Pathway enrichment analysis of THOC7 in LUAD. (A)** Bar plot showing the top enriched pathways associated with THOC7 expression in LUAD (-log10 p-value). The most significant pathways included cell cycle: DNA replication elongation and termination, chromosome condensation in prometaphase, DNA replication initiation, intra-S phase checkpoint, mismatch repair, ATR/ATM checkpoint regulation, base excision repair, spindle assembly and chromosome separation, and sister chromatid cohesion, indicating THOC7's strong link to DNA damage response and replication fidelity.** (B)** Pathway map of cell cycle: chromosome condensation in prometaphase, highlighting THOC7-associated molecular interactions. Core regulators such as Cyclin B, Aurora kinases (Aurora-A/B), condensin complex subunits (CAP-D2/D3, CAP-E, CAP-G, CAP-H/H2), histone H3/H1, and topoisomerases (TOP1/2) are shown as central nodes controlling mitotic chromatin condensation and separation. Significance: Pathways displayed at p < 0.05 in enrichment analysis.

To further examine cancer-relevant transcriptional patterns, we performed GSEA using Hallmark gene sets. High THOC3 expression was associated with enrichment of PI3K-AKT-mTOR signaling, apoptosis, epithelial-mesenchymal transition (EMT), DNA repair, TNFα signaling via NF-κB, and G2M checkpoint pathways (Figure 9A-G). Similarly, elevated THOC7 expression was associated with enrichment of PI3K-AKT-mTOR signaling, apoptosis, inflammatory response, EMT, G2M checkpoint, and NF-κB signaling pathways (Figure 10A-G). These enrichments reflect coordinated transcriptional programs commonly observed in aggressive or highly proliferative tumors and represent correlated expression states rather than direct evidence of pathway activation or regulatory control by THOC3 or THOC7. MetaCore KEGG-based pathway analysis provided additional contextual refinement of these associations. For THOC3-associated gene sets, the top enriched pathways included DNA replication elongation and termination, mismatch repair, base excision repair, and intra-S phase checkpoint regulation (Figure 11A-B; Supplementary Tables 1). In contrast, THOC7-associated gene sets showed stronger enrichment in chromosome condensation during prometaphase, mitotic spindle organization, and ATM/ATR-mediated checkpoint signaling (Figure 12A-B; Supplementary Tables 2). These patterns suggest that although THOC3 and THOC7 participate in overlapping RNA processing networks, their expression aligns with partially distinct transcriptional programs related to replication dynamics and mitotic control. Detailed pathway maps illustrating these programs are provided in Supplementary Figures S4-S21. These functional and pathway analyses indicate that elevated THOC3 and THOC7 expression in LUAD is associated with transcriptional programs integrating RNA processing, cell cycle regulation, and genome maintenance (Figures 7-12). However, all enrichment and pathway results presented here are associative and hypothesis-generating. Rather than demonstrating direct regulatory or oncogenic roles, these findings support the interpretation that THOC3 and THOC7 mark transcriptional states linked to high proliferative demand and cellular stress adaptation in LUAD. Further experimental studies will be required to determine whether these associations reflect direct functional involvement or indirect consequences of broader transcriptional reprogramming.

### Immune infiltration and single-cell correlations of THOC3 and THOC7 expression in LUAD

**Figure 13:**
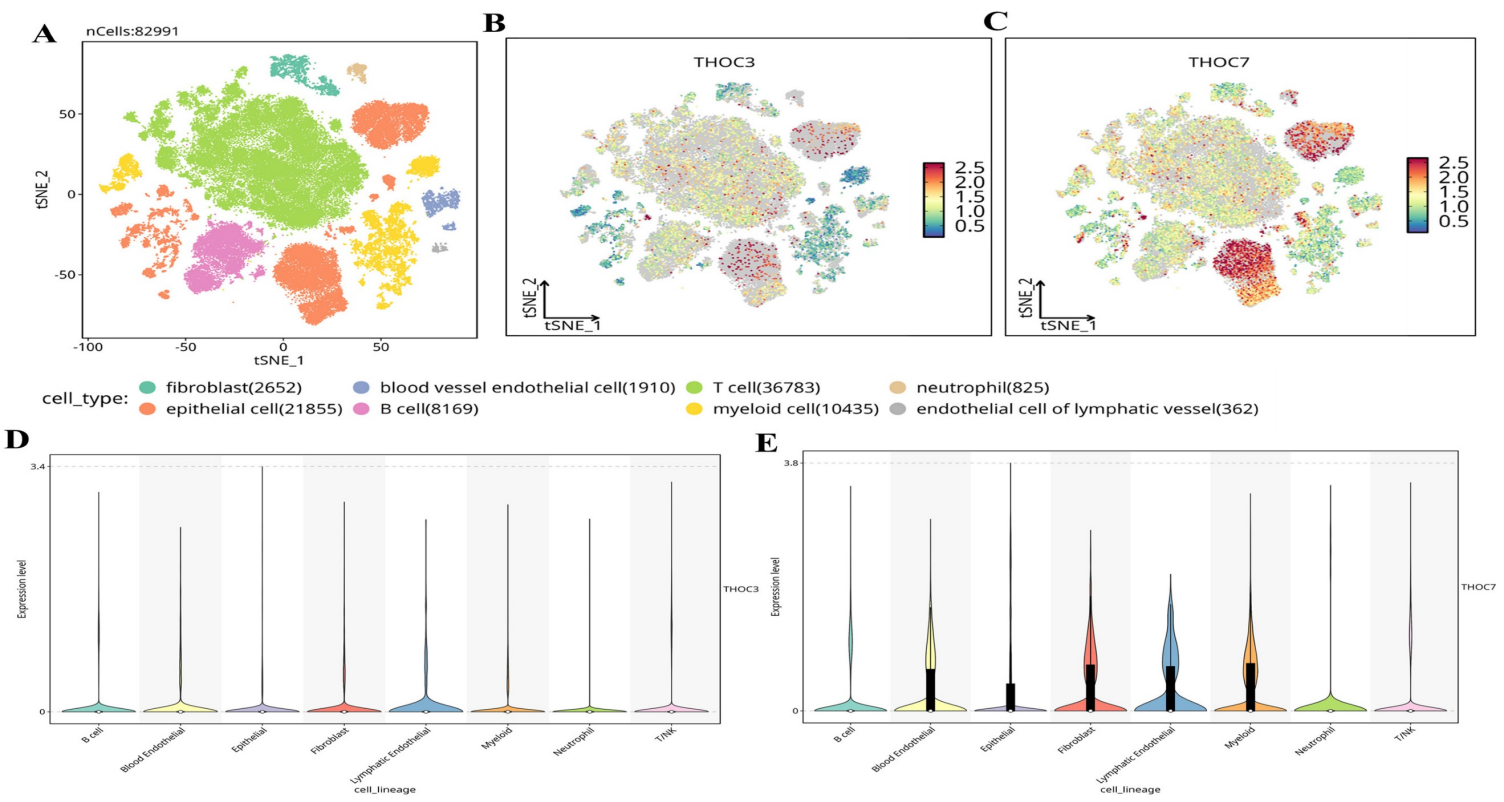
**Single-cell transcriptomic profiling of THOC3 and THOC7 expression in LUAD. (A)** t-SNE plot showing clustering of 82,991 single cells from LUAD into major cell types, including T cells (n = 36,783), epithelial cells (n = 21,855), myeloid cells (n = 10,435), B cells (n = 8,169), fibroblasts (n = 2,652), blood vessel endothelial cells (n = 1,910), neutrophils (n = 825), and lymphatic endothelial cells (n = 362). **(B-C)** Expression distribution of THOC3 and THOC7 across LUAD single-cell populations, visualized by t-SNE. THOC3 showed diffuse but detectable expression across multiple immune and stromal clusters, whereas THOC7 exhibited stronger enrichment in epithelial and myeloid subsets, suggesting both tumor-intrinsic activity and microenvironmental regulation.** (D-E)** Violin plots depicting the expression levels of THOC3 (D) and THOC7 (E) across annotated cell lineages. THOC3 expression was relatively moderate across lineages, whereas THOC7 exhibited higher expression in epithelial cells, fibroblasts, and myeloid cells, consistent with its role in LUAD tumor biology.

To further contextualize the clinical relevance of THOC3 and THOC7 beyond bulk transcriptomic associations, we investigated their relationships with tumor immune infiltration and cellular heterogeneity using independent computational and single-cell approaches. Immune infiltration analysis was performed using the TIMER framework, which adjusts for tumor purity and estimates the abundance of major immune cell populations in TCGA-LUAD samples. As shown in Supplementary Figure 22A-B, elevated THOC3 expression was modestly but significantly negatively correlated with B-cell infiltration (partial cor = -0.113, p = 1.33 × 10⁻²), CD8⁺ T cells (partial cor = -0.156, p = 8.34 × 10⁻⁴), and CD4⁺ T cells (partial cor = -0.124, p = 6.11 × 10⁻³). Similarly, THOC7 expression exhibited inverse correlations with B cells (partial cor = -0.176, p = 9.66 × 10⁻⁵), CD8⁺ T cells (partial cor = -0.107, p = 1.82 × 10⁻²), and CD4⁺ T cells (partial cor = -0.211, p = 2.92 × 10⁻⁶). Although the effect sizes were moderate, these consistent inverse associations suggest that high THOC3/THOC7 expression is linked to a relatively immune-excluded tumor microenvironment. From a biological standpoint, reduced lymphocyte infiltration is frequently associated with impaired anti-tumor immune surveillance and poorer clinical outcomes in LUAD, aligning with the adverse prognostic associations observed for THOC3 and THOC7 in both TCGA and independent GEO cohorts. To validate and refine these findings at single-cell resolution, we analyzed scRNA-seq datasets from LUAD tumors, comprising 82,991 cells across malignant, stromal, and immune compartments (Figure 13A). Major cell populations were resolved, including epithelial tumor cells, fibroblasts, endothelial cells, T cells, B cells, myeloid cells, neutrophils, and lymphatic endothelial cells. Projection of THOC3 and THOC7 expression onto the t-SNE space (Figure 13B-C) revealed heterogeneous but preferential enrichment within malignant epithelial clusters, with additional expression observed in myeloid and neutrophil populations. Quantitative violin plots (Figure 13D-E) confirmed that epithelial and myeloid lineages exhibited the highest expression levels, whereas lymphoid populations showed comparatively low expression.

**Figure 14:**
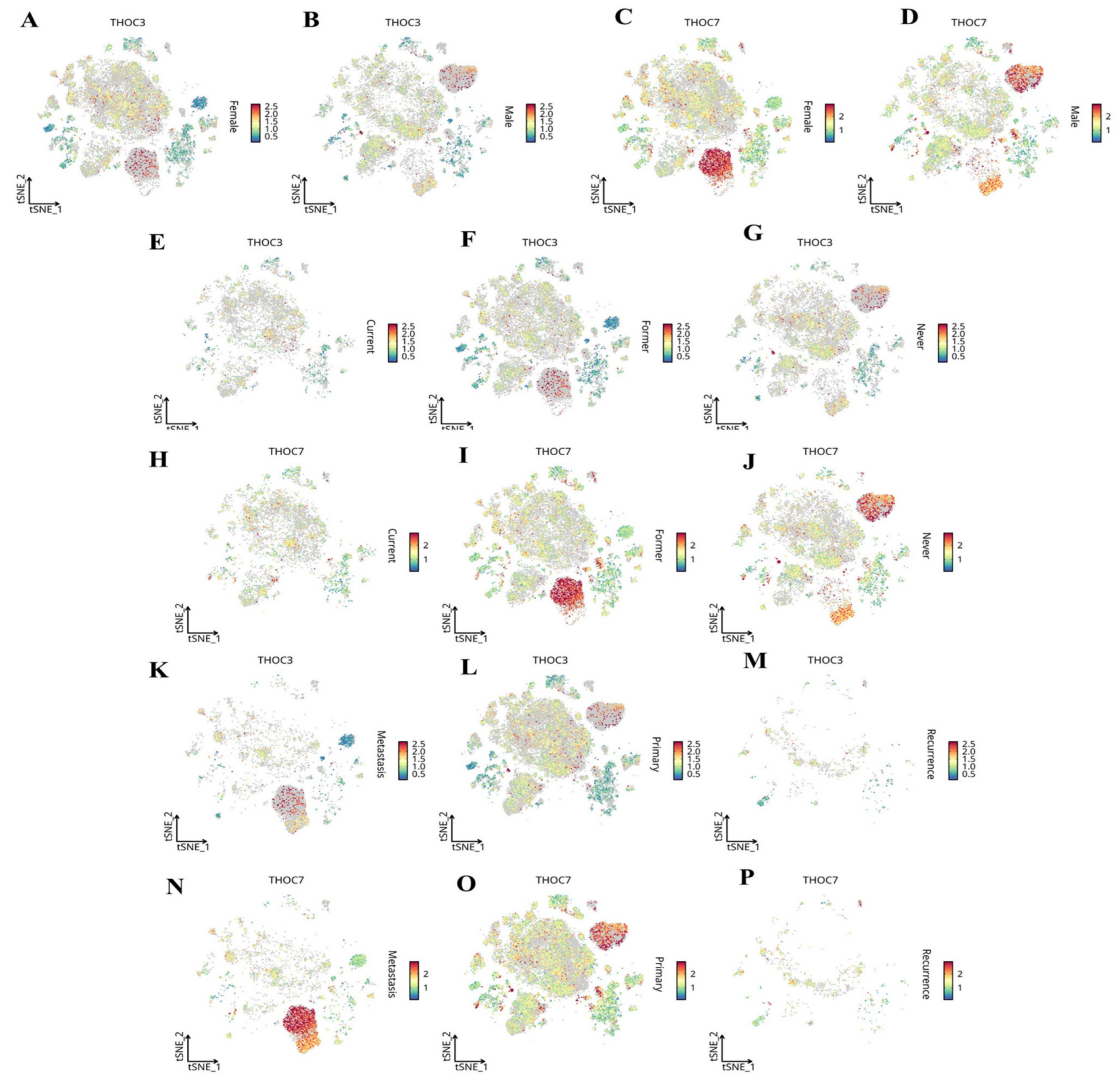
**Single-cell transcriptomic distribution of THOC3 and THOC7 in LUAD across clinical subgroups. (A-B)** Expression of THOC3 across LUAD single-cell populations stratified by primary tumors and lymph node metastasis (N stage).** (C-D)** Expression of THOC7 in the same subgroups, showing stronger enrichment in malignant epithelial and myeloid subsets. **(E-G)** Expression of THOC3 in LUAD patients stratified by clinical stage (C), primary tumor site (F), and nodal metastasis (G). **(H-J)** Expression of THOC7 in LUAD across clinical stage (H), primary tumor site (I), and nodal metastasis (J). **(K-M)** Expression of THOC3 in LUAD samples grouped by metastasis (K), primary lesions (L), and recurrence (M).** (N-P)** Expression of THOC7 across the same categories (metastasis, primary lesions, and recurrence). Heatmap scale (blue to red) indicates low-to-high gene expression levels (log2 TPM).

**Figure 15:**
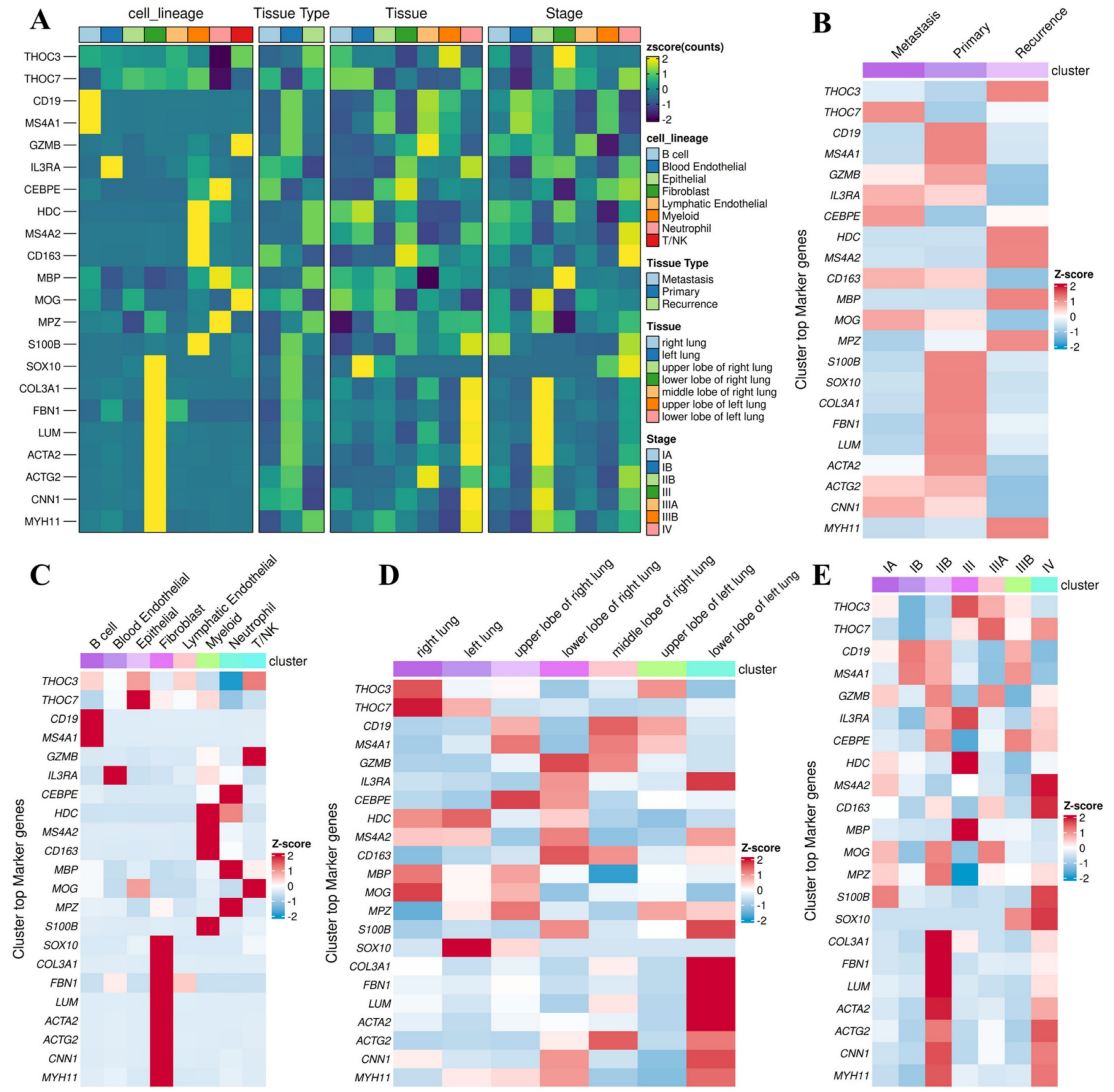
** Stratified single-cell and tissue-level profiling of THOC3 and THOC7 across LUAD subtypes and clinical contexts. (A)** Heatmap displaying normalized expression (Z-score) of THOC3, THOC7, and representative lineage or stromal markers across major cell lineages, tissue types (primary, metastatic, recurrent), anatomical locations, and pathological stages of LUAD. Both THOC3 and THOC7 show enrichment in malignant epithelial clusters and recurrence-associated tissues, aligning with proliferative and repair-linked profiles.** (B)** Cluster-wise co-expression heatmap summarizing top marker genes for primary, metastatic, and recurrent LUAD subsets of THOC3/THOC7. Elevated THOC7 expression is particularly evident in recurrence clusters, suggesting a link to proliferative resilience and post-therapy adaptation.** (C)** Lineage-resolved Z-score distribution of THOC3/THOC7 across immune, stromal, and epithelial cell populations, highlighting preferential enrichment within epithelial and fibroblast compartments. **(D)** Spatial distribution of THOC3/THOC7 across lung lobes and anatomical sites, showing conserved activation in upper and middle lobes, typical of LUAD lesion distribution. **(E)** Stage-wise analysis (I-IV) demonstrating progressive upregulation of THOC3 and THOC7 with advancing tumor stage, supporting their role in disease progression and recurrence.

We next examined context-dependent expression patterns across clinically relevant subgroups using stratified single-cell analyses (Figure 14). Both THOC3 and THOC7 were consistently enriched in malignant epithelial cells relative to normal epithelial counterparts (Figure 14A-D), supporting a tumor-intrinsic transcriptional upregulation. Stratification by smoking status revealed higher expression in smoking-associated LUAD clusters (Figure 14E-H), suggesting that carcinogen-induced replication and transcriptional stress may select for enhanced RNA export capacity. Notably, both genes were further upregulated in TP53-mutant tumors (Figure 14I-J), a context characterized by compromised checkpoint control and increased reliance on alternative stress-adaptation mechanisms. Importantly, expression of THOC3 and THOC7 persisted in recurrent tumors (Figure 14K-P), with THOC7 showing particularly strong enrichment in proliferative epithelial subsets, indicating that THOC-mediated RNA processing is maintained during disease progression and relapse rather than being restricted to early tumorigenesis. Integrated tissue- and lineage-level analyses further supported these observations. Heatmap-based profiling across LUAD subtypes and pathological stages (Figure 15A-E) demonstrated progressive upregulation of both THOC3 and THOC7 from early-stage to advanced and recurrent tumors. Co-expression with stromal and extracellular matrix-associated genes, including ACTA2, COL3A1, CNN1, and FBN1, suggests that THOC-driven RNA export may also contribute indirectly to stromal remodeling and mechanical adaptation within the tumor microenvironment. Spatial analyses indicated preferential enrichment in upper and middle lung lobes, anatomical regions frequently affected by LUAD, potentially reflecting adaptation to hypoxic and high-stress niches. Collectively, immune deconvolution and single-cell analyses provide orthogonal validation that complements bulk transcriptomic findings from TCGA and GEO cohorts. While THOC3 and THOC7 are most prominently expressed in malignant epithelial cells, their detectable presence in myeloid and neutrophil populations suggests broader involvement in shaping tumor-microenvironment interactions. Importantly, these analyses remain descriptive and associative; they do not imply direct immunomodulatory or causal functions. Rather, the data supports a model in which elevated THOC3 and THOC7 expression reflects transcriptional states associated with high proliferative demand, replication stress tolerance, and immune exclusion, features that collectively characterize aggressive LUAD phenotypes.

## Discussion

Despite substantial advances in early detection, molecular stratification, and targeted and immune-based therapies, lung adenocarcinoma (LUAD) continues to exhibit high rates of relapse and treatment resistance, resulting in poor long-term survival for many patients 80, 81. Increasing evidence indicates that post-transcriptional gene regulation, particularly RNA processing and nuclear export, is actively remodeled in cancer to sustain oncogenic signaling and preserve genome stability under stress. However, the contribution of individual components of the transcription-export (TREX) machinery to LUAD pathogenesis has remained incompletely characterized 19. While select THOC family members, such as THOC1 and THOC5, have been implicated in other malignancies, the biological and clinical relevance of THOC3 and THOC7 in LUAD has not been systematically explored.

In this study, we address this gap through an integrative, multi-layered analysis and identify THOC3 and THOC7 as consistently dysregulated TREX components associated with LUAD progression. Pan-cancer and LUAD-specific transcriptomic analyses demonstrated that THOC3 and THOC7 are preferentially upregulated in LUAD compared with normal lung tissues, whereas other THOC subunits showed weaker or inconsistent alterations. Importantly, these observations were not restricted to TCGA data alone. Independent validation using multiple GEO LUAD cohorts, including GSE13213 and GSE31210, confirmed significantly elevated expression of THOC3 and THOC7 in tumor tissues relative to normal lung samples and reproduced their adverse survival associations. The consistency of these findings across distinct patient populations, profiling platforms, and normalization strategies strengthens the robustness of our conclusions and reduces the likelihood that the observed associations are dataset-specific or driven by cohort bias. At the protein level, immunohistochemical data from the Human Protein Atlas confirmed increased expression of THOC3 and THOC7 in LUAD tumors, with distinct subcellular distributions. THOC3 predominantly localized to cytoplasmic and membranous compartments, whereas THOC7 showed strong nuclear enrichment 82. The observed compartmentalization of THOC3 and THOC7 is consistent with a functional division within the TREX complex. THOC3 may preferentially support cytoplasmic engagement of exported transcripts, facilitating efficient translation of proliferation-associated mRNAs, whereas THOC7 likely contributes to nuclear RNA surveillance and export checkpoint regulation. The presence of these patterns in an EGFR-active epithelial context supports a model in which growth factor signaling enhances RNA export throughput to meet increased transcriptional demand, a common feature of aggressive LUAD.

Meanwhile, our analysis suggests that LUAD cells may exploit THOC3 and THOC7 through complementary regulatory layers. DNA methylation profiling revealed tumor-specific hypomethylation at CpG sites associated with both genes, providing a plausible epigenetic basis for their transcriptional activation. In parallel, protein-protein interaction network analysis positioned THOC3 and THOC7 as central hubs within the TREX machinery, interacting with canonical RNA-processing factors such as DDX39B, ALYREF, CHTOP, and MAGOH, as well as nuclear pore-associated proteins including RAE1 and NUP88. These interaction patterns indicate that THOC3 and THOC7 participate in coordinated RNA maturation, surveillance, and export rather than acting as passive structural components 83. Functional enrichment analyses further supports this interpretation. Gene Ontology and KEGG analyses linked both genes to RNA splicing, mRNA surveillance, nucleocytoplasmic transport, and DNA replication and repair pathways. Gene set enrichment analysis associated high THOC3 and THOC7 expression with hallmark oncogenic programs, including PI3K-AKT-mTOR signaling, epithelial-mesenchymal transition, apoptosis-related pathways, and inflammatory response. MetaCore pathway analysis refined these observations, indicating preferential associations of THOC3 with DNA replication elongation and repair pathways, while THOC7 showed stronger links to chromosomal condensation, mitotic regulation, and checkpoint signaling. Together, these results suggest that THOC3 and THOC7 may support LUAD cell survival by stabilizing transcriptional programs required for replication stress tolerance and genome maintenance. Importantly, immune deconvolution and single-cell transcriptomic analyses extended these findings beyond tumor-intrinsic effects. Bulk tumor immune profiling revealed modest but consistent inverse correlations between THOC3/7 expression and lymphocyte infiltration, suggesting an association with immune-excluded tumor states. Single-cell RNA sequencing refined this view, demonstrating that THOC3 and THOC7 are most prominently expressed in malignant epithelial clusters, with additional expression in myeloid and neutrophil populations. Stratified analyses further showed enrichment in smoking-associated, TP53-mutant, and recurrent LUAD subsets, contexts characterized by elevated genomic instability and therapeutic stress 84. While these observations remain associative, they indicate that THOC3 and THOC7 expression reflects transcriptional states linked to aggressive disease behavior and adaptive resilience.

## Conclusion

**Figure 16:**
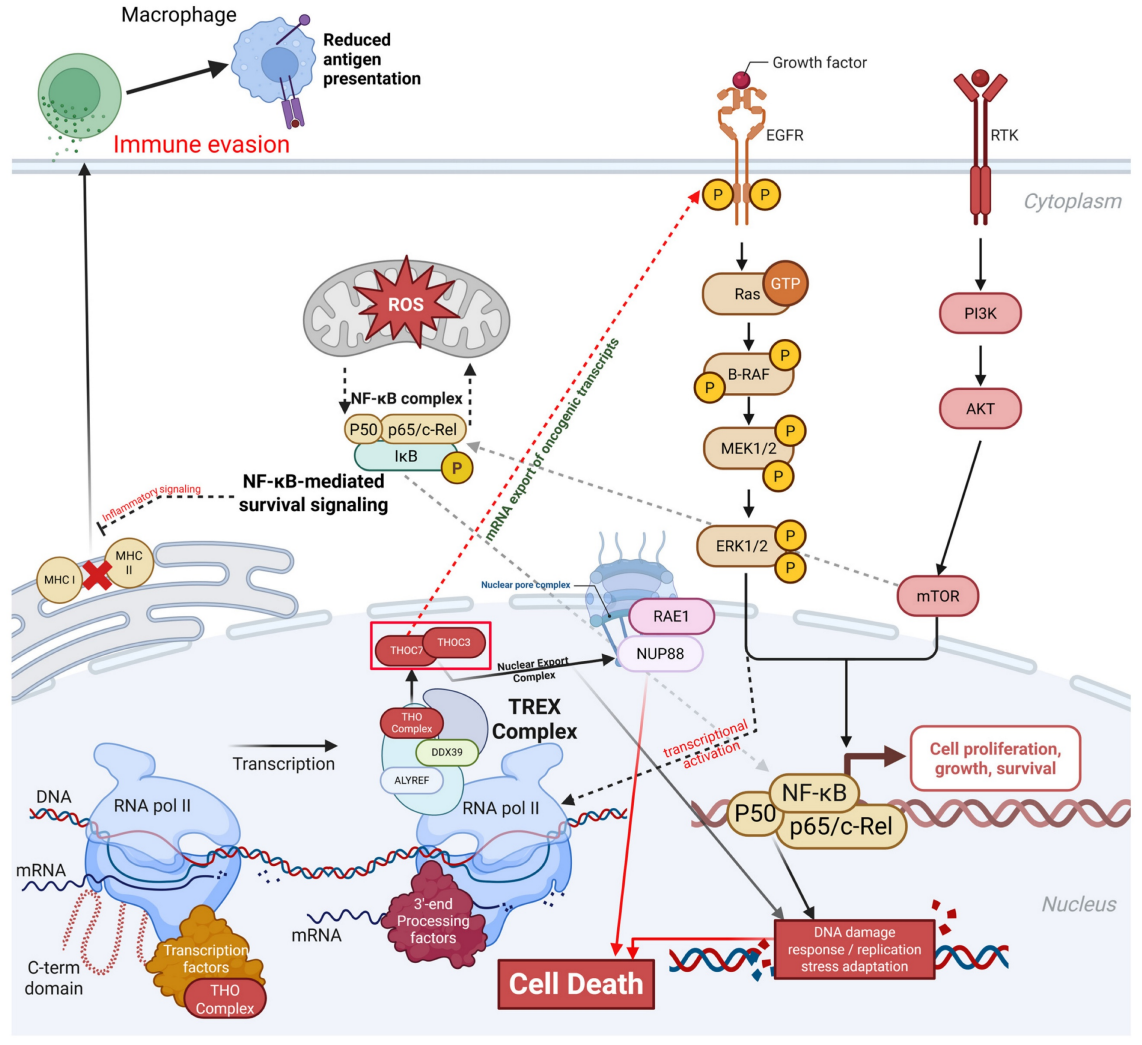
** THOC3/THOC7-mediated TREX activation integrates mRNA export, oncogenic signaling, stress adaptation, and immune evasion in LUAD.** Schematic summary presenting a hypothesis generating framework in which elevated THOC3 and THOC7, identified as reproducibly upregulated and associated with adverse clinical outcomes across multiple LUAD cohorts, co vary with transcriptomic programs related to RNA processing and nuclear export, cell cycle progression, genome maintenance, and stress or inflammatory signaling. The model integrates established roles of the THO/TREX complex in mRNP maturation and nucleocytoplasmic export from prior literature with association-based signals from bulk transcriptomics, epigenetic profiling, co expression network analyses, immune estimation results, and single cell context mapping in this study.

These integrative analyses identify THOC3 and THOC7 as consistently upregulated RNA-export-associated genes in lung adenocarcinoma across TCGA and independent GEO cohorts, with reproducible associations with poor overall and disease-free survival. Epigenetic profiling and protein interaction analyses place both genes as central components of the TREX complex, linking RNA processing and nuclear export with DNA replication, repair, and checkpoint-related transcriptional programs. Functional enrichment, immune deconvolution, and single-cell transcriptomic analyses further indicate that elevated THOC3 and THOC7 expression marks LUAD states characterized by high transcriptional demand, replication stress adaptation, and altered tumor-microenvironment interactions. These convergent multi-omics findings support THOC3 and THOC7 as robust biomarkers of LUAD aggressiveness and provide a rationale for future functional studies to evaluate RNA export dependency as a potential therapeutic vulnerability (Figure 16).

## Supplementary Material

Supplementary figures and tables.

## Figures and Tables

**Table 1:** Basic characteristics of THOC Complex genes.

Gene symbol	Official Full Name	HGNC ID	Gene ID	Aliases	Description	Location on chromosome
THOC1	THO complex subunit 1	19070	9984	P84; HPR1; P84N5; DFNA86	THOC1 (THO Complex Subunit 1) is a Protein Coding gene. Diseases associated with THOC1 include Deafness, Autosomal Dominant 86 and Autosomal Dominant Nonsyndromic Deafness. Among its related pathways are Transport of Mature Transcript to Cytoplasm and Gene expression (Transcription).	18p11.32
THOC2	THO complex subunit 2	19073	57187	AMC7; THO2; MRX12; MRX35; CXorf3; XLID12; hTREX120; dJ506G2.1	THOC2 (THO Complex Subunit 2) is a Protein Coding gene. Diseases associated with THOC2 include Intellectual Developmental Disorder, X-Linked, Syndromic, Kumar Type and X-Linked Intellectual Disability-Short Stature-Overweight Syndrome. Among its related pathways are Transport of Mature Transcript to Cytoplasm and Gene expression (Transcription).	Xq25
THOC3	THO complex subunit 3	19072	84321	THO3; hTREX45	THOC3 (THO Complex Subunit 3) is a Protein Coding gene. Diseases associated with THOC3 include Lethal Congenital Contracture Syndrome 1 and Sarcoma. Among its related pathways are Transport of Mature Transcript to Cytoplasm and Gene expression (Transcription).	5q35.2
THOC5	THO Complex Subunit 5	19074	8563	Fmip; PK1.3; fSAP79; C22orf19	THOC5 (THO Complex Subunit 5) is a Protein Coding gene. Diseases associated with THOC5 include Meningioma and Sarcoma. Among its related pathways are Transport of Mature Transcript to Cytoplasm and Gene expression (Transcription).	22q12.2
THOC6	THO complex subunit 6	79228	28369	WDR58; fSAP35; MMRFCGU	THOC6 (THO Complex Subunit 6) is a Protein Coding gene. Diseases associated with THOC6 include Beaulieu-Boycott-Innes Syndrome and Chromosome 16P13.3 Duplication Syndrome. Among its related pathways are Transport of Mature Transcript to Cytoplasm and Gene expression (Transcription).	16p13.3
THOC7	THO complex subunit 7	80145	29874	fSAP24; hTREX30; NIF3L1BP1	THOC7 (THO Complex Subunit 7) is a Protein Coding gene. Diseases associated with THOC7 include Ogden Syndrome and Microphthalmia, Syndromic 1. Among its related pathways are Transport of Mature Transcript to Cytoplasm and Gene expression (Transcription).	3p14.1

## Abbreviations

LUAD: Lung adenocarcinoma
NSCLC: Non-small cell lung cancer
SCLC: Small-cell lung cancer
OS: Overall survival
DFS: Disease-free survival
TREX: Transcription-export complex
THOC: THO complex subunit
PPI: Protein-protein interaction
IHC: Immunohistochemistry
scRNA-seq: Single-cell RNA sequencing
GO: Gene Ontology
KEGG: Kyoto Encyclopedia of Genes and Genomes
GSEA: Gene Set Enrichment Analysis
HPA: Human Protein Atlas
LDCT: Low-dose computed tomography
TCGA: The Cancer Genome Atlas
GTEx: Genotype-Tissue Expression project
UALCAN: University of Alabama at Birmingham Cancer Data Analysis Portal
EGFR: Epidermal growth factor receptor
PCNA: Proliferating cell nuclear antigen
MCM: Minichromosome maintenance proteins
CDK: Cyclin-dependent kinase
ATM: Ataxia telangiectasia mutated
ATR: Ataxia telangiectasia and Rad3-related
EMT: Epithelial-mesenchymal transition
