# Supplementary material for: Integrative Multi-Omics and Single-Cell Analysis Reveal THOC3 and THOC7 as Oncogenic RNA Processing Regulators in Lung Adenocarcinoma
Source: Int J Med Sci. 2026 Mar 9;23(4):1408–30. doi: 10.7150/ijms.128975 (PMC13048885; doi:10.7150/ijms.128975)
Supplement: Supplementary file 1 — Supplementary figures and tables. [file ijmsv23p1408s1.pdf]

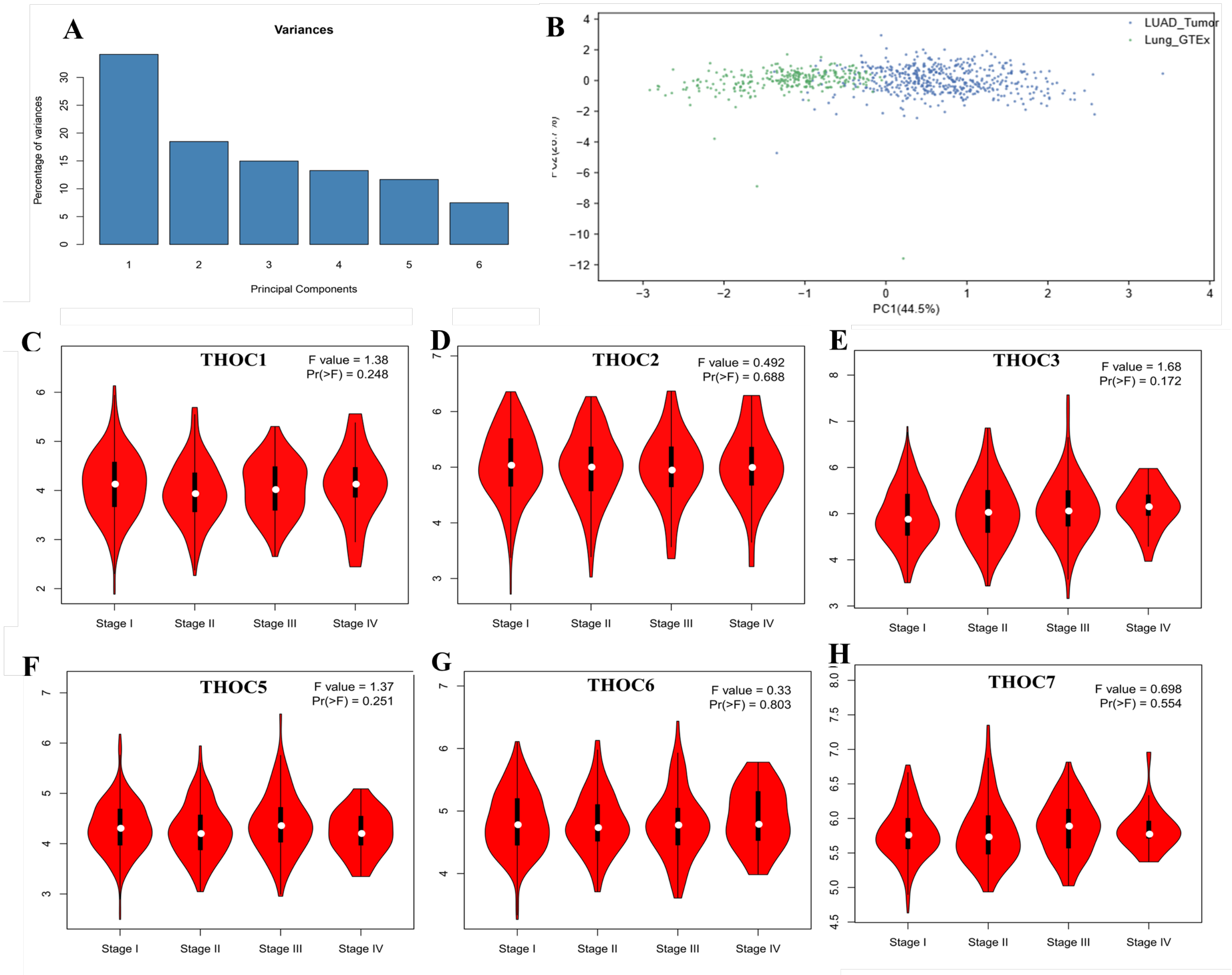

Supplementary Figure 1: Expression of THOC family genes across LUAD clinical stages and assessment of cohort-driven batch effects. (A) Percentage of variance explained by the top six principal components derived from principal component analysis (PCA) of combined TCGA-LUAD tumor and GTEx normal lung transcriptomic datasets, illustrating the relative contribution of each component to overall variance. **(B)** PCA plot (PC1 vs PC2) of combined TCGA-LUAD tumor samples and GTEx normal lung tissues prior to batch correction. Samples are colored by cohort, revealing clear cohort-driven separation, indicative of batch effects between TCGA and GTEx datasets. **(C–H)** Violin plots showing mRNA expression levels of THOC1 (A), THOC2 (B), THOC3 (C), THOC5 (D), THOC6 (E), and THOC7 (F) across pathological stages I–IV in lung adenocarcinoma (LUAD) patients from the TCGA cohort. Each violin represents the distribution of expression values within a given clinical stage. White dots indicate median expression levels, and black vertical bars denote the interquartile range. One-way ANOVA was used to evaluate differences in expression across stages; F statistics and corresponding p-values are shown in each panel. No statistically significant stage-dependent differences were observed for THOC family members, although THOC3 displayed a modest, non-significant trend toward higher expression in advanced stages.

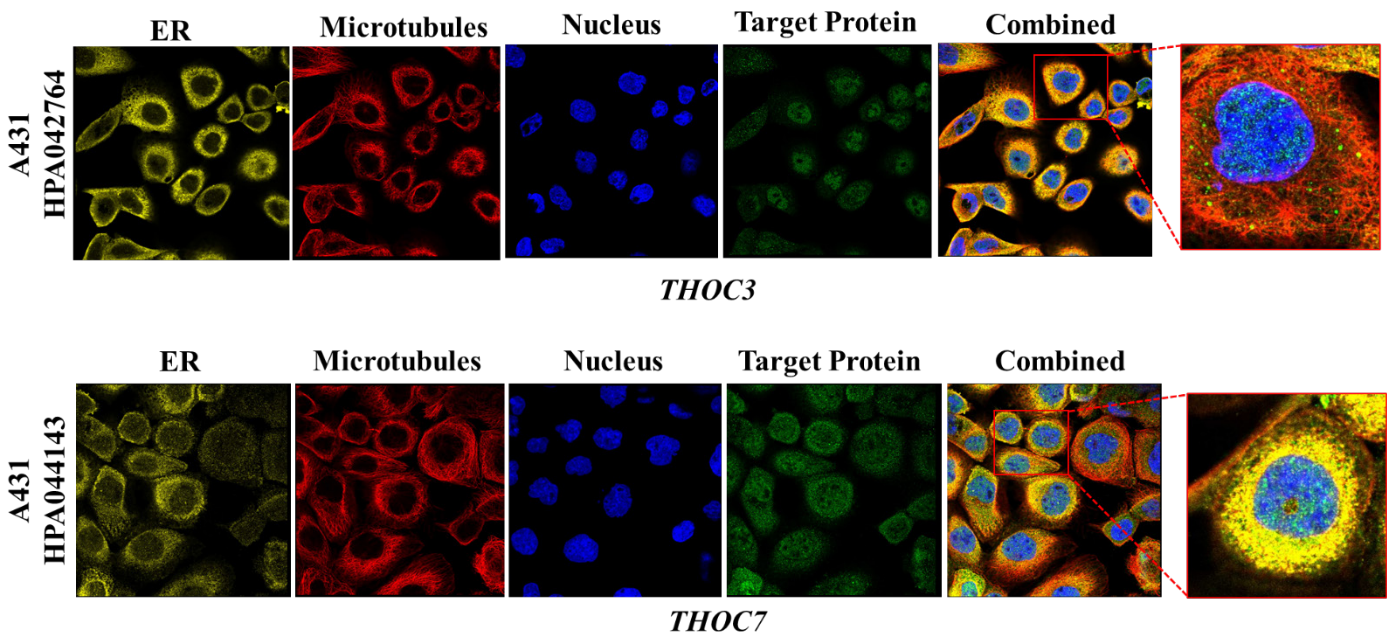

Supplementary Figure 2. Immunofluorescence validation of THOC3 and THOC7 subcellular localization in an EGFR-high epithelial model. Immunofluorescence staining was used to assess the subcellular localization of THOC3 and THOC7. THOC3 showed predominantly cytoplasmic and membranous distribution, whereas THOC7 was mainly localized to the nucleus, consistent with immunohistochemical findings in LUAD tissues. A431 cells were employed as an epithelial reference model due to their high EGFR expression, a key molecular feature of LUAD, and their well-defined cellular morphology, enabling clear visualization of EGFR-associated nucleocytoplasmic compartmentalization of THOC3 and THOC7.

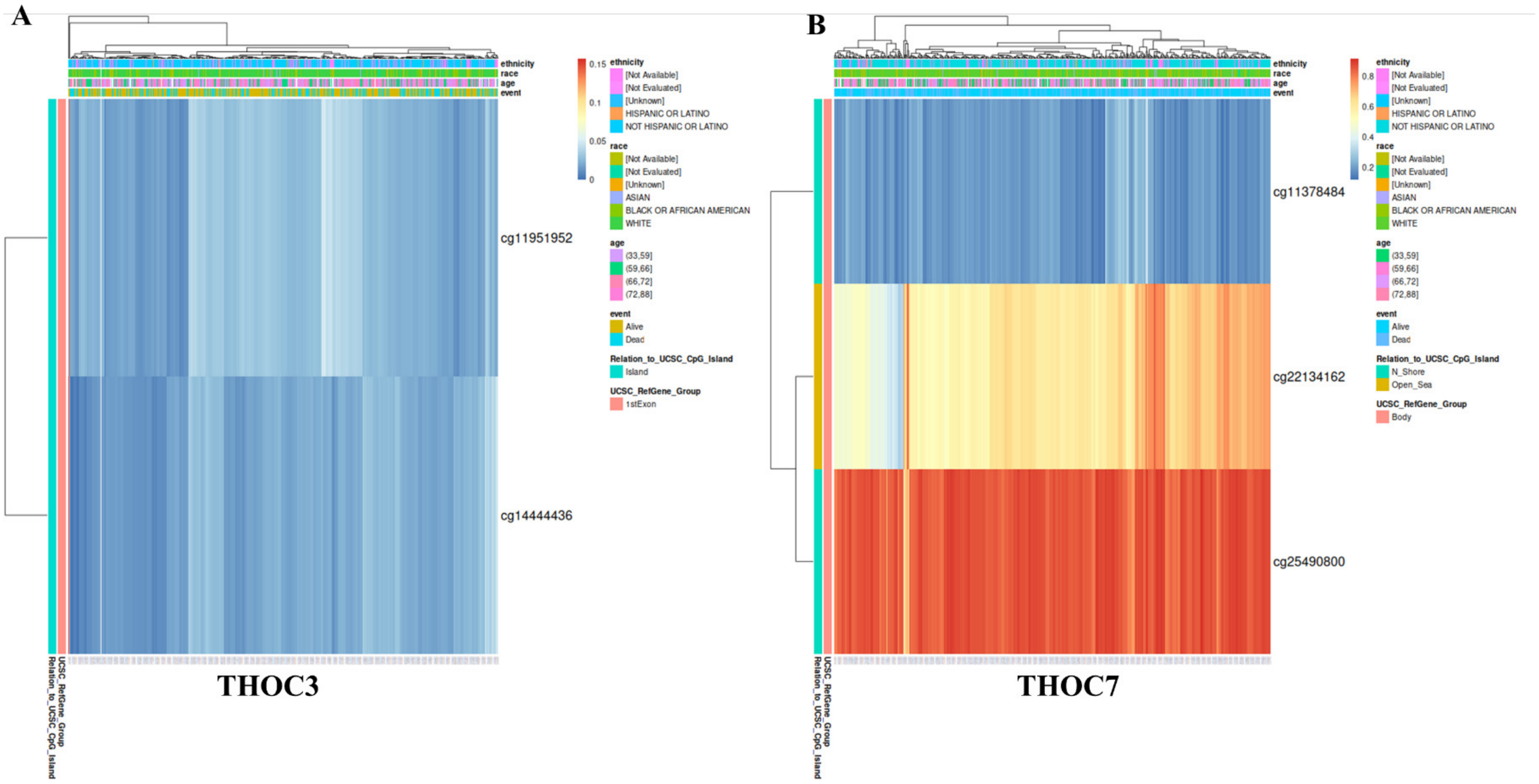

Supplementary Figure 3. Differential DNA methylation patterns of THOC3 and THOC7 in LUAD.(A) Heatmap showing methylation status of CpG sites associated with THOC3 (cg11951952 and cg1444436) across LUAD patient samples from TCGA. Predominantly hypomethylated regions are observed, suggesting epigenetic activation.**(B)** Heatmap showing CpG methylation levels for THOC7 (cg11378484, cg22134162, cg25490800). THOC7 exhibits broad hypomethylation across multiple CpG islands and gene body regions. Annotations for patient ethnicity, race, age, and survival status are displayed at the top of each heatmap.

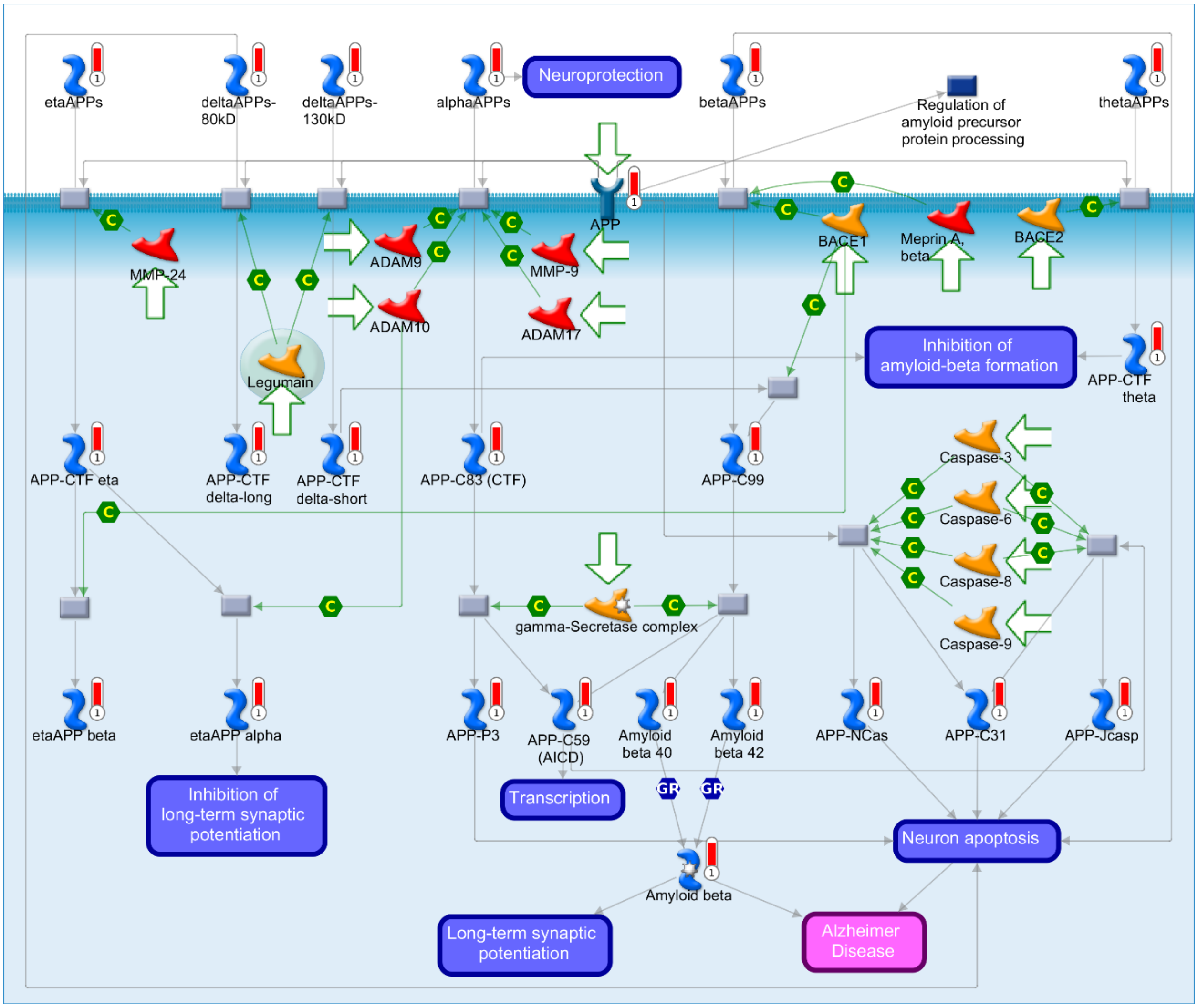

Supplementary Figure 4. Enrichment of the pathway Protein folding and maturation: amyloid precursor protein processing (schema) in LUAD for THOC3.

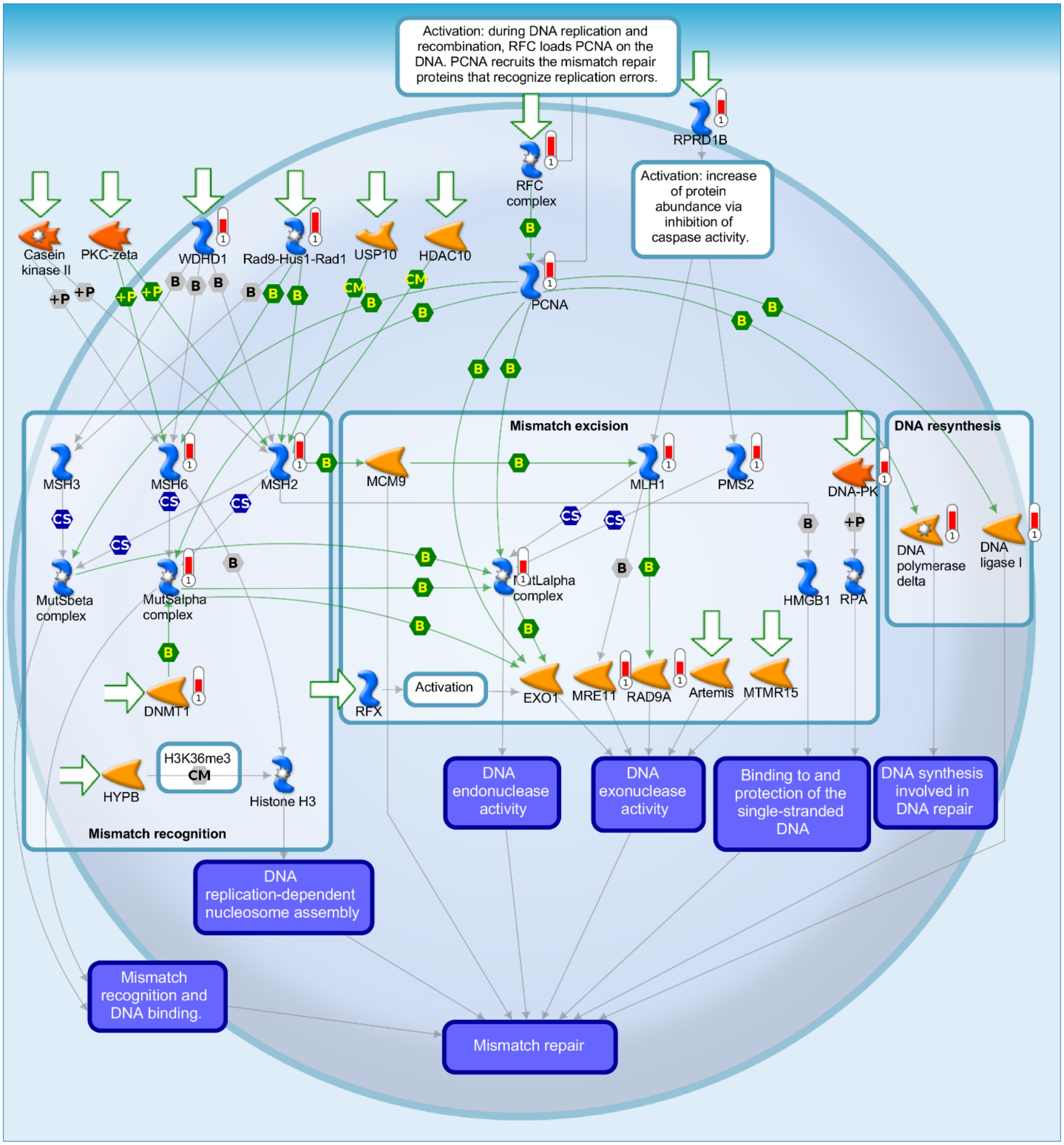

Supplementary Figure 5. Enrichment of the pathway DNA damage: mismatch repair in LUAD for THOC3.

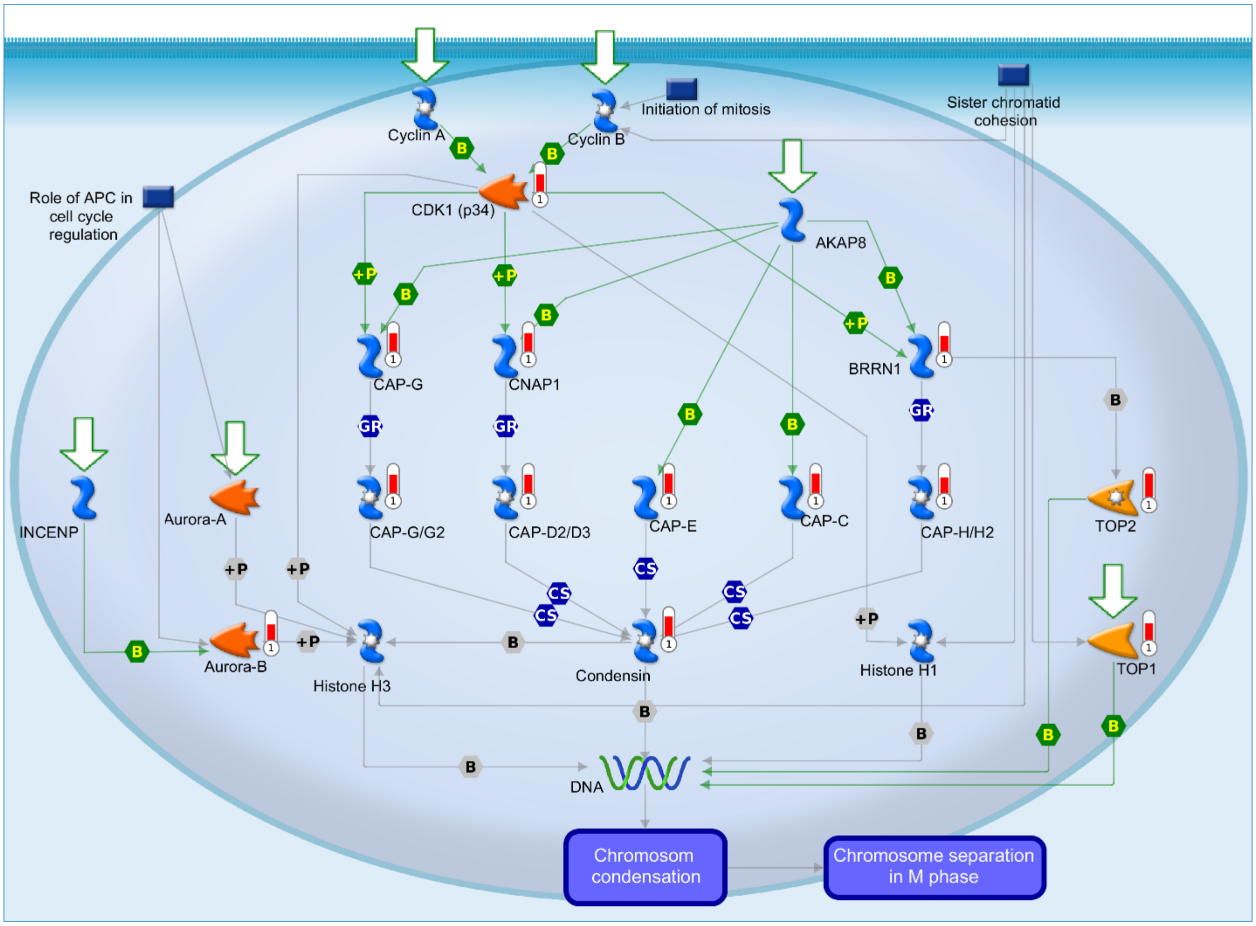

Supplementary Figure 6. Enrichment of the pathway Cell cycle: chromosome condensation in prometaphase in LUAD for THOC3.

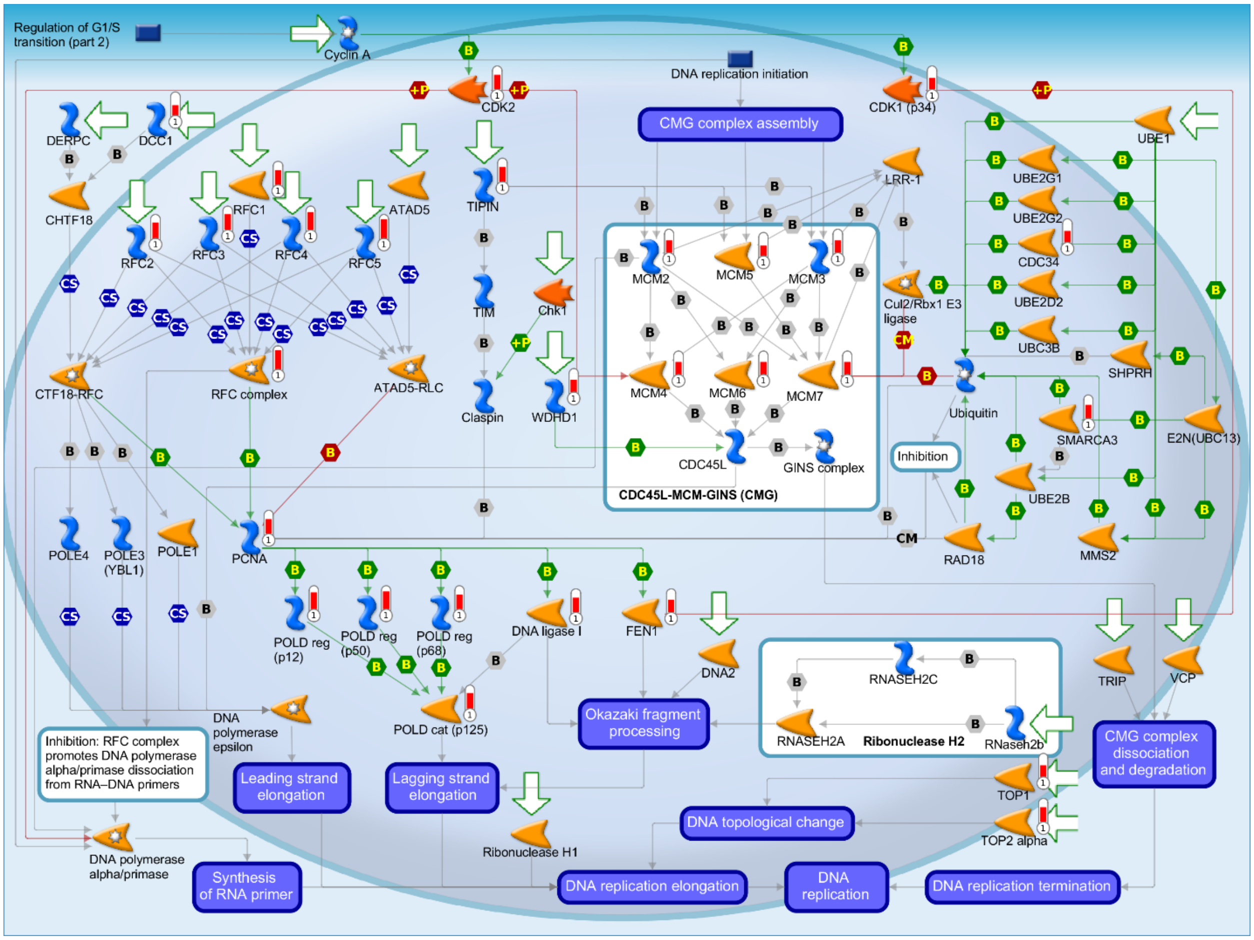

Supplementary Figure 7. Enrichment of the pathway Cell cycle: DNA replication initiation in LUAD for THOC3.

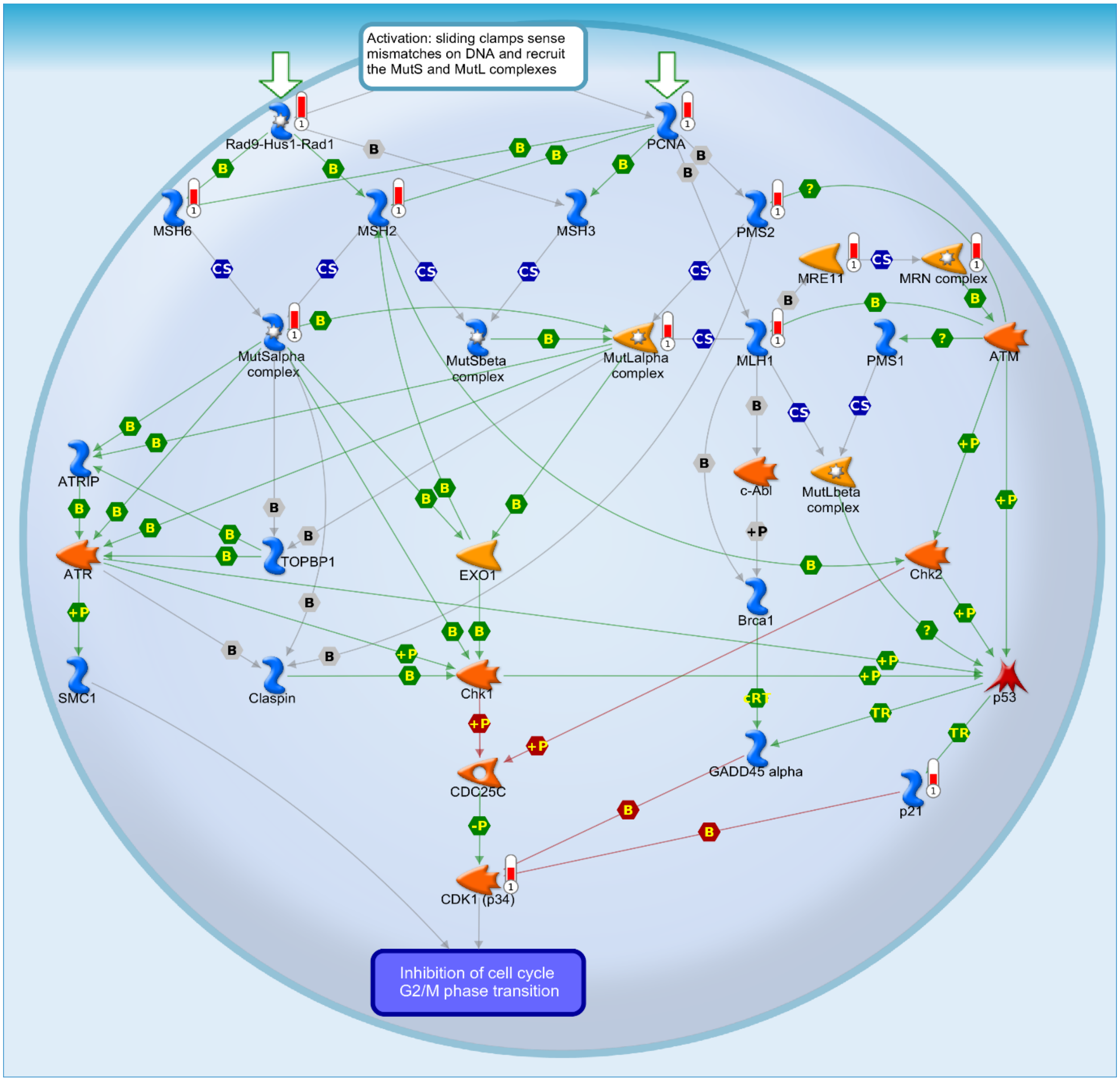

Supplementary Figure 8. Enrichment of the pathway DNA damage: G2 checkpoint in response to DNA mismatches in LUAD for THOC3.

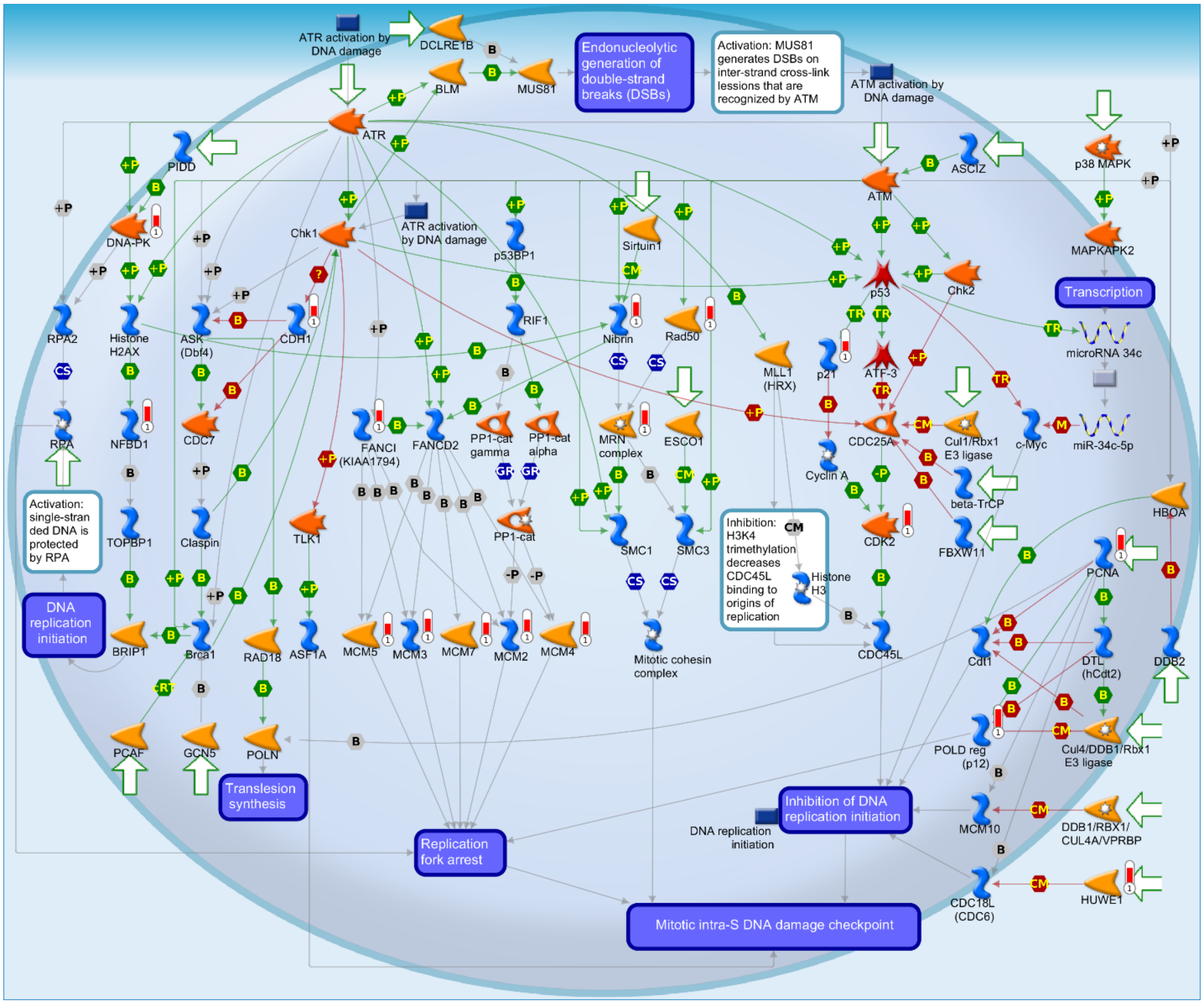

Supplementary Figure 9. Enrichment of the pathway DNA damage: intra-S-phase checkpoint in LUAD for THOC3.

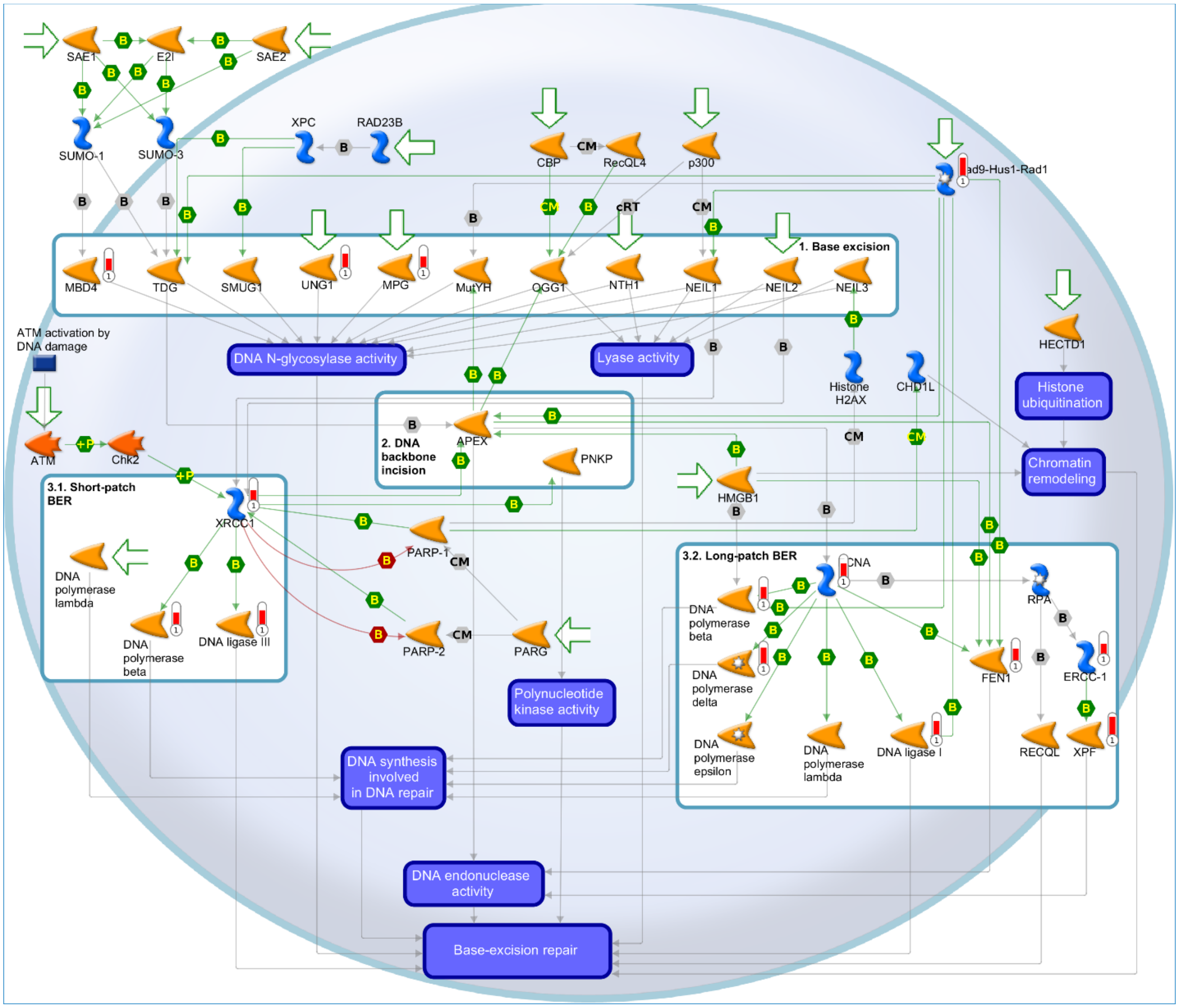

Supplementary Figure 10. Enrichment of the pathway DNA damage: base excision repair (BER) in LUAD for THOC3.

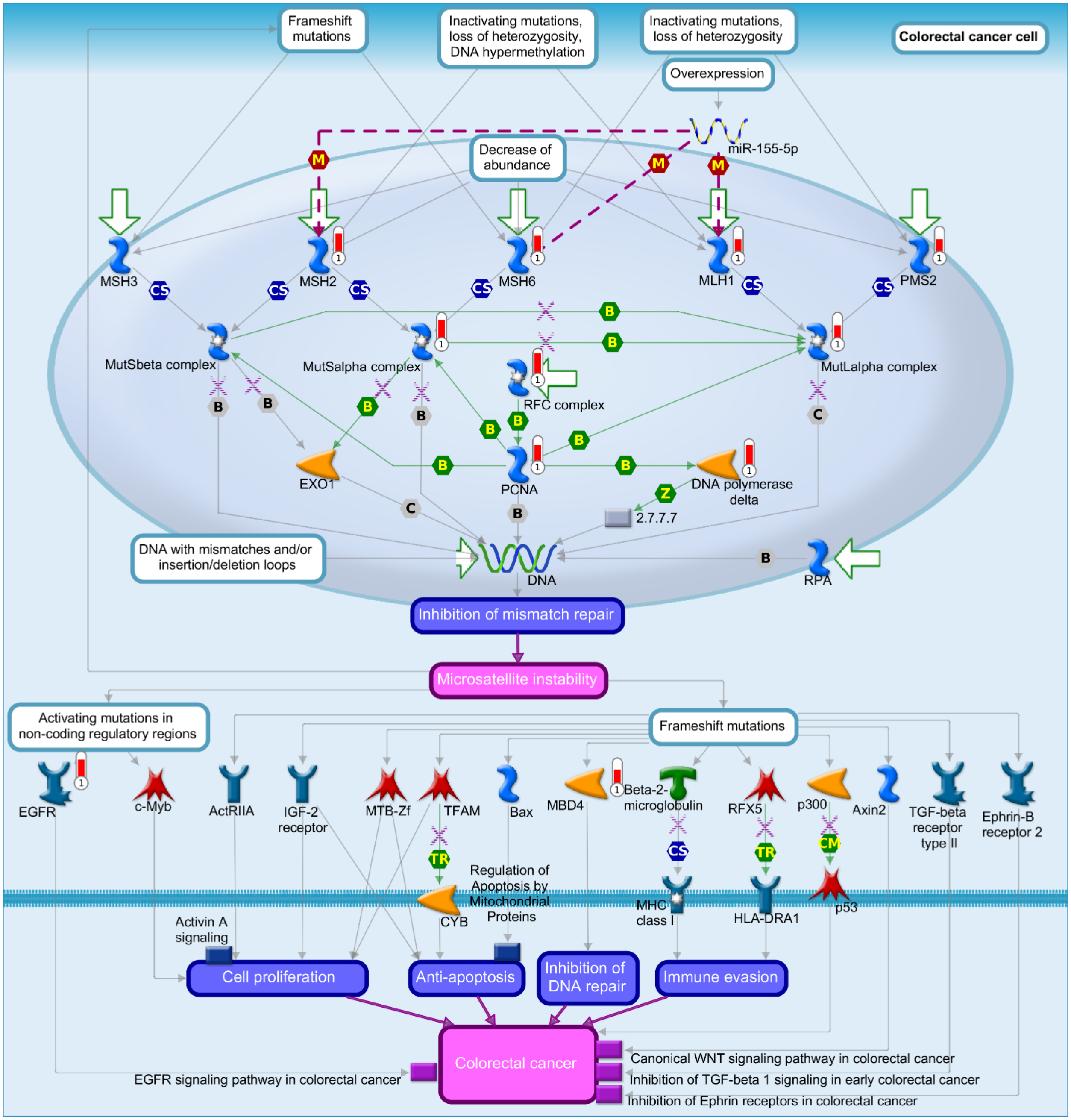

Supplementary Figure 11. Enrichment of the pathway Microsatellite instability (MSI) in colorectal cancer in LUAD for THOC3.

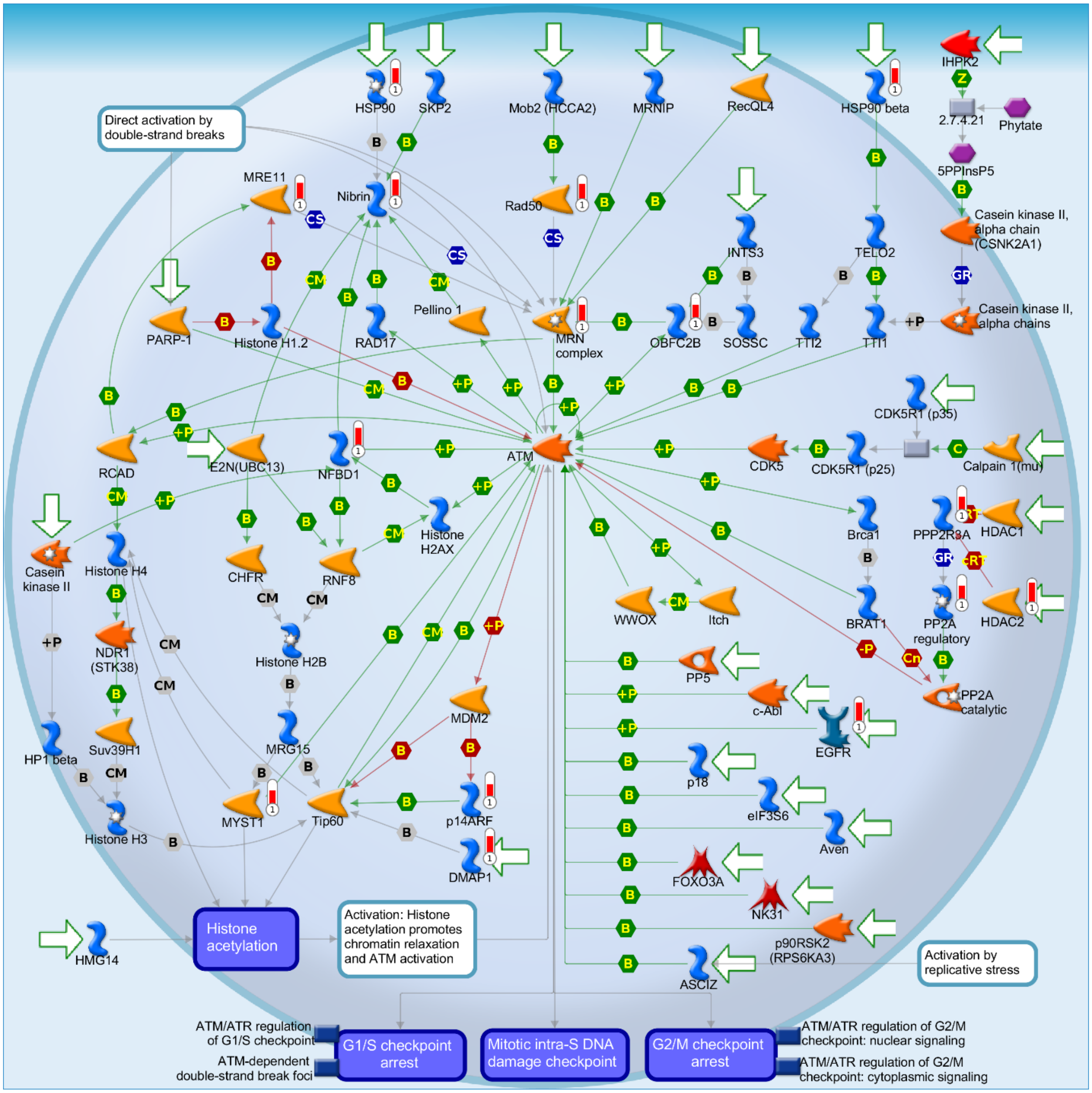

Supplementary Figure 12. Enrichment of the pathway DNA damage: ATM activation by DNA damage in LUAD for THOC3

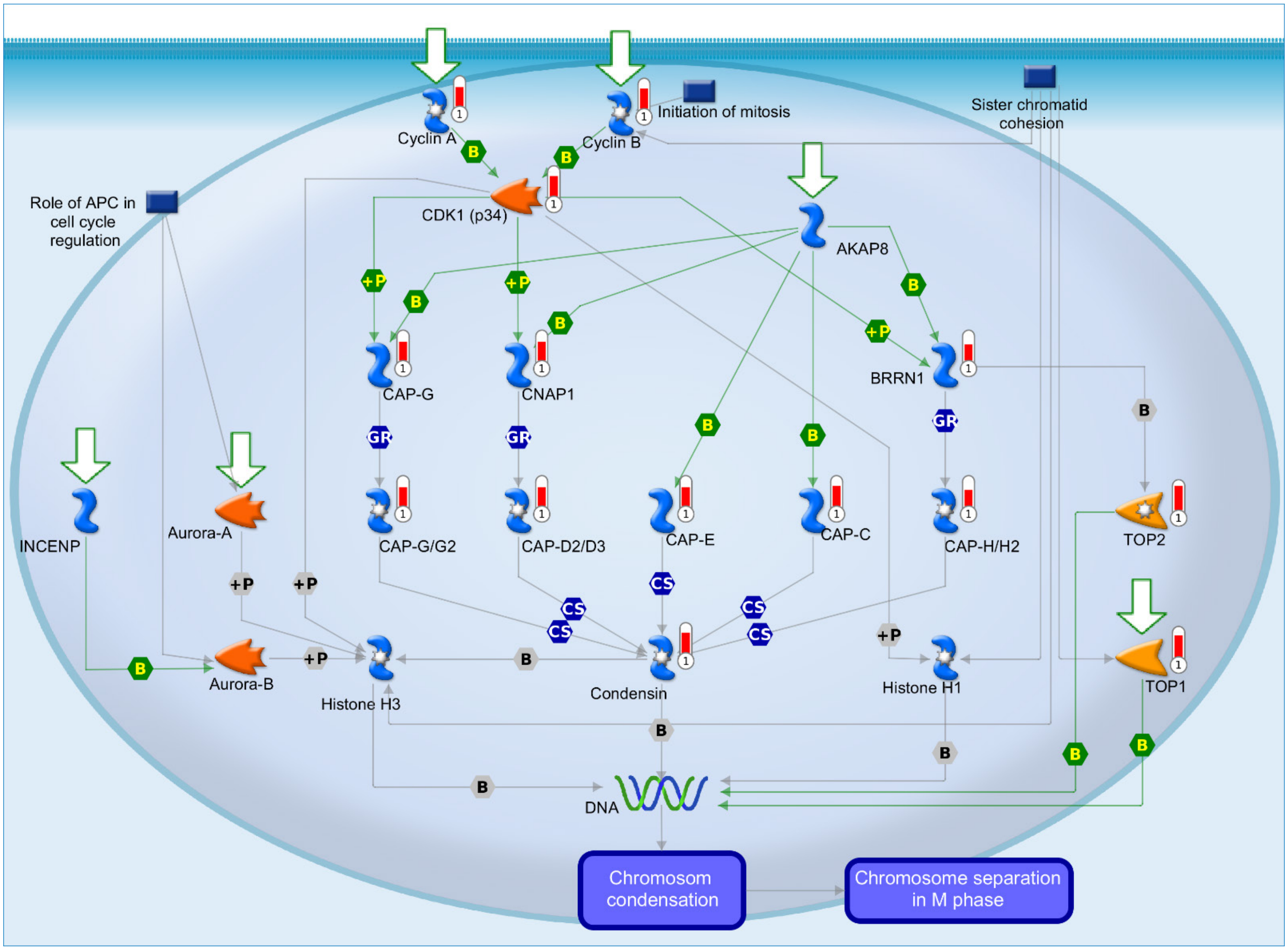

Supplementary Figure 13. Enrichment of the pathway Cell cycle: chromosome condensation in prometaphase in LUAD for THOC7.

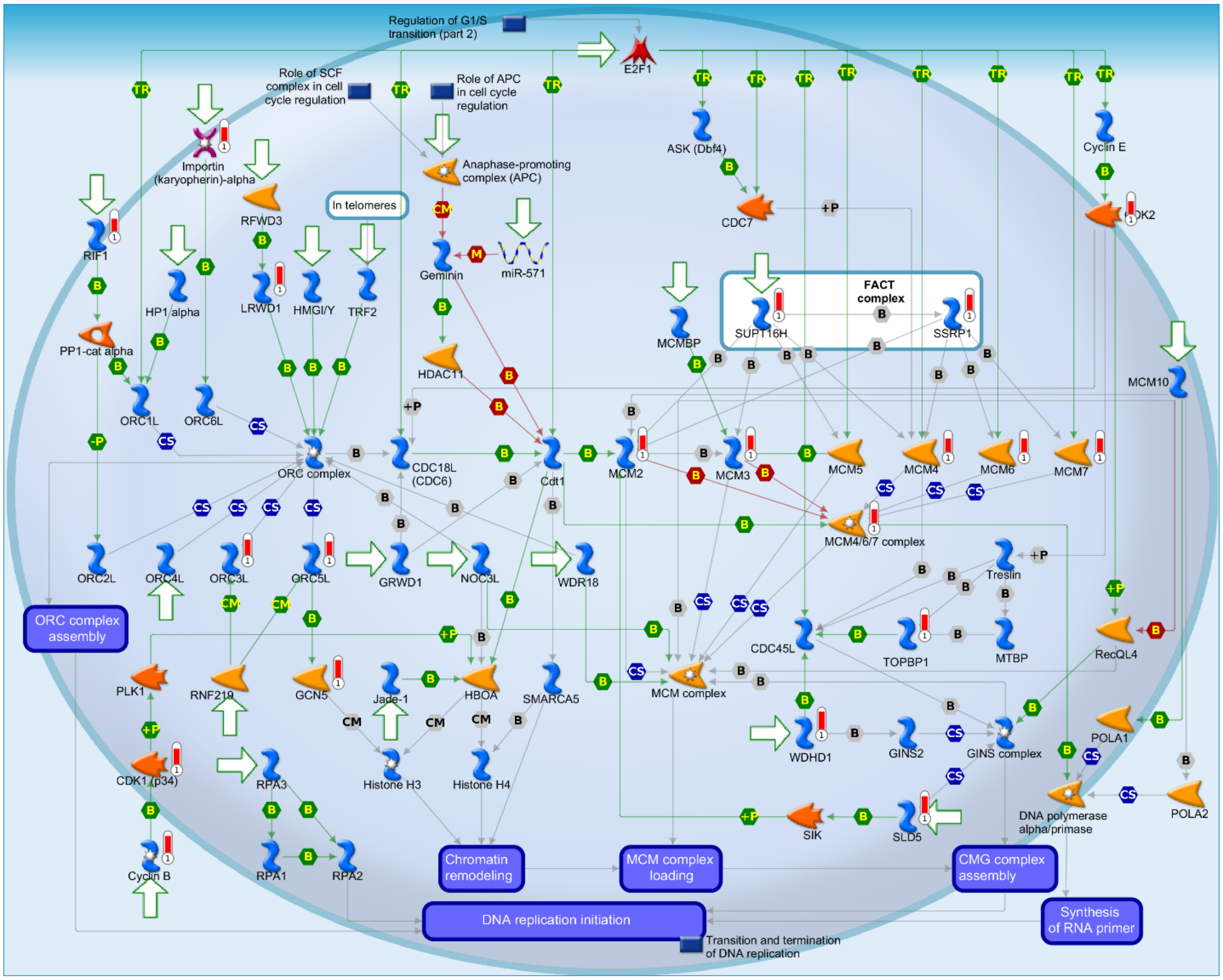

Supplementary Figure 14. Enrichment of the pathway Cell cycle: DNA replication initiation in LUAD for THOC7.

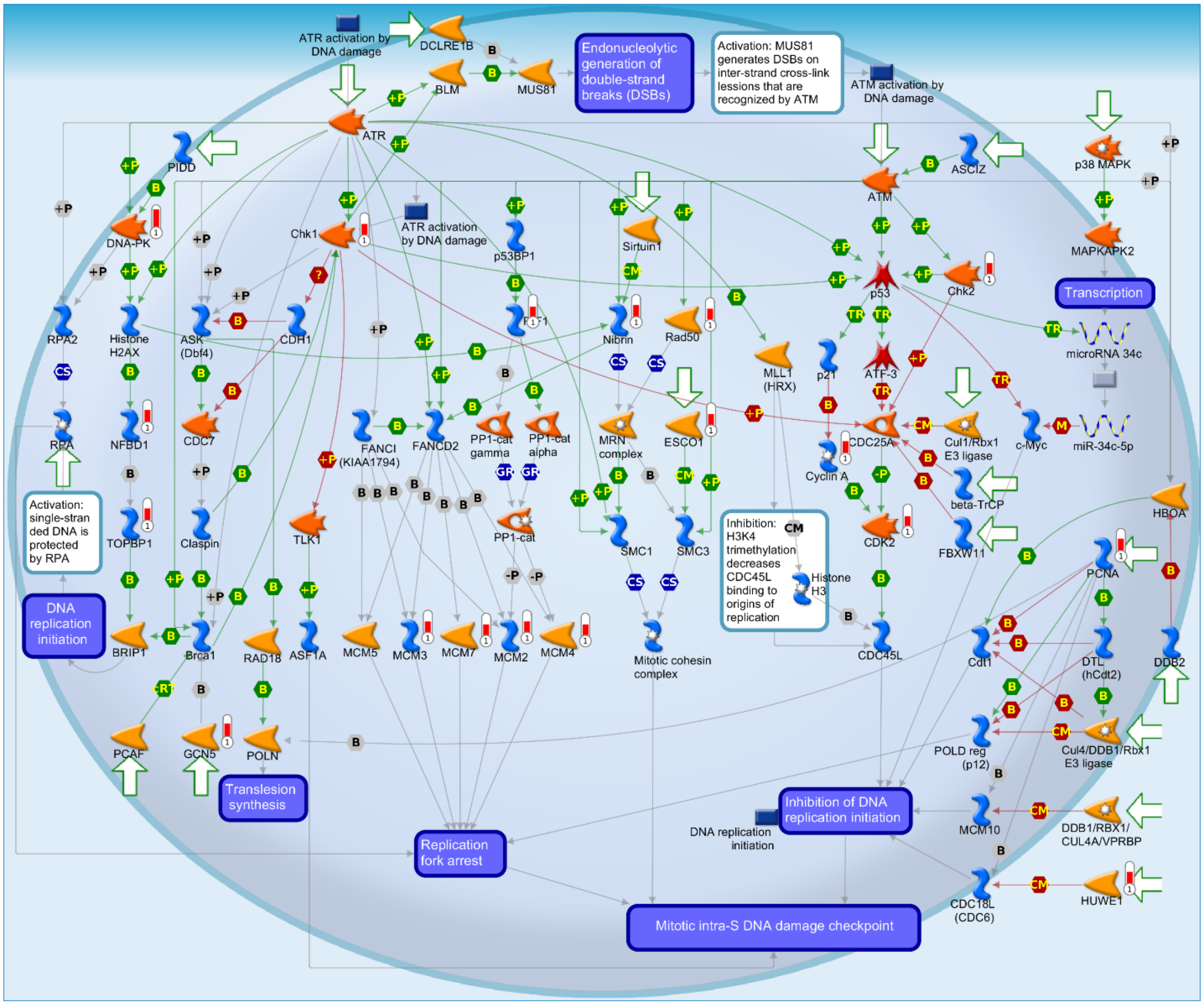

Supplementary Figure 15. Enrichment of the pathway DNA damage: intra-S-phase checkpoint in LUAD for THOC7.

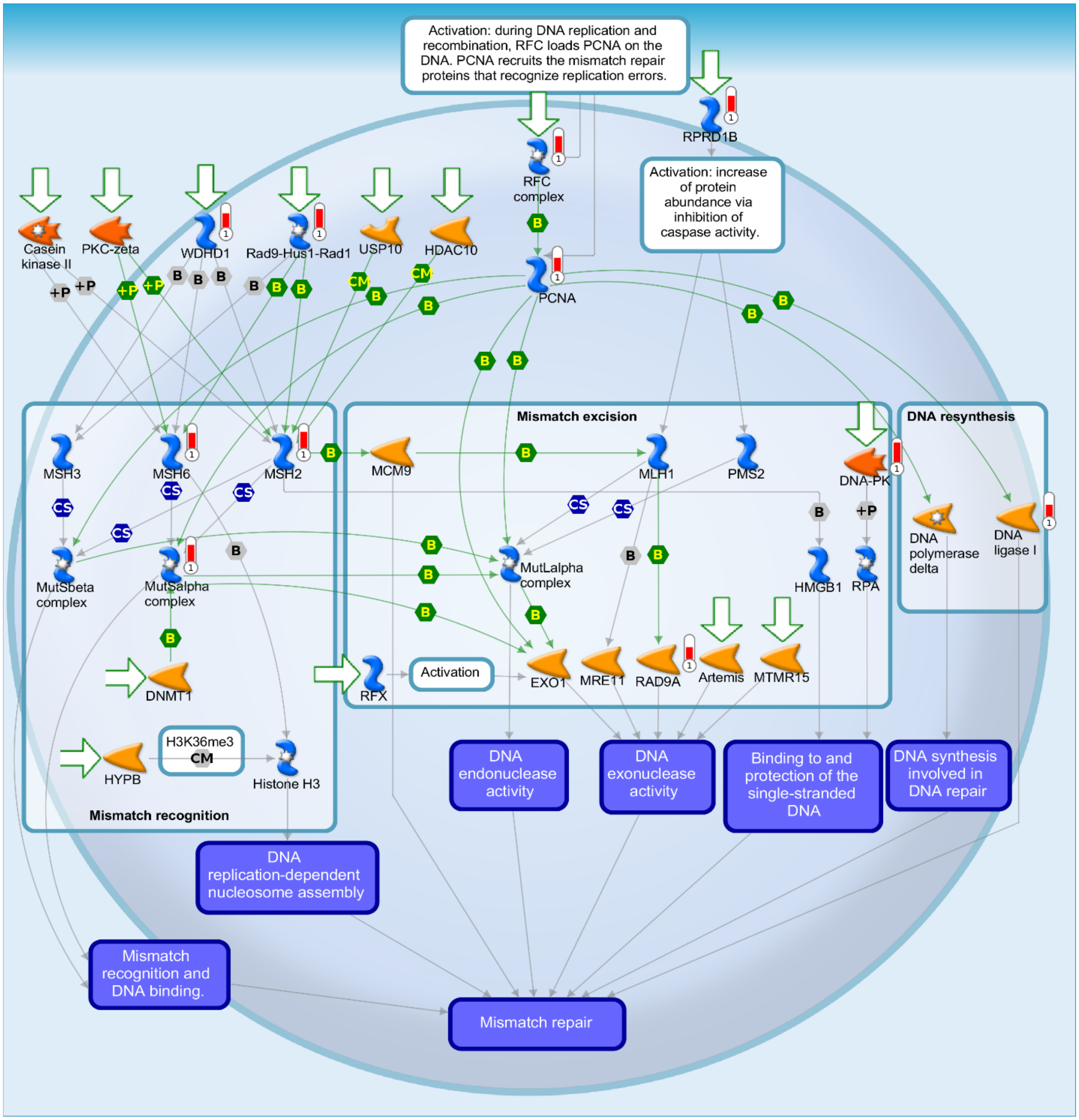

Supplementary Figure 16. Enrichment of the pathway DNA damage: mismatch repair in LUAD for THOC7.

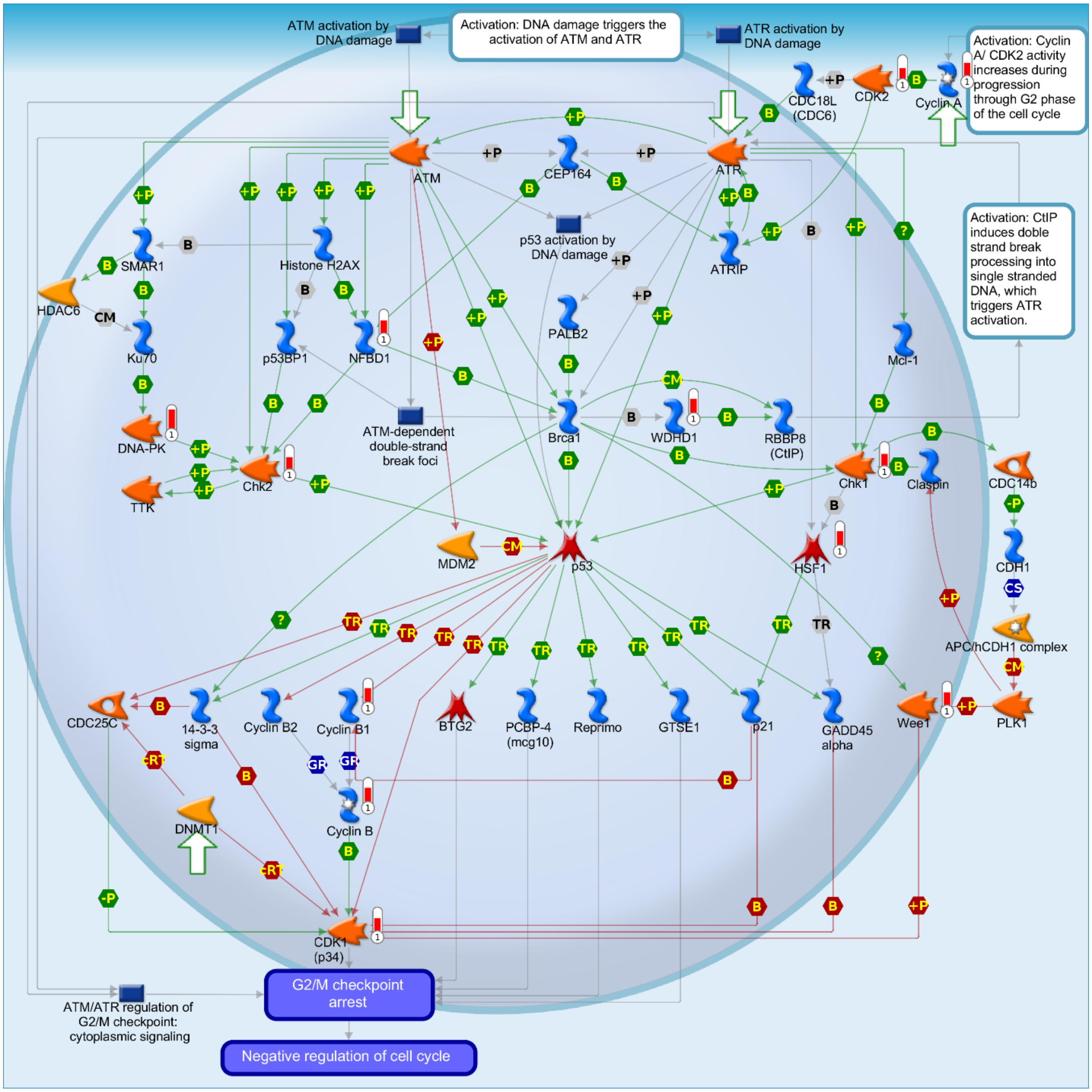

Supplementary Figure 17. Enrichment of the pathway DNA damage: ATM/ATR regulation of G2/M checkpoint nuclear signaling in LUAD for THOC7.

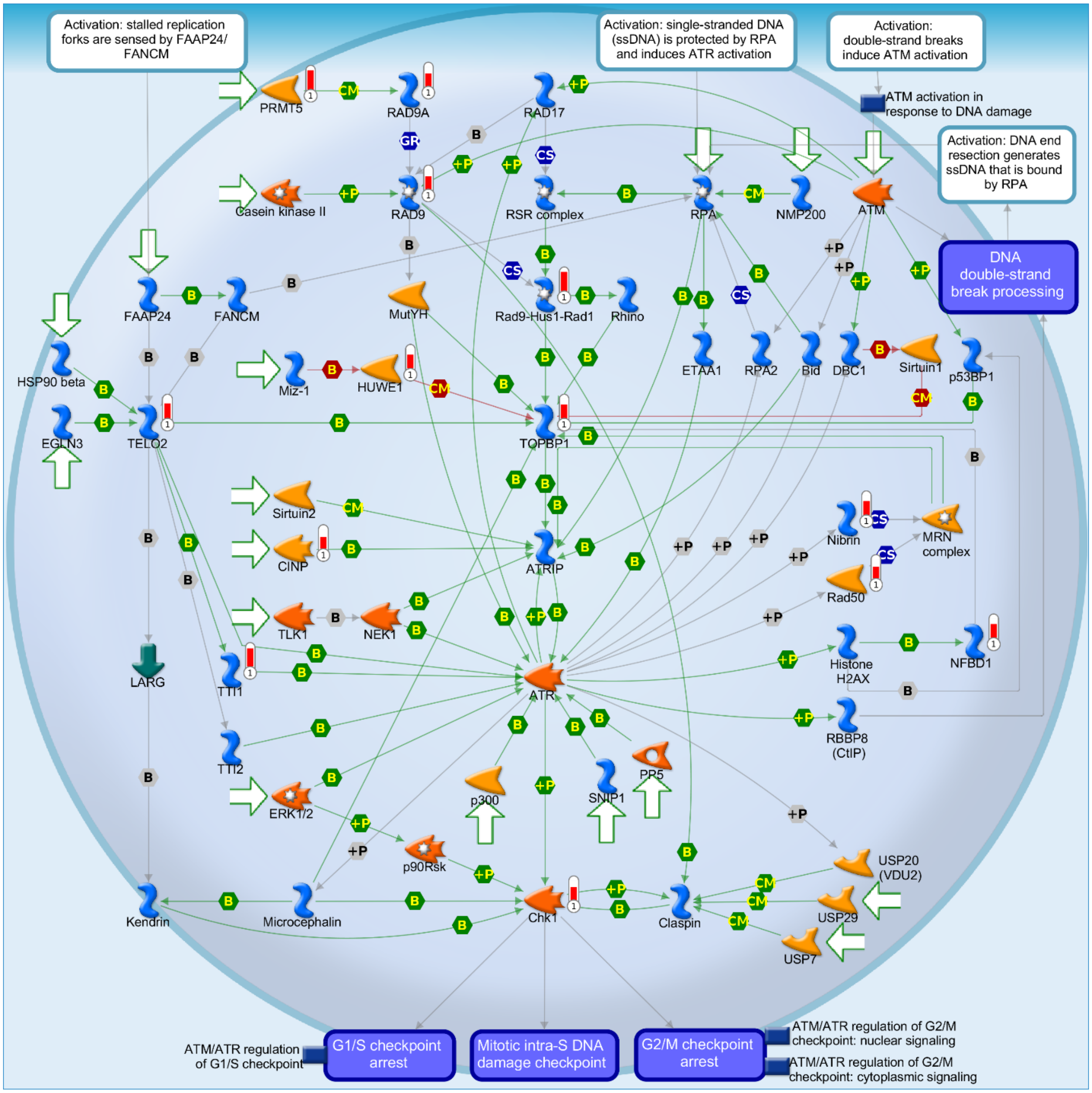

Supplementary Figure 18. Enrichment of the pathway DNA damage: ATR activation by DNA damage in LUAD for THOC7.

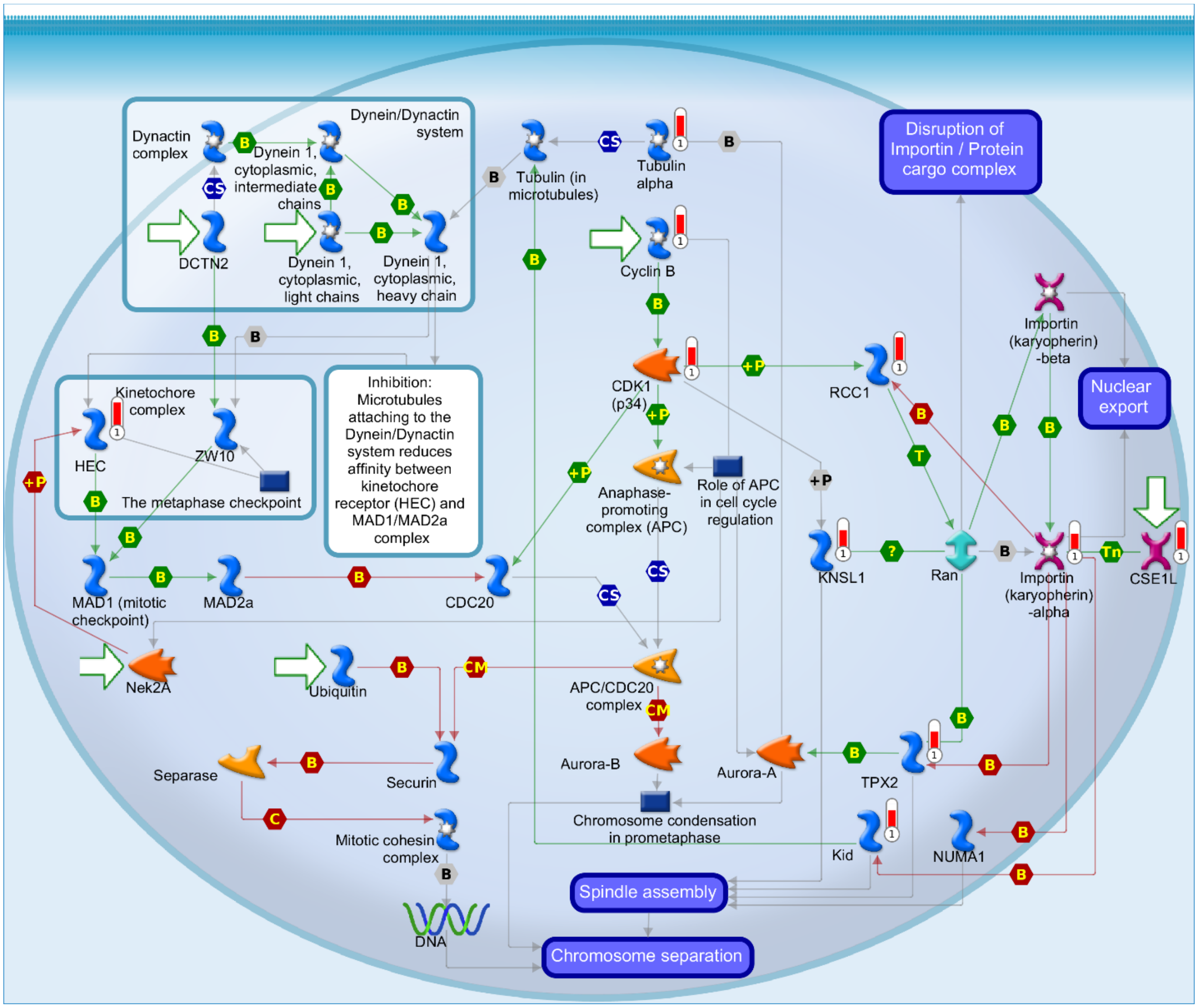

Supplementary Figure 19. Enrichment of the pathway Cell cycle: spindle assembly and chromosome separation in LUAD for THOC7.

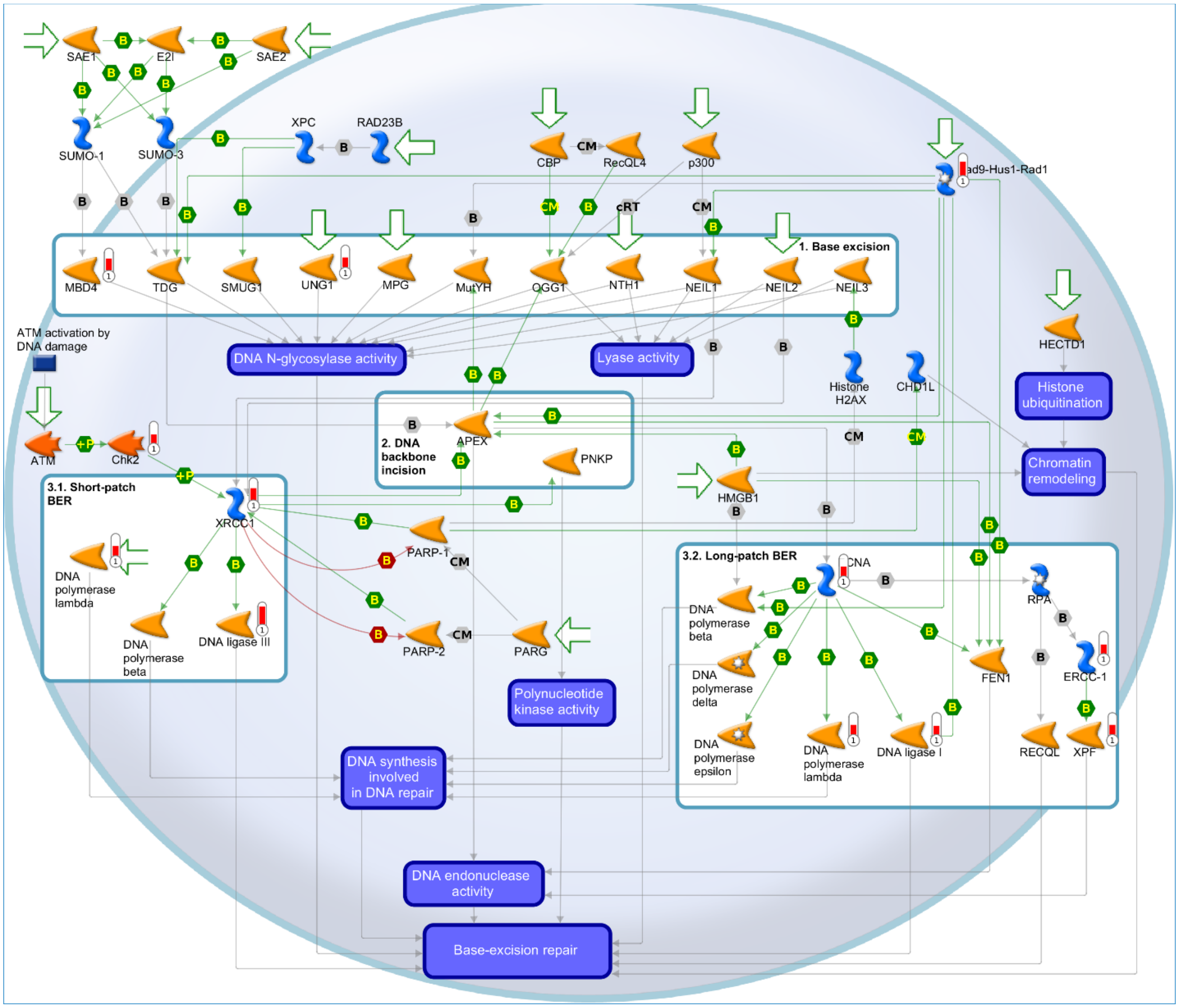

Supplementary Figure 20. Enrichment of the pathway DNA damage: base excision repair (BER) in LUAD for THOC7.

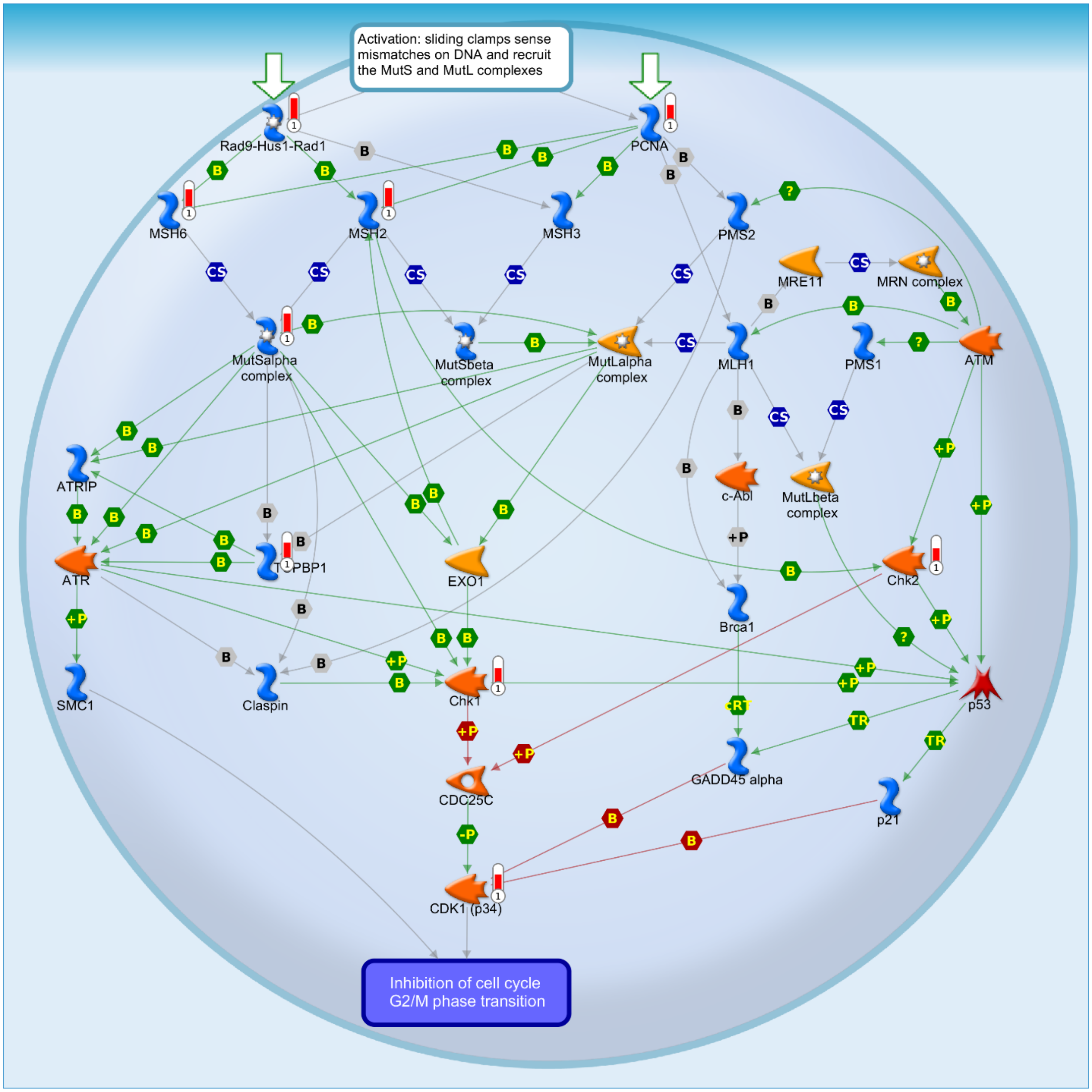

Supplementary Figure 21. Enrichment of the pathway DNA damage_G2 checkpoint in response to DNA mismatches in LUAD for THOC7.

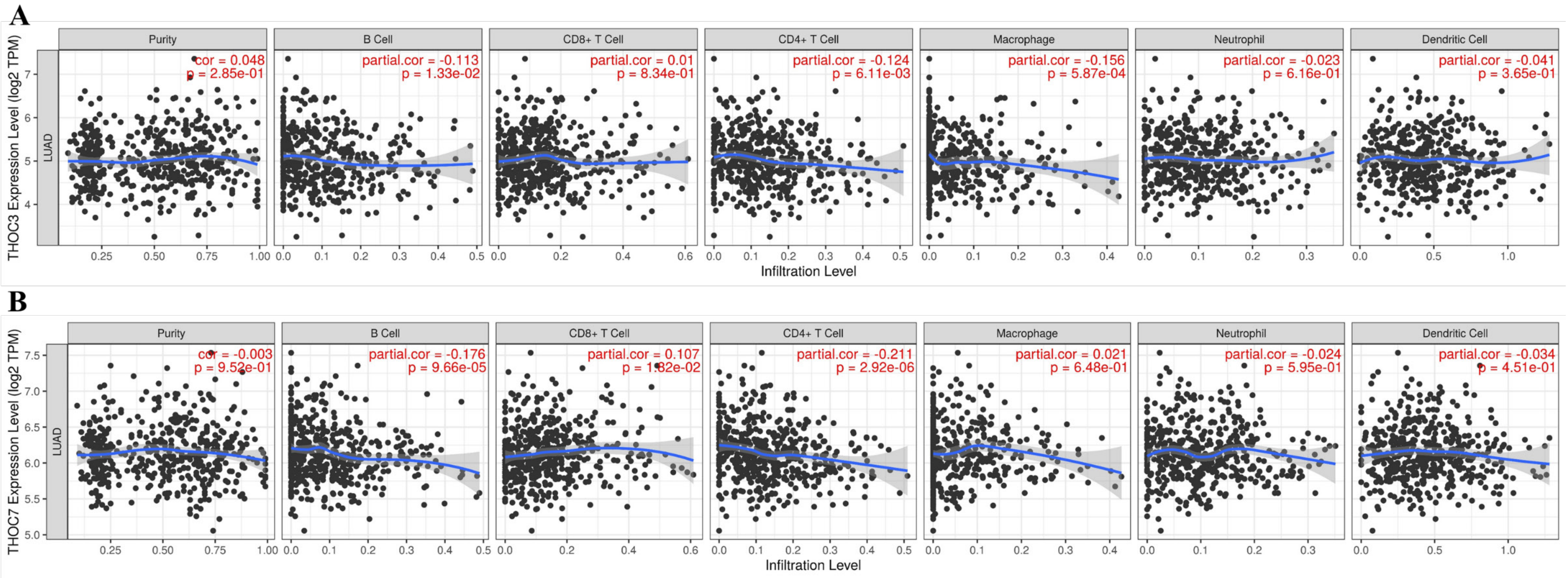

Supplementary Figure 22. Immune infiltration profiles of THOC3 and THOC7 in LUA (A–B) Scatterplots depicting correlations between THOC3 expression (C) and THOC7 expression with immune cell infiltration in LUAD. Analysis was performed across multiple immune subtypes, including B cells, $CD8^+$ T cells, $CD4^+$ T cells, macrophages, neutrophils, and dendritic cells.

Table 1: Pathway analysis of co-expressed genes with THOC3 from the MetaCore database

S No	Maps	pValue	Network Objects from Active Data
**1**	Cell cycle_DNA replication: elongation and termination	1.213E-24	RFC4, TOP2 alpha, WDHD1, PCNA, MCM6, MCM3, RFC1, RFC2, MCM7, TOP1, RFC3, MCM2, POLD reg (p50), FEN1, POLD cat (p125), DNA ligase I, MCM5, RFC5, RFC complex, MCM4, TIPIN, POLD reg (p68), SMARCA3, DCC1, POLD reg (p12), CDK1 (p34), CDC34, CDK2
**2**	Protein folding and maturation_Amyloid precursor protein processing (schema)	4.485E-21	APP-C99, APP-C31, APP-C83 (CTF), APP-CTF delta-short, etaAPP alpha, APP-NCas, Amyloid beta 40, APP-CTF delta-long, betaAPPs, APP, deltaAPPs-80kD, APP-CTF eta, thetaAPPs, APP-P3, alphaAPPs, Amyloid beta, etaAPPs, etaAPP beta, APP-C59 (AICD), deltaAPPs-130kD, APP-CTF theta, APP-Jcasp, Amyloid beta 42
**3**	DNA damage_Mismatch repair	2.864E-17	WDHD1, PCNA, MutSalpha complex, PMS2, DNMT1, MRE11, DNA polymerase delta, RAD9A, MSH2, DNA ligase I, RPRD1B, Rad9-Hus1-Rad1, MLH1, RFC complex, MSH6, MutLalpha complex, DNA-PK
**4**	Cell cycle_Chromosome condensation in prometaphase	1.091E-14	CAP-H/H2, Condensin, CAP-C, CNAP1, CAP-D2/D3, Aurora-B, TOP1, TOP2, BRRN1, CAP-G, CAP-G/G2, CAP-E, CDK1 (p34)
**5**	Cell cycle_DNA replication initiation	3.741E-14	WDHD1, Importin (karyopherin)-alpha, MCM6, MCM3, SSRP1, SMARCA5, MCM7, RPA1, MCM4/6/7 complex, POLA1, MCM2, MCM complex, MCM5, RPA3, SLD5, MCM4, SUPT16H, LRWD1, CDK1 (p34), CDK2
**6**	DNA damage_G2 checkpoint in response to DNA mismatches	1.027E-10	PCNA, MutSalpha complex, PMS2, MRE11, MSH2, p21, Rad9-Hus1-Rad1, MRN complex, MLH1, MSH6, MutLalpha complex, CDK1 (p34)
**7**	DNA damage_Intra S-phase checkpoint	4.860E-10	PCNA, CDH1, MCM3, Rad50, NFBD1, MCM7, MCM2, MCM5, p21, MRN complex, MCM4, FANCI (KIAA1794), Nibrin, HUWE1, POLD reg (p12), DNA-PK, CDK2
**8**	DNA damage_Base excision repair	2.506E-09	PCNA, UNG1, MPG, XRCC1, DNA polymerase delta, DNA polymerase beta, XPF, ERCC-1, FEN1, DNA ligase I, Rad9-Hus1-Rad1, MBD4, DNA ligase III
**9**	Microsatellite instability in colorectal cancer	8.796E-09	PCNA, MutSalpha complex, PMS2, DNA polymerase delta, MSH2, MBD4, MLH1,

			RFC complex, MSH6, EGFR, MutLalpha complex
**10**	DNA damage_ATM activation by DNA damage	1.190E-08	PP2A regulatory, Rad50, MRE11, p14ARF, NFBD1, DMAP1, HDAC2, HSP90, PPP2R3A, OBFC2B, MRN complex, Nibrin, EGFR, HSP90 beta, MYST1
**11**	Microsatellite instability in gastric cancer	1.886E-08	PCNA, MutSalpha complex, PMS2, MSH2, MLH1, RFC complex, p16INK4, MSH6, MutLalpha complex
**12**	DNA damage_Double-strand break repair via homologous recombination	1.284E-07	FIGNL1, WDHD1, PPP4C, MRE11, SMARCA5, NFBD1, RPA1, MRN complex, WDR79, BRD9, Nibrin, CCDC98 (Abraxas), RAP80, CDK1 (p34), CDK2
**13**	DNA damage_ATR activation by DNA damage	1.307E-06	Rad50, PRMT5, NFBD1, RAD9A, Rad9-Hus1-Rad1, MRN complex, Nibrin, HUWE1, HSP90 beta, USP7, RAD9
**14**	DNA damage_ATM-dependent double-strand break foci	1.921E-06	SMARCA5, NFBD1, ENL, HBXAP, MRN complex, BAT3, Mi-2 beta, Nibrin, USP7, CCDC98 (Abraxas), RAP80, KDM2A
**15**	DNA damage_Nucleotide excision repair	1.921E-06	PCNA, RFC1, XRCC1, DNA polymerase delta, XPF, ERCC-1, DNA ligase I, MPP11, RFC complex, DNA ligase III, SUPT16H, USP7
**16**	Cell cycle_The metaphase checkpoint	2.335E-06	PMF1, BUB3, DSN1, Aurora-B, ZW10, BUB1, CDCA1, Zwilch, BUBR1
**17**	Transcription_Negative regulation of HIF1A function	3.136E-06	SART1, MCM3, p14ARF, MCM7, MCM2, MCM5, Elongin B, HSP90, HSP70, HSPA4, HSP90 beta, LAMP2
**18**	Abnormalities in cell cycle in small cell lung cancer (SCLC)	3.847E-06	PCNA, p14ARF, Aurora-B, CKS1, p21, p16INK4, CDK1 (p34), CDK2
**19**	Cell cycle_Sister chromatid cohesion	3.861E-06	PCNA, TOP1, RFC3, PDS5, RFC complex, Stromalins 1/2, DCC1
**20**	dCTP/dUTP metabolism	6.694E-06	AK2, POLA1, DNA polymerase beta, POLD reg (p50), POLD cat (p125), RRM2, TK1, POLG reg, POLD reg (p68), Small RR subunit, AK1, POLD reg (p12)
**21**	Transcription_Sin3 and NuRD in transcription regulation	7.471E-06	Mi-2, MBD3, HDAC2, RBBP4 (RbAp48), Mi-2 beta, SMRT, Sin3B, RAR-alpha/RXR-beta, RARalpha
**22**	Cell cycle_Role of APC in cell cycle regulation	8.617E-06	BUB3, CDH1, Aurora-B, CKS1, BUB1, CDK1 (p34), CDK2, BUBR1
**23**	Cell cycle_Spindle assembly and chromosome separation	1.104E-05	Importin (karyopherin)-alpha, RCC1, KNSL1, Aurora-B, Tubulin alpha, ZW10, CSE1L, CDK1 (p34)
**24**	dATP/dITP metabolism	1.677E-05	8ODP, AK2, POLA1, DNA polymerase beta, POLD reg (p50), POLD cat (p125), Adenosine kinase, RRM2, POLG reg, POLD reg (p68), Small RR subunit, AK1, POLD reg (p12)
**25**	Aberrant B-Raf signaling in melanoma progression	8.890E-05	Rictor, Aurora-B, B-Raf, RHEB2, p21, Nicastrin, HES1, SRp55, CDK1 (p34)

**26**	DNA damage_ATM/ATR regulation of G2/M checkpoint: nuclear signaling	1.015E-04	WDHD1, CDH1, DNMT1, NFBD1, p21, DNA-PK, CDK1 (p34), CDK2
**27**	CFTR folding and maturation (normal and cystic fibrosis)	1.195E-04	Calnexin, HSP90 alpha, HSP70, Aha1, HSP90 beta, FKBP8
**28**	ATP/ITP metabolism	2.799E-04	AK2, POLR1A, Adenosine kinase, RPB5, ENTPD6, RRM2, HPRT, RPB7.0, Small RR subunit, 5'-NT1A, ADAR1, RPB8, AK1
**29**	Immune response_ETV3 affect on CSF1-promoted macrophage differentiation	3.485E-04	DDX20, HDAC2, SMRT, PRIM2A, CDK1 (p34)
**30**	Brca1 and Brca2 in breast cancer	3.485E-04	Rad50, MSH2, MRN complex, MLH1, Nibrin
**31**	DNA damage_Classical NHEJ mechanism of DSBs repair	3.538E-04	PAXIP1L, NFBD1, Aprataxin, TARDBP (TDP43), HDAC2, MRN complex, DNA-PK
**32**	TTP metabolism	4.664E-04	POLA1, UDP, DNA polymerase beta, POLD reg (p50), POLD cat (p125), TK1, POLG reg, POLD reg (p68), POLD reg (p12)
**33**	Possible regulation of HSF-1/ chaperone pathway in Huntington's disease	5.765E-04	HSP90 alpha, HSP90, PLA2, HSP70, HSP90 beta
**34**	Tau dysregulation in Alzheimer disease	5.984E-04	Caspase-2, CAPON, LCMT1, PP2A regulatory, OGT (GlcNAc transferase), TARDBP (TDP43), APP, HSP90, APP-C59 (AICD), Amyloid beta 42
**35**	HSP70 and HSP40-dependent folding in Huntington's disease	1.345E-03	HSP90 alpha, HSP90, HSP70, SGTA, HSP90 beta
**36**	dGTP metabolism	1.401E-03	8ODP, POLA1, DNA polymerase beta, POLD reg (p50), POLD cat (p125), POLG reg, POLD reg (p68), POLD reg (p12)
**37**	Stem cells_Cytotoxic effect of temozolomide on glioblastoma cells	1.933E-03	MutSalpha complex, MSH2, p21, MSH6, MutLalpha complex
**38**	DNA damage_p53 activation by DNA damage	2.079E-03	PP2A regulatory, AATF (Che-1), p21, TTC5 (Strap), USP7, MYST1, PP2C gamma, DNA-PK
**39**	Transcription_Role of heterochromatin protein 1 (HP1) family in transcriptional silencing	2.152E-03	Mi-2, DNMT1, SETDB1, MBD3, HDAC2, CDK1 (p34)
**40**	Cell cycle_Senescence activation pathways	2.233E-03	p14ARF, RHEB2, HSP90, p21, p16INK4, WISp39, CDK2
**41**	The role of aberrations in CDKN2 locus and CDK4 in familial melanoma	2.289E-03	p14ARF, p21, p16INK4, EGFR, MIG6
**42**	Mitogenic action of Estradiol / ESR1 (nuclear) in breast cancer	2.289E-03	CHD8, p21, NCOA3 (pCIP/SRC3), LRP16, CDK2

**43**	Maturation and migration of dendritic cells in skin sensitization	2.450E-03	MHC class II alpha chain, HLA-DRB1, MHC class II, HLA-DRB5, MHC class II beta chain, HLA-DRB
**44**	Cell cycle_Role of Cul1/Rbx1 E3 ligase in cell cycle regulation	2.689E-03	CKS1, p21, CDK1 (p34), CDC34, CDK2
**45**	Transcription_Epigenetic regulation of gene expression	3.056E-03	DNMT1, PRMT5, SETDB1, SMCX, JMJD1A, HDAC2, MYST1
**46**	DNA damage_ATM/ATR regulation of G1/S checkpoint	3.137E-03	PCNA, PP2A regulatory, NFBD1, p21, MRN complex, CDK2
**47**	Apoptosis and survival_Regulation of apoptosis by mitochondrial proteins	3.240E-03	Caspase-2, RAD9A, NOR1, PP2C, Aif, Fis1, TIMM8A, RAD9, Cofilin, CDK2
**48**	Development_H3K36 demethylation in stem cell maintenance	4.147E-03	p14ARF, M33, p21, p16INK4
**49**	Regulation of metabolism_ChREBP signaling	4.918E-03	PYC, PP2A regulatory, OGT (GlcNAc transferase), ACLY, SCD, SMRT, G6PT
**50**	Epigenetic alterations in ovarian cancer	6.644E-03	HNF1-beta, DNMT1, Aurora-B, HDAC2, p21, MLH1, p16INK4, SUZ12

Table 2: Pathway analysis of co-expressed genes with THOC7 from the MetaCore database

S No	Maps	pValue	Network Objects from Active Data
**1**	Cell cycle_DNA replication: elonation and termination	2.684E-17	RFC4, TOP2 alpha, WDHD1, PCNA, MCM6, MCM3, RFC1, RFC2, Cyclin A, Chk1, MCM7, TOP1, RFC3, MCM2, DNA ligase I, RFC5, RFC complex, MCM4, TIPIN, SMARCA3, CDK1 (p34), CDC34, CDK2
**2**	Cell cycle_Chromosome condensation in prometaphase	7.157E-16	CAP-H/H2, Condensin, CAP-C, Cyclin A, CNAP1, CAP-D2/D3, TOP1, Cyclin B, TOP2, BRRN1, CAP-G, CAP-G/G2, CAP-E, CDK1 (p34)
**3**	Cell cycle_DNA replication initiation	1.916E-13	WDHD1, Importin (karyopherin)-alpha, MCM6, MCM3, SSRP1, RIF1, MCM7, ORC5L, Cyclin B, MCM4/6/7 complex, MCM2, TOPBP1, SLD5, MCM4, SUPT16H, ORC3L, LRWD1, CDK1 (p34), CDK2, GCN5
**4**	DNA damage_Intra S-phase checkpoint	2.224E-10	ESCO1, PCNA, Chk2, MCM3, Cyclin A, RIF1, Rad50, Chk1, NFBD1, MCM7, MCM2, TOPBP1, MCM4, Nibrin, HUWE1, DNA-PK, CDK2, GCN5
**5**	DNA damage_Base excision repair	7.149E-09	PCNA, UNG1, Chk2, XRCC1, RAD23B, DNA polymerase beta, XPF, ERCC-1, DNA ligase I, Rad9-Hus1-Rad1, MBD4, DNA ligase III, DNA polymerase lambda
**6**	DNA damage_Mismatch repair	1.044E-08	WDHD1, PCNA, MutSalpha complex, RAD9A, MSH2, DNA ligase I, RPRD1B, Rad9-Hus1-Rad1, RFC complex, MSH6, DNA-PK
**7**	DNA damage_ATM/ATR regulation of G2/M checkpoint: nuclear signaling	4.252E-08	WDHD1, HSF1, Chk2, Cyclin A, Chk1, NFBD1, Wee1, Cyclin B, Cyclin B1, DNA-PK, CDK1 (p34), CDK2
**8**	DNA damage_ATR activation by DNA damage	4.659E-08	TELO2, Rad50, Chk1, PRMT5, NFBD1, RAD9A, CINP, Rad9-Hus1-Rad1, TOPBP1, TTI1, Nibrin, HUWE1, RAD9
**9**	Cell cycle_Spindle assembly and chromosome separation	1.935E-07	Importin (karyopherin)-alpha, RCC1, KNSL1, HEC, Kid, Tubulin alpha, Cyclin B, TPX2, CSE1L, CDK1 (p34)
**10**	DNA damage_G2 checkpoint in response to DNA mismatches	8.830E-07	PCNA, MutSalpha complex, Chk2, Chk1, MSH2, Rad9-Hus1-Rad1, TOPBP1, MSH6, CDK1 (p34)
**11**	Transcription_Role of heterochromatin protein 1 (HP1) family in transcriptional silencing	1.403E-06	TIF1-beta, Mi-2, SETDB1, MBD3, HP1 beta, HDAC2, Cyclin A2, HP1 gamma, HP1, CDK1 (p34)

**12**	Transcription_Sin3 and NuRD in transcription regulation	1.794E-06	Mi-2, MBD3, p66beta, HDAC2, PSF, RBBP4 (RbAp48), Mi-2 beta, Sin3B, RAR-alpha/RXR-beta, RARalpha
**13**	DNA damage_Double-strand break repair via homologous recombination	2.276E-06	FIGNL1, WDHD1, PPP4C, RIF1, RMI1, NFBD1, AUNIP, RecQ5, TOPBP1, WDR79, RAD54L, Nibrin, CDK1 (p34), CDK2
**14**	Cell cycle_Sister chromatid cohesion	6.856E-06	ESCO1, PCNA, TOP1, RFC3, PDS5, RFC complex, Stromalins 1/2
**15**	Abnormalities in cell cycle in small cell lung cancer (SCLC)	7.312E-06	PCNA, Cyclin A, p14ARF, CKS1, Cyclin B1, p16INK4, CDK1 (p34), CDK2
**16**	Microsatellite instability in colorectal cancer	2.626E-05	PCNA, MutSalpha complex, MSH2, MBD4, RFC complex, MSH6, EGFR, c-Myb
**17**	DNA damage_Nucleotide excision repair	2.774E-05	PCNA, RFC1, XRCC1, RAD23B, XPF, ERCC-1, DNA ligase I, MPP11, RFC complex, DNA ligase III, SUPT16H
**18**	Cell cycle_Initiation of mitosis	3.300E-05	Nucleolin, KNSL1, Wee1, Cyclin B1, MAT1, CDK1 (p34), CDK7
**19**	DNA damage_ATM activation by DNA damage	5.595E-05	TELO2, Rad50, p14ARF, NFBD1, HP1 beta, HDAC2, HSP90, OBFC2B, TTI1, Nibrin, EGFR
**20**	Proteolysis_SUMOylation pathway	7.559E-05	TIF1-beta, MMS21, ZNF451, TRIM33, p14ARF, UHRF2, HDAC2, PIAS3
**21**	Possible regulation of HSF-1/ chaperone pathway in Huntington's disease	8.530E-05	HSF1, HSP90 alpha, HSP90, PLA2, HSP70, p23 co-chaperone
**22**	Cell cycle_Nucleocytoplasmic transport of CDK/Cyclins	1.044E-04	Importin (karyopherin)-alpha, Cyclin A, Cyclin B1, CDK1 (p34), CDK2
**23**	Development_Negative regulation of WNT/Beta-catenin signaling in the nucleus	1.050E-04	P15RS, NF-AT3(NFATC4), TRIM33, BCL9/B9L, Calcineurin A (catalytic), CHD8, TLE, HDAC2, RUVBL2, Kaiso, Frizzled, 14-3-3
**24**	Microsatellite instability in gastric cancer	1.136E-04	PCNA, MutSalpha complex, MSH2, RFC complex, p16INK4, MSH6
**25**	Development_H3K9 demethylases in pluripotency maintenance of stem cells	1.136E-04	RING2, TIF1-beta, SETDB1, JMJD1A, HP1, LBP9
**26**	Cell cycle_Role of 14-3-3 proteins in cell cycle regulation	1.136E-04	Chk2, Chk1, Wee1, HUS1, 14-3-3 zeta/delta, CDK1 (p34)
**27**	Cell cycle_Role of APC in cell cycle regulation	1.392E-04	Cyclin A, Kid, Cyclin B, CKS1, BUB1, CDK1 (p34), CDK2
**28**	DNA damage_ATM-dependent double-strand break foci	1.470E-04	RING2, TIF1-beta, NFBD1, BAZ1A, HBXAP, BAT3, Mi-2 beta, Nibrin, HP1 gamma, GCN5
**29**	DNA damage_DNA-damage-induced responses	2.033E-04	Chk2, Chk1, NFBD1, DNA-PK
**30**	Transcription_Negative regulation of HIF1A function	2.145E-04	SART1, MCM3, p14ARF, MCM7, MCM2, Elongin B, HSP90, HSP70, RUVBL2, Elongin C

**31**	Development_WNT/Beta-catenin signaling in the nucleus	4.360E-04	MED1, PYGO2, BCL9/B9L, TLE, Kaiso, Frizzled, UBR5, GCN5, RUVBL1
**32**	Development_Positive regulation of WNT/Beta-catenin signaling in the cytoplasm	4.778E-04	TGT, COX-2 (PTGS2), Bcl-9, BIG1, TGIF, 14-3-3 zeta/delta, Frizzled, CDK1 (p34), FAK1, 14-3-3
**33**	Cell cycle_Role of Cul1/Rbx1 E3 ligase in cell cycle regulation	5.802E-04	Chk1, Wee1, CKS1, CDK1 (p34), CDC34, CDK2
**34**	DNA damage_Classical NHEJ mechanism of DSBs repair	5.96 6E-04	RIF1, PAXIP1L, NFBD1, Aprataxin, HDAC2, DNA polymerase lambda, DNA-PK
**35**	Development_Transcription factors in segregation of hepatocytic lineage	7.02 8E-04	HNF1-beta, p14ARF, CPSM, Activin A, p16INK4, Activin
**36**	Signal transduction_Glucocorticoid receptor signaling	7.85 2E-04	Keratin 17, MED1, TFB2M, HSP90, Karyopherin alpha 2, FKBP4, TFB1M, Oct-1
**37**	Development_Positive regulation of WNT/Beta-catenin signaling in the nucleus	9.686E-04	SOX4, BCL9/B9L, FOXK1, TLE, HDAC2, RUVBL2, NCOA2 (GRIP1/TIF2), Frizzled, UBR5
**38**	Cell cycle_Role of Nek in cell cycle regulation	1.007E-03	RCC1, HEC, Tubulin alpha, Cyclin B1, TPX2, CDK1 (p34)
**39**	CFTR folding and maturation (normal and cystic fibrosis)	1.631E-03	HSP90 alpha, Csp, HSP70, HSPBP1, p23 co-chaperone
**40**	Transcription_Mechanism of activation of the transcription of Retinoid-target genes	1.631E-03	MED1, CRABP2, NCOA3 (pCIP/SRC3), NCOA2 (GRIP1/TIF2), RARalpha
**41**	Cell cycle_The metaphase checkpoint	1.910E-03	SPBC25, CENP-B, HEC, BUB1, HP1 gamma, CDCA1
**42**	HSP70 and HSP40-dependent folding in Huntington's disease	1.977E-03	HSP90 alpha, HSP90, HSP70, SGTA, HSPA1A
**43**	ATP/ITP metabolism	2.044E-03	POLR1B, POLR1A, RPA39, RPB5, NDPK A, ADSS, HPRT, RPB7.0, 5'-NT1A, ADAR1, RRM1, RPB8
**44**	Transcription_CoREST complex-mediated epigenetic gene silencing	2.209E-03	BAF57, ZNF217, HDAC2, EHMT1, BAF170, c-Myb
**45**	DNA damage_ATM/ATR regulation of G2/M checkpoint: cytoplasmic signaling	2.609E-03	Chk2, Chk1, PARN, UBE2C, Cyclin B1, CDK1 (p34), 14-3-3
**46**	Signal transduction_mTORC2 upstream signaling	2.671E-03	TELO2, Rictor, HSP70, TTI1, GBL, EGFR, SIN1, Frizzled
**47**	Mitogenic action of Estradiol / ESR1 (nuclear) in breast cancer	3.337E-03	CAD, CHD8, NCOA3 (pCIP/SRC3), CDK2, Oct-1
**48**	Signal transduction_AKT(PKB) activation	3.551E-03	Rictor, Cyclin A, SETDB1, HSP90, GBL, EGFR, SIN1, CDK2
**49**	Cell cycle_Senescence activation pathways	3.628E-03	Chk2, Chk1, p14ARF, RHEB2, HSP90, p16INK4, CDK2
**50**	Aberrant B-Raf signaling in melanoma progression	4.027E-03	Rictor, B-Raf, RHEB2, HES1, MAP2, SRp55, CDK1 (p34)
